# Aberration in DNA Methylation in B-Cell Lymphomas Has a Complex Origin and Increases with Disease Severity

**DOI:** 10.1371/journal.pgen.1003137

**Published:** 2013-01-10

**Authors:** Subhajyoti De, Rita Shaknovich, Markus Riester, Olivier Elemento, Huimin Geng, Matthias Kormaksson, Yanwen Jiang, Bruce Woolcock, Nathalie Johnson, Jose M. Polo, Leandro Cerchietti, Randy D. Gascoyne, Ari Melnick, Franziska Michor

**Affiliations:** 1 Department of Medicine, University of Colorado School of Medicine, Aurora, Colorado, United States of America; 2 Molecular Oncology Program, University of Colorado Cancer Center, Aurora, Colorado, United States of America; 3 Division of Hematology/Oncology, Department of Medicine, Weill Cornell Medical College, New York, New York, United States of America; 4 Division of Immunopathology, Department of Pathology, Weill Cornell Medical College, New York, New York, United States of America; 5 Department of Biostatistics and Computational Biology, Dana-Farber Cancer Institute, Boston, Massachusetts, United States of America; 6 Department of Biostatistics, Harvard School of Public Health, Boston, Massachusetts, United States of America; 7 Institute for Computational Biomedicine, Weill Cornell Medical College, New York, New York, United States of America; 8 Division of Biostatistics and Epidemiology, Department of Public Health, Weill Cornell Medical College, New York, New York, United States of America; 9 Centre for Lymphoid Cancers and Departments of Pathology and Experimental Therapeutics, British Columbia Cancer Agency, British Columbia Cancer Research Centre, Vancouver, British Columbia, Canada; 10 Monash Immunology and Stem Cell Laboratories, Monash University, Clayton, Australia; Cincinnati Children's Hospital Medical Center, United States of America

## Abstract

Despite mounting evidence that epigenetic abnormalities play a key role in cancer biology, their contributions to the malignant phenotype remain poorly understood. Here we studied genome-wide DNA methylation in normal B-cell populations and subtypes of B-cell non-Hodgkin lymphoma: follicular lymphoma and diffuse large B-cell lymphomas. These lymphomas display striking and progressive intra-tumor heterogeneity and also inter-patient heterogeneity in their cytosine methylation patterns. Epigenetic heterogeneity is initiated in normal germinal center B-cells, increases markedly with disease aggressiveness, and is associated with unfavorable clinical outcome. Moreover, patterns of abnormal methylation vary depending upon chromosomal regions, gene density and the status of neighboring genes. DNA methylation abnormalities arise via two distinct processes: i) lymphomagenic transcriptional regulators perturb promoter DNA methylation in a target gene-specific manner, and ii) aberrant epigenetic states tend to spread to neighboring promoters in the absence of CTCF insulator binding sites.

## Author Summary

Follicular lymphomas and diffuse large B-cell lymphomas are the most common non-Hodgkin lymphomas. Although these diseases share many mutant alleles, the underlying cause of the different phenotypes remains unclear. We show that direct comparison of DNA methylation patterning provides insights about gene deregulation during lymphomagenesis and explains the nature of the different clinical behavior.

## Introduction

**Figure 1:**
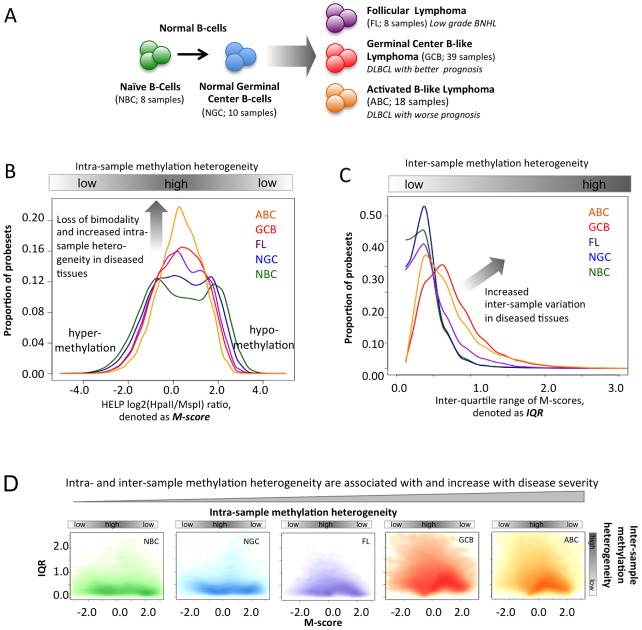
Methylation variation in normal and lymphoma samples. (A) Summary of the normal and lymphoma samples used in this study. (B) Histogram representation of DNA methylation score (M-score, horizontal axis) and frequency (vertical axis). Positive M-scores represent hypo-methylation while negative scores represent hyper-methylation. The DNA methylation distributions of samples are shown using the same color code as in panel A. The methylation patterns of NBC are bimodal, where the positive node represents hypo-methylation and the negative node represents hyper-methylation. The proportion of promoters with intermediate M-score (around zero), which represents high intra-sample variation, increases for lymphoma categories with increased disease severity. (C) The histogram represents the frequency distribution of inter-quartile ranges (IQR) of the M-scores per probeset for normal and diseased samples. The vertical axis represents the frequency of probesets and the horizontal axis represents the IQR. High IQR values indicate high inter-sample variation, and the proportion of such promoters increases for lymphoma categories with increased disease severity. (D) The scatter plot reflects the joint distribution of M-scores and IQR, which represent intra- and inter-sample variation, respectively, per probeset for normal B-cells and lymphoma categories. The color intensity is proportional to the density of points on the graph. High inter-sample variation is also associated with high intra-sample variation. The distribution of points becomes progressively broader and more smear-like in lymphoma samples vs. normal B-cells. The colors are the same as in (A).

Follicular lymphomas (FLs) and diffuse large B-cell lymphomas (DLBCLs) are the most common non-Hodgkin lymphomas [1]. Follicular lymphomas represent a spectrum from low- to high-grade tumors and, while predominantly diagnosed as indolent tumors, progress to more aggressive lymphomas like DLBCL over the course of several years [2]. DLBCLs are high-grade tumors that are sub-classified based on gene expression profiling into a typically chemo-responsive germinal center B-like (GCB) subtype and a more refractory activated B-like (ABC) subtype (Figure 1A) [3]. Although FL and DLBCL have markedly distinct clinical phenotypes, they both originate from mature B-cells transiting the germinal center (GC) reaction. When resting naïve B-cells are activated by exposure to T-cell dependent antigens, they migrate within lymphoid follicles and initiate massive clonal expansion while simultaneously undergoing somatic hypermutation and class switch recombination. Genetic defects arising as a byproduct of this immunoglobulin affinity maturation process are believed to give rise to FLs and DLBCLs [4]. Consistent with this hypothesis, genomic resequencing studies identified a large number of mutations occurring in FL and DLBCL. While it is known that FLs accumulate new mutations as they progress, the underlying cause of the different phenotype of *de novo* FL and DLBCL, which share many of the same mutant alleles, remains unclear. Emerging data suggest that epigenetic gene regulation through cytosine methylation is perturbed in FLs and DLBCLs, yet very little is known about how aberrant DNA methylation contributes to the disease phenotype, the genomic features of epigenetic defects in these tumor types, and mechanisms through which these defects occur. Recently we demonstrated that DNA methylation patterning plays a key role in hematopoietic development [5] and that DNA methylation and expression signatures define molecular subtypes of diffuse large B-cell lymphomas [6]. Here, we hypothesized that direct comparison of DNA methylation patterning in normal B-cells, FLs and DLBCLs would provide clues about gene deregulation during lymphomagenesis and explain the nature of the different clinical behavior of these lymphoma subtypes.

## Results/Discussion

### DNA methylation heterogeneity is associated with increasing disease aggressiveness

We examined the DNA methylation profiles of normal naïve B-cells (NBC, 8 samples), normal germinal center B-cells (NGC, 10 samples), follicular lymphomas (FL, 8 samples), germinal center B-like DLBCLs (GCB, 39 samples), and activated B-like DLBCLs (ABC, 18 samples) (Figure 1A, Methods and Text S1, Module 1; ) using the HELP assay [7] and custom-designed NimbleGen microarrays with probesets representing >50,000 CpGs corresponding to regulatory regions of roughly 14,000 human genes. In the HELP assay, the normalized array signal intensity corresponds to the degree of methylation associated with each probeset (Methods, [6], [8]). For any given probeset, a positive or negative normalized signal intensity indicates that the respective CpGs are either unmethylated or methylated (Figure S4). In contrast, intermediate probeset signal intensity indicates that a fraction of cells within the sample are unmethylated while others are methylated, thus reflecting the heterogeneity of methylation. We performed technical validation for the HELP array and validated DNA methylation profiles of six DLBCL samples using orthogonal base-pair resolution quantitative bisulfite sequencing based assays: ERRBS and MassARRAY assays (Text S1, Module 1 and Figures S5, S6, S7, S8). Overall mapping of probesets according to their positions along the human chromosomes indicated that sites of hypo- and hyper-methylation were distributed across all chromosomes in both normal and lymphoma samples (Text S1, Module 1, and Figure S9). However, we noted a higher abundance of intermediate methylation states in lymphomas and hypothesized that epigenetic heterogeneity might contribute to the clinical features of the disease.

In order to address this question we derived two parameters:

- The “M-score”, a measurement of intra-sample DNA methylation heterogeneity. The M-score specifically reflects the degree of methylation at a given probeset and thus the uniformity with which specific CpGs are methylated or unmethylated; an intermediate score (around zero) reflects the presence of balanced hypo- and hyper-methylated CpGs within the cells of a sample and thus high intra-sample variation (Figure 1B).
- The inter-quartile range (IQR) of the M-score, a measurement of inter-sample heterogeneity, derived from comparing the differences in signal intensity of given probesets across different samples of the same normal or tumor cell type. The IQR reflects the extent of inter-sample methylation variation by measuring the spread of the distribution of M-scores (Figure 1C).

Using these indicators for normal cell types, we observed a strong bimodal distribution of probeset intensities, indicating that the vast majority of gene promoter CpGs were predominantly either unmethylated or methylated within the cells of a sample, as represented by the two modes at positive or negative M-scores, respectively (Figure 1B). This observation is consistent with previous studies noting the bimodality of DNA methylation distributions in normal tissues [7], [9]. In contrast, the distribution of DNA methylation in lymphoma samples was significantly different from those of normal cells (Figure 1B; Kolmogorov-Smirnov test between pairs of normal and lymphoma samples; FDR corrected p-value<2.2×10^−16^). All lymphoma subtypes showed a significantly greater proportion of probes with an intermediate M-score, indicating increased intra-sample variation, and most notably, such variation increased progressively from FL to GCB to ABC DLBCLs. This intra-sample variation was not due to sample purity, which was high for both lymphoma and normal (NBC, NGC; >90% purity) samples as confirmed by flow cytometry, and was not accounted for by differences in cellularity among the samples [10] (Text S1, Module 1). In order to prove that intra-sample variation is an inherent feature of neoplastic transformation, rather than a technical artifact or a result of a confounding biological factors, we performed analysis controlling for (i) copy number variations using SNP data (Figure S1), (ii) sample purity using % purity data (Figure S3), (iii) exclusion of low signal-to-noise ratio probes from the analysis (Figure S4), (iv) differences in mitotic rate using cell line data with known doubling times (Figure S11; Table S2), and (v) potential age differences between controls and DLBCL patients (Figure S12). Finally, we validated the observation of increasing intra-sample heterogeneity in DLBCLs using the MassARRAY and ERRBS orthogonal assays, which supported our findings (Text S1, Module 1, and Figures S5, S6, S7).

Likewise, we then found that the IQR values, which represent inter-sample variation, were small in normal B-cell controls, but again progressively increased in FL and the GCB and ABC subtypes of DLBCL (Figure 1C; Mann-Whitney test between pairs of normal and lymphoma tissues; FDR corrected p-value<2.2×10^−16^). We also obtained consistent results using alternative approaches to profile methylation changes as well as an alternative definition of inter-sample variation (Text S1 Module 1, Figure S10). Since higher-grade lymphomas are known to display genomic instability, we verified that the observed differences in methylation in lymphomas were not due to gain or loss of genomic material by controlling for copy number alterations using SNP data from the same patients (Text S1, Module 1, and Figure S1). Variability was also independent of whether the probes were localized in CpG islands or not (Text S1, Module 2; Figure S13). We found that the promoter regions with high CpG density usually were more hypo-methylated than others, as observed using both HELP and ERRBS assays, but that the CpG density did not affect patterns of inter-sample variation (Text S1, Module 2; Figures S14, S15 S16). Notably, the probes with high intra-sample variation (i.e., M-scores near zero) were also likely to have high inter-sample variation (i.e., high IQR) in normal and lymphoma samples (Figure 1D); this finding is consistent with the identification of variable CpGs in solid tumors [11].

In summary, since FLs are diagnosed most often as indolent tumors while GCB and ABC DLBCLs have progressively worse prognosis, our findings suggest that the extent of intra-and inter-sample variation in DNA methylation increases with disease aggressiveness. Based on our cell line data (Text S1, Module 1, and Figure S11) it is unlikely that the greater epigenetic heterogeneity in more aggressive tumors is a reflection of higher proliferative rates that lead to stochastic variation in the DNA methylation distribution. Alternatively, heterogeneity could be related to loss of function of specific epigenetic regulators that normally tightly control DNA methylation patterns. Either way, epigenetic diversity could foster the survival of subpopulations of lymphoma cells after exposure to cytotoxic drugs, thus contributing to the greater risk of relapse in ABC DLBCLs. We found that DNA methylation diversity initiates within NGC, which are more heterogeneous than NBCs (Figure 1B–1D), which is consistent with recent findings [10]. All three lymphoma subtypes originate via different molecular and likely epigenetic mechanisms from a common precursor – germinal center B-cells. Each subtype is characterized by a different extent of epigenetic heterogeneity, which likely reflects different mechanisms of lymphomagenesis. Epigenetic diversity might then cooperate with somatic mutations in predisposing NGC towards malignant transformation.

### The patterns of aberrant DNA methylation predict patient survival

**Figure 2:**
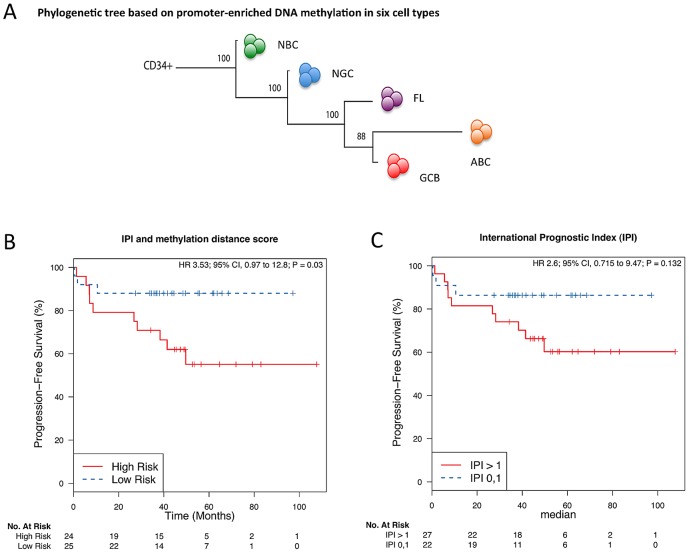
The extent of DNA methylation aberration is predictive of patient survival. (A) Phylogenetic tree, as estimated based on the correlation of group-averaged M-scores. Departure from normal methylation patterns is correlated with disease severity of the lymphoma samples. (B–C) Kaplan-Meier curves for risk groups defined according to their methylation distance score (i.e. distance from normal B-cells), which reflects how different a sample's methylation profile is from that of NBC or NGC, for all DLBCL (GCB and ABC) samples. (B) Multivariate analysis with the International Prognostic Index (IPI) and distance to NBC. (C) Only IPI.

It is not known whether alterations in DNA methylation patterning are associated with clinical outcome in lymphomas. Using a phylogenetic clustering approach [12], which arranges samples according to their distance in methylation patterning from that of undifferentiated cells, we found that genome-wide DNA methylation undergoes progressive changes from bone marrow CD34+ hematopoietic progenitor cells to NBC and NGC, FL and then DLBCL (Figure 2A). This finding reflects the ontogeny of normal B-cell development, the origin of B-cell lymphomas in NGC, and the increased aggressiveness of DLBCL subtypes. We then performed a Kaplan-Meier analysis using a methylation heterogeneity score derived from the distances of the methylation pattern of each tumor to that of the methylation pattern of NGC (Text S1, Modules 1 and 3; Figures S18, S19; Tables S3, S4). Cox models incorporating the International Prognostic Index (IPI) [13] and methylation heterogeneity score as covariates were then utilized for stratification of patients into high- and low-risk groups, depending on whether their estimated risk scores were above or below the cohort median. Analyzing the GCB and ABC samples together, we found that the methylation heterogeneity score improved the concordance [14] of the predictions of the IPI from 0.64 to 0.7 (ΔC 0.06; 95% CI −0.08–0.20; Text S1, Module 3) and yielded a significant risk stratification (HR = 3.85, p<0.03; Figure 2B). Thus, we found that the extent of aberrant methylation, as measured by the distance of a patient sample in terms of its methylation patterning from that of normal B-cells, is a significant predictor for survival: disease types with high intra-sample methylation variation have a poor prognosis and short survival while disease types with low intra-sample methylation variation have a good prognosis and long survival. Increased epigenetic heterogeneity may reflect the presence of diverse tumor cell populations in the patient, which in turn increases the risk of resistance and of the emergence of more aggressive clones, thus leading to poor prognosis. In a complementary analysis, we grouped the FL samples according to grade and found that DNA methylation patterning becomes increasingly heterogeneous with an increase in disease severity in FL (Text S1, Module 3; Figure S20). Taken together, our results demonstrate that the landscape of epigenetic DNA modifications is associated with the degree of neoplastic transformation and aggressiveness of a tumor.

### Aberrant cytosine methylation patterning is dependent on chromosomal regions and local gene density

**Figure 3:**
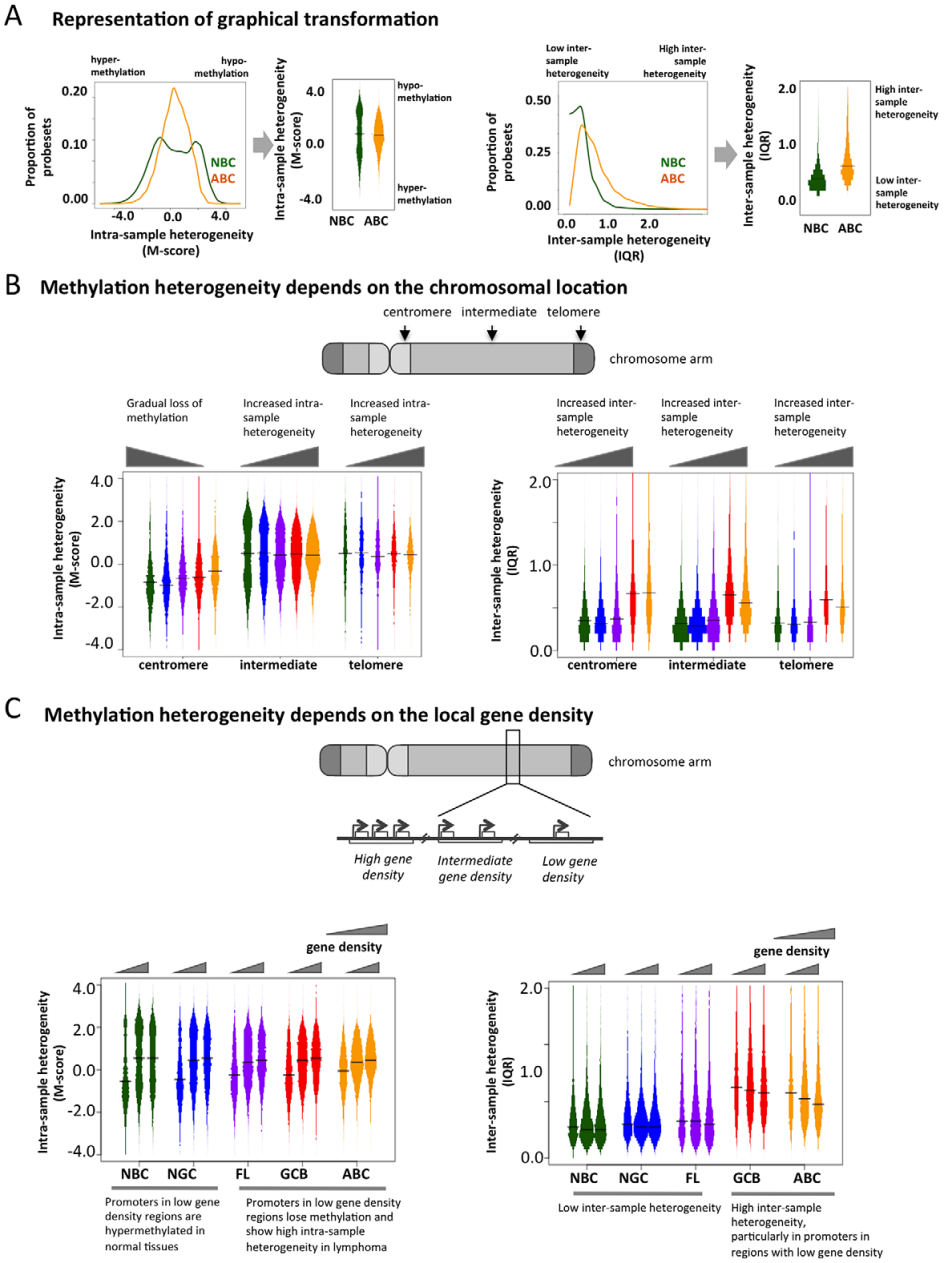
Genome-wide patterns of aberrant methylation. (A) Graphical explanation of how the distribution of M-scores and IQR are transformed into violin distribution plots to enable more efficient visualization and comparison on intra- and inter-sample variability. (B) Distribution of the methylation score (M-score, left) and inter-quartile ranges (IQR, right) at probesets in centromeric, telomeric, and intermediate regions for normal and diseased tissues. Bar width is proportional to the number of data points, and the colors are the same as in Figure 1A. (C) Distributions of M-score (left) and IQR (right) are shown for gene-poor, gene-rich, and intermediate regions.

To determine whether genomic features direct the aberrant cytosine methylation distribution in lymphomas, we examined DNA methylation diversity at the chromosomal regional level. In order to facilitate the visualization of intra-sample (M-scores) and inter-sample (IQR) heterogeneity in DNA methylation, we transformed the histograms shown in Figure 1B and 1C into a “violin” plot format (Figure 3A). Chromosomes were separated into telomeric, centromeric, and intermediate regions. We observed that centromeric regions were hyper-methylated in normal cells but exhibited a gradual loss of methylation in lymphomas (Figure 3B). Intermediate chromosomal regions displayed increasing intra-sample variation with disease severity, i.e. NBC<NGC<FL<GCB<ABC (p-value for NBC-FL, NBC-GCB and NBC-ABC pairs<2.2×10^−16^; Kolmogorov-Smirnov test), suggesting that much of the heterogeneity observed in the initial analysis is localized in these regions. All three regions displayed an overall tendency towards greater inter-sample variation in lymphoma cells compared to normal cells throughout all three chromosomal regions. These results were also validated using the ERRBS assay (Text S1, Module 2; Figure S17).

To investigate whether disruption of cytosine methylation is associated with gene density, we divided the genome into non-overlapping windows classified as gene-rich, intermediate, or gene-poor (Methods section). We found that in normal B-cells, gene-rich regions displayed a bimodal methylation pattern, while gene-poor regions were mostly hyper-methylated. This distribution was perturbed in lymphomas, which exhibited increased intra-sample variation in gene-rich regions, while gene-poor regions displayed progressive hypo-methylation compared to normal B-cells (Figure 3C). Inter-sample variation was low in normal cells in both gene-poor and gene-dense regions, but significantly increased in the lymphoma subtypes for both categories (FL: p-value<1×10^−3^, GCB and ABC: p-value<1×10^−10^; Mann Whitney test). Our findings were robust even after excluding centromeric and telomeric regions (Text S1, Module 4; Figure S21). Taken together, our results show that abnormal methylation patterns in lymphoma samples depend on chromosomal regions and local gene density. This differential aberration in gene-poor versus gene-rich areas suggests that these changes are not random, but are directed by genomic or epigenomic modifiers.

### Aberrant DNA methylation patterning spreads locally between genes but is limited by CTCF

**Figure 4:**
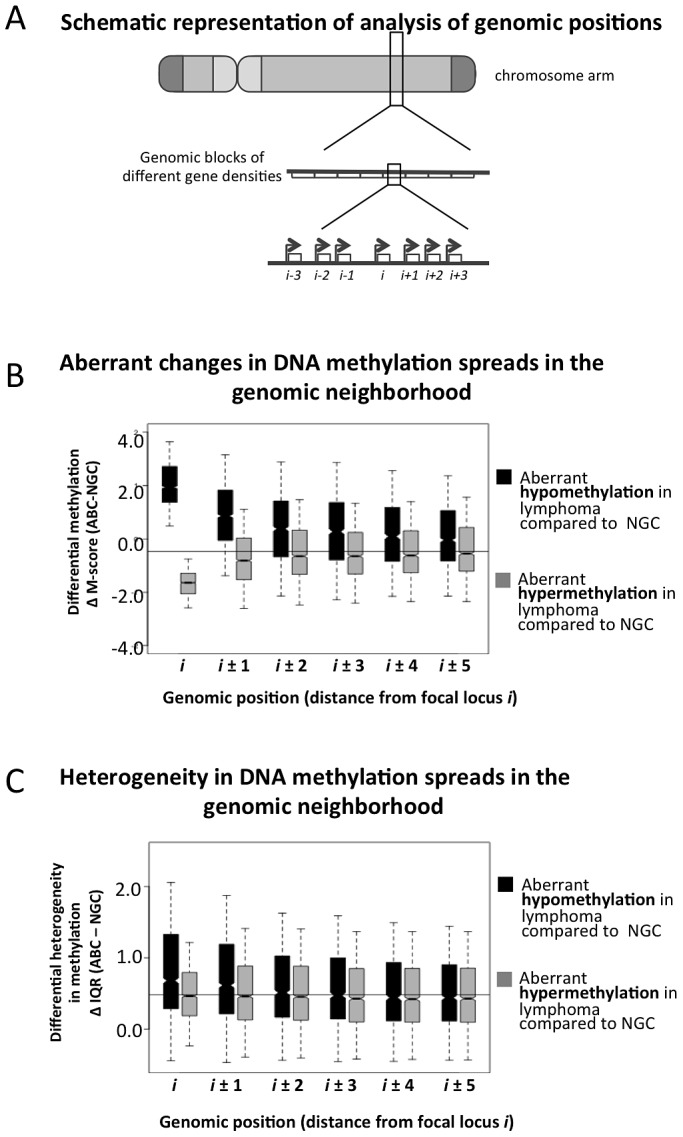
Spreading of aberrant methylation to neighboring probesets in the ABC samples. (A) A schematic representation of how the genome was divided into blocks of genes to study spreading of altered DNA methylation. (B–C) Analysis of spreading of aberrant methylation within genomic neighborhoods. Loci “*i*” represent probesets that are significantly hypo- (black) or hyper-methylated (grey) in lymphoma samples compared to normal tissues, and loci “*i*±*j*” represent both the (*i*+*j*)-th and (*i*−*j*)-th neighbors of those probesets. For instance, when we focused on probeset #10 (i.e. *i* = 10), we analyzed spreading of aberrant methylation at probesets #5, 6, 7, 8, 9, 11, 12, 13, 14 and 15. Panel B displays the change in methylation states while panel C shows the change in IQR (variability between samples).

We next focused at the level of specific genes and their impact on DNA methylation of neighboring genes (Figure 4A). We found that 3,414 and 2,044 probesets were significantly hyper- and hypo-methylated in ABC vs. NGC specimens, respectively (FDR-corrected p-value<5.0×10^−3^, Text S1, Module 5). For each of these hypo- and hyper-methylated promoters (denoted as “*i*” in Figure 4A and 4B), we investigated their neighboring promoter probesets (“*i*+1”, “*i*−1”, “*i*+2”, “*i*−2” up to “*i*−5” and “*i*+5”). For both hyper- and hypo-methylated promoter probesets, we found that their neighboring promoter probesets also displayed a change in methylation in the same direction (Figure 4B), and that this effect weakened with increasing distance, i.e. decayed from *i*+1 (or *i*−1) to *i*+5 (or *i*−5). Therefore, when a promoter displayed aberrant hypo- or hyper-methylation in lymphoma samples, their neighboring promoters were also likely to follow a similar trend. For instance, when the *i*-th promoter probeset was aberrantly hypo-methylated (ΔM-score>0), then the *i*±1 (i.e. *i*+1 and *i*−1) promoter probesets were also significantly aberrantly hypo-methylated (ΔM-score>0; p-value: 4.56×10^−5^); and when the *i*-th promoter probeset was aberrantly hyper-methylated (ΔM-score<0), then the *i*±1 positions were also significantly aberrantly hyper-methylated (ΔM-score>0; p-value: 3.11×10^−3^). This effect was stronger for hypo-methylated loci. For instance, when the *i*-th probeset was aberrantly hypo-methylated (ΔM-score>0), then the *s*±5 (i.e. *i*+5 and *i*−5) positions were also significantly aberrantly hypo-methylated (ΔM-score>0; p-value: 3.01×10^−3^), but the effect was not significant in the case of aberrant hyper-methylation (ΔM-score<0; p-value>0.05 at *i*±5 positions). We then found that the aberrantly hypo-methylated promoters, but not the hyper-methylated ones, generally displayed a greater extent of inter-sample variation among ABC lymphomas (Figure 4C). Our results were similar for the other lymphoma subtypes (Text S1 Module 5; Figures S22 and S23), and at par with published reports that local DNA methylation and histone modification (H3K9me3) patterns spreads to neighboring regions. [15]–[17] Thus it is likely that abnormal promoter methylation, especially hypo-methylation, tends to spread to neighboring promoters along the chromosomes; however, at this stage we cannot rule out other possibilities.

**Figure 5:**
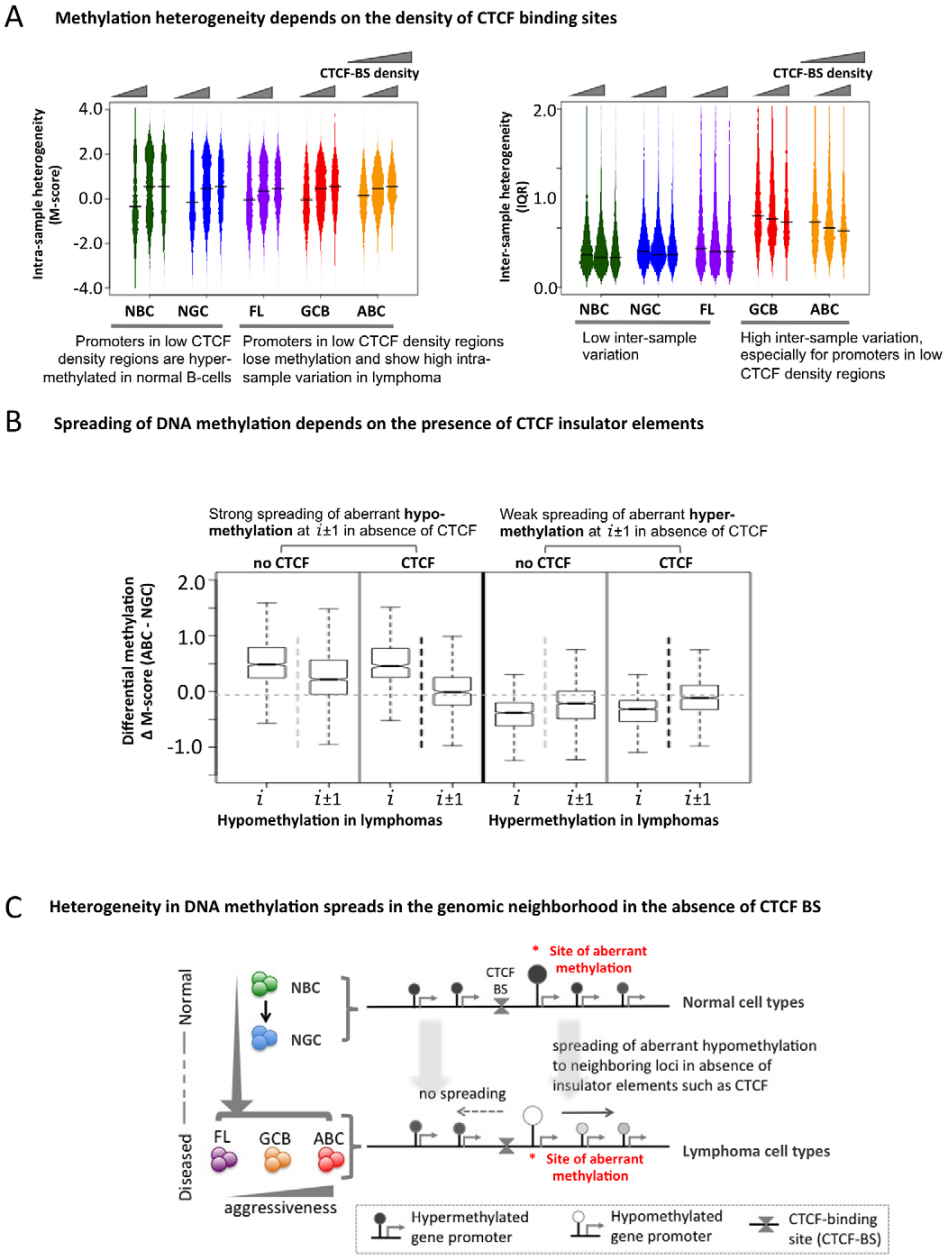
The insulator factor CTCF prevents spreading of aberrant methylation. (A) Methylation heterogeneity depends on the density of CTCF-binding sites. Methylation state (M-score, left) and inter-sample methylation variation (IQR, right) are shown for CTCF-BS-poor, CTCF-BS-rich, and intermediate regions. (B) Spreading of aberrant methylation from genomic position “*i*” to “*i*±1” (i.e. two neighboring sites) when at least one CTCF-BS is present (black vertical dotted line) and when no CTCF-BS is present (light grey vertical dotted line) between “*i*” and “*i*±1”, for aberrant hypo-methylation (two left panels) and aberrant hyper-methylation (two right panels). The presence of CTCF-BS more efficiently restricts the spreading of aberrant hypo-methylation. (C) A schematic overview showing spreading of abnormal methylation in the absence of CTCF-binding sites in genomic neighborhood.

The transcriptional repressor CTCF contributes to the organization of chromatin domains and the spatial delimitation of epigenetic marks [18]. Hence, we investigated whether CTCF was associated with the DNA methylation status of genes in normal and lymphoma cells. Overlaying published genome-wide CTCF ChIP-seq data [18], [19], we found that promoters in CTCF-binding site (BS)-poor regions were usually hyper-methylated in normal B-cells, but hypo-methylated in lymphomas (FL, GCB and ABC) (Figure 5A–5B). There was little inter-sample variation in normal cells regardless of the density of CTCF binding, whereas in the lymphoma subtypes, CTCF-BS-poor regions displayed significantly greater inter-sample variation than CTCF-BS-dense regions (p-value for NBC: 1.040×10^−6^; NGC: 6.656×10^−7^; FL: 2.9×10^−13^; GCB: 1.367×10^−11^; ABC: <2.2×10^−16^, Mann Whitney test). Our findings were robust even after excluding centromeric and telomeric regions (Text S1, Module 6; Figure S24). These data suggest that CTCF-BS-poor regions are more susceptible to epigenetic deregulation.

Since CTCF can establish boundaries between genomic regions, we tested whether it might affect DNA methylation spreading between loci and whether this function was perturbed in lymphomas. We divided the probesets into two groups: those in which neighboring promoter probesets were separated by at least one CTCF-BS, and those in which neighboring promoter probesets were not separated by any CTCF-BS. First, focusing on the promoters hypo-methylated in ABC versus NGC, we found that promoter pairs not containing intervening CTCF–BS displayed greater spreading of aberrant hypo-methylation from one promoter (probe set *i*) to the neighboring promoters (probe sets *i*+1 and *i*−1) compared to those that had one or more intervening CTCF-BS (Figure 5B, comparing probe sets *i*+1, *i*−1 between the two groups, p-value = 2.2×10^−8^, Mann Whitney test). In contrast, we did not observe any impact of CTCF on genes with hyper-methylation in DLBCL (Figure 5B, p-value>0.05 at probesets *i*±1, Mann Whitney test). We obtained similar results for the FL and GCB samples and using the ERRBS assay instead of the HELP assay data (Text S1, Module 6; Figures S25, S26). Thus, CTCF is suspected to play a gatekeeper role in the spreading of aberrant hypo-methylation among genes in DLBCL (Figure 5C), although more work is necessary to rule out other possibilities.

### Lymphomagenic regulatory proteins contribute to aberrant DNA methylation patterning in DLBCL

**Figure 6:**
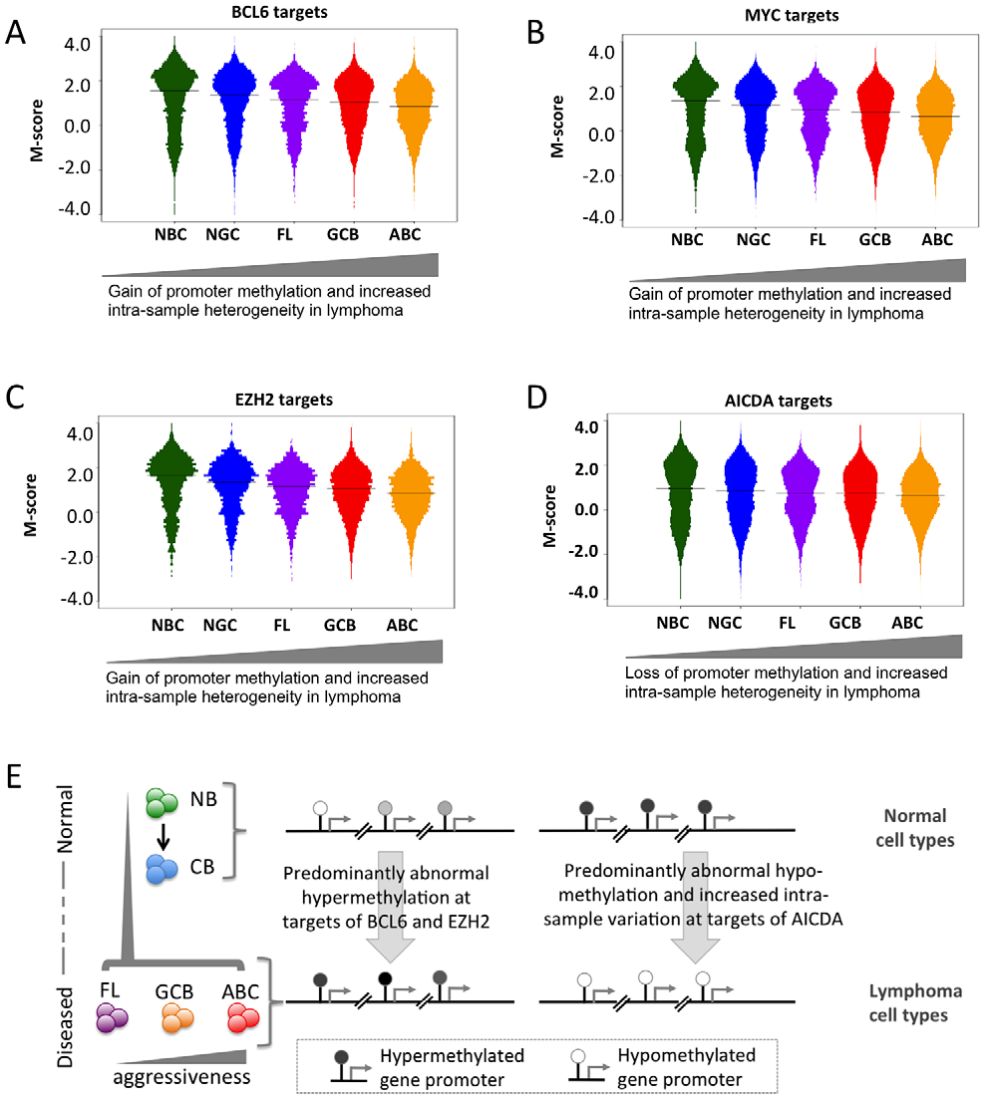
Genomic localization of transcriptional regulators and AICDA associates with sites of aberrant DNA methylation. (A–D) Methylation heterogeneity of promoters of genes that are targets of master regulators. The panels display the distribution of methylation scores (M-scores) for promoters of target genes of (A) BCL6, (B) MYC, (C) EZH2, and (D) AICDA. (E) A schematic overview showing targeted abnormal promoter methylation by master regulators such as MYC, BCL6, EZH2 and AICDA in the lymphoma subtypes.

We then investigated potential factors associated with the abnormal DNA methylome in lymphomas. We first investigated whether in general, there was an association between promoter methylation status and the expression of the same gene and found a positive correlation (Text S1, Module 7), which suggests that genes with a loss of promoter methylation were likely to experience increased expression. We then obtained genomic localization data, detected by genome-wide CHIP-chip or CHIP-seq studies, of four master regulators of lymphoid differentiation and lymphomagenesis: BCL6 [20], EZH2 [21], MYC (newly reported herein), and AICDA [22], and overlaid promoter methylation information (Text S1, Module 7; Figures S27, S28, S29). BCL6 is a transcriptional repressor that is expressed in NGCs and also in most DLBCLs and FLs [23], and its constitutive expression is known to drive malignant transformation of NGCs [24]; EZH2 is a Polycomb repressor protein also expressed in NGC [25] that is highly expressed in most DLBCLs [26] and is sometimes targeted by gain of function mutations [27]; and the MYC oncogene, is aberrantly expressed in DLBCLs, often through chromosomal translocations [20], [28]. AICDA is a cytosine deaminase that mediates single- and double-strand DNA breaks during somatic hypermutation and class switch recombination [29]. We first investigated the extent of change in the DNA methylation status of the BCL6 and MYC loci, including the surrounding genes, in lymphoma samples compared to that in the NBC samples, and found that both BCL6 and MYC loci experienced loss of promoter methylation in lymphoma samples compared to normal samples (Text S1, Module 7; Figure S29). Furthermore, we found that the target gene promoters of MYC, BCL6 and EZH2 were hypo-methylated in normal B-cells and became increasingly hyper-methylated in lymphoma samples (Figure 6A–6C; p-value<1×10^−4^ for all three cases; Mann-Whitney test). Gain of methylation at the promoters of the target genes of MYC, BCL6, and EZH2 in lymphomas was significantly higher than that at the promoters of other genes (Mann Whitney test, p-value<1×10^−5^ in each case). Since BCL6 and EZH2 are transcriptional repressors, accumulation of DNA methylation may reflect their constitutive activity at their targets in lymphoma cells. Notably, a previous report showed that EZH2 and H3K27 are mostly mutually exclusive with DNA methylation in NGC B-cells, but that this opposing relation is disrupted in DLBCL [21]. The reason for MYC targets acquiring hypermethylation is not as clear, but it is notable that the MYC and BCL6 ChIP-on chip binding patterns are highly overlapping (data not shown). In contrast, target genes of AICDA, such as *BRCA2*, *GATA1* and *LMO1* [22], displayed a loss of bimodality (Figure 6D). However, hypomethylation of promoters of AICDA target genes was not immediate apparent, perhaps indicating disruption or variability in AICDA binding to the genome in malignant cells. Nevertheless, AICDA expression was associated with a loss of DNA methylation at a genome-wide scale, as discussed in the following section in detail, which is consistent with the role of AICDA in demethylation [6], [30], [31]. AICDA plays a role in gene demethylation downstream of TET family protein-mediated hydroxylation of methylcytosine [30]. Moreover, we recently reported that genes that are hypo-methylated in NGC B-cells tend to be known direct targets of AICDA [10]. Collectively, aberrant DNA methylation in lymphomas is related in part to the action of constitutively expressed lymphomagenic regulatory factors during lymphomagenesis (Figure 6E).

**Figure 7:**
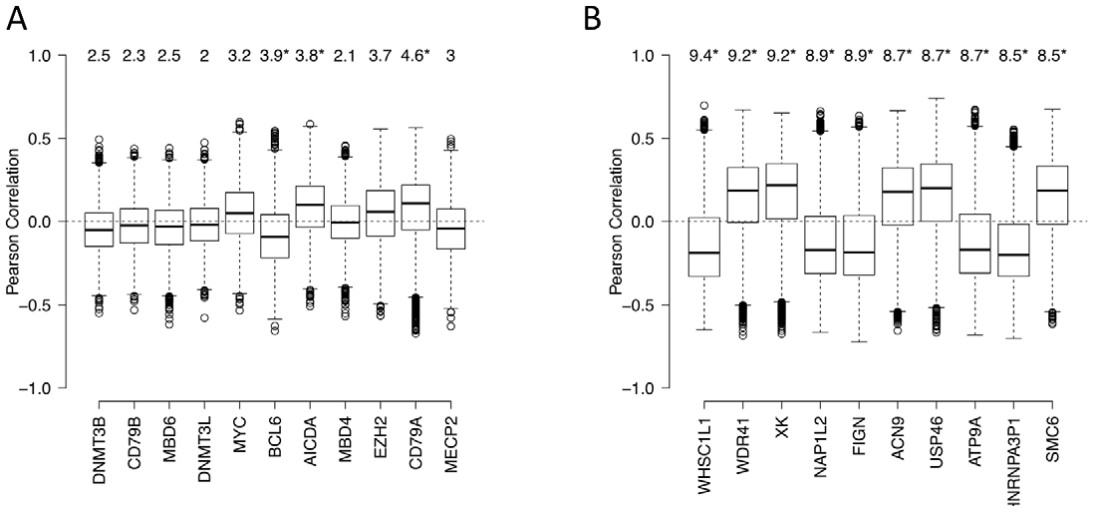
Genes associated with aberrant DNA methylation patterns B-cell lymphoma. (A) List of genes potentially associated with aberrant methylation patterns in DLBCL. Boxplots visualize the distribution of Pearson correlation coefficients of primary variable (expression level of a candidate gene) and the fitted variables (ΔM of promoters). The numbers on top represent the summarized quantity R^2^, i.e. statistical variance in the fitted variable explained by the primary variable (in percent). See Text S1, Module 7 for more details. Statistically significant R^2^ values (p<0.05) are marked with an asterisk. (B) List of the top 10 genes with highest R^2^ the unbiased genome-wide analysis. See Text S1, Module 7 for more details.

We next used an independent approach, integrating DNA methylation and gene expression profiling data in a subset of our cases, to identify factors driving or associated with the aberrant lymphoma methylome. First, we focused on a set of genes: DNMT3A, DNMT3B, DNMT3L, MYC, BCL6, AICDA, MBD4, MBD6, CD79A, CD79B and MECP2 – which include DNA methyltransferases, methyl-CpG binding domain proteins, as well as signaling and transcription factors involved in lymphoid differentiation and lymphomagenesis. We investigated whether the expression levels of these genes correlated with genome-wide aberrant DNA methylation patterns in DLBCL samples (see Text S1, Module 7 for details of the method). We found the following trends (Figure 7A): (i) the BCL6 expression level was significantly correlated with aberrant hyper-methylation at a genome-wide scale (p-value<0.05), which is consistent with a transcriptional repressor role of this gene, and (ii) expression levels of AICDA and CD79A were significantly correlated with aberrant hypo-methylation at a genome-wide scale (p-value<0.05 in both cases). This finding was significant given the role of AICDA in demethylation, as noted above [6], [30], [31]. The association was not significant for other genes in the list for our dataset. Larger patient cohorts will be necessary to test those cases systematically.

Some the above factors, such as DNMT3B, are associated with the maintenance of methylation in simple repeat sequences [32]. Indeed, overlaying DNA repeat sequence information, we found that both low-complexity repeats and simple repeats exhibited hypo-methylation in normal cells, but displayed an increasing extent of hyper-methylation in lymphoma samples (Text S1, Module 8; Figure S30). Note that the effect size of expression levels of each of the 11 genes, including DNA methyltransferases, is relatively modest; this may be at least partly due to the fact that these modifier genes influence the epigenetic state of their target genes by the recruitment of other enzymes or cofactors, and that the lymphoma samples show a high level of intra-sample variation. A completely unbiased genome-wide analysis exploring whether other genes on the expression array showed significant associations with the aberrant methylation pattern in lymphomas yielded a list of candidates provided in Figure 7B (see Text S1, Module 7 for details). Interestingly, some of the top genes are known epigenetic modifiers. For instance, the top candidate of the list, WHSC1L1, is a known histone methyltransferase and plays a key role in chromatin integrity [33]–[35]. Other top hits are important for the genomic and epigenomic integrity of the cell, such as NAP1L2, which promotes histone acetylation [36], and SMC6, which regulates chromosomal stability [37], [38]. Many of these genes are known epigenetic modifiers, downstream targets or co-factors of the shortlisted genes described in the paragraph above, while others may be novel factors associated with perturbation of DNA methylation patterning in DLBCLs. Systematic characterization of these candidates will be pursued further in forthcoming work.

### Conclusions

Through integrative analysis of DNA methylation, copy number variation, genomic sequence, gene expression and genomic localization data, our study provides insights into the architecture and biology of aberrant DNA methylation patterning in a human malignancy. Based on these analyses, we report three key findings: (a) DNA methylation exhibits considerable heterogeneity, both within individual lymphoma samples and between patients, and the degree of heterogeneity and departure from the DNA methylation pattern of normal B-cells correlates with disease severity and patient survival, (b) these abnormal methylation patterns are not randomly distributed but instead associate with chromosomal regions, local gene density, and the methylation status of neighboring genes, and (c) the pattern of DNA methylation abnormalities is at par with the effects of at least two distinct processes: i) lymphomagenic transcriptional regulators, such as BCL6 and EZH2, perturb DNA methylation in a target gene-specific manner; and ii) aberrant methylcytosine marks, especially promoter hypo-methylation, tend to spread to neighboring promoters in the absence of insulator elements such as CTCF. We propose that focal aberrant hyper- and hypo-methylation via target-specific recruitment of master regulators and non-specific spreading of aberrant methylation drives the generation of epigenetic abnormalities in follicular lymphoma and DLBCL. While our results themselves cannot pinpoint causality, they are consistent with emerging reports that highlight the roles of lymphomagenic transcriptional regulators [23], [24], [26] and that DNA methylation patterns tend to spread in a genomic neighborhood [15]–[17]. Recently, Lai et al. showed that BCL6 expression is maintained during lymphomagenesis in part through DNA methylation that prevents CTCF-mediated silencing [39], and our results confirm their conclusions. The fact that epigenetic diversity is first observed in NGC B-cells further suggests that epigenetic heterogeneity may originate in these rapidly dividing cells and potentially contributes to malignant transformation. Since epigenetic abnormalities increase with disease aggressiveness and are a predictor of patient survival, clonal epigenetic diversity and evolution may increase the survival advantage of lymphoma cells, leading to more aggressive and chemo-resistant tumors. Heterogeneity in DLBCL DNA methylation patterning does not preclude the co-existence of subtype-specific DNA methylation profiles, in which specific genes are differentially methylated. Indeed, we previously observed that ABC and GCB DLBCLs feature distinct and specific DNA methylation signatures, involving genes of potential functional significance, most notably including hypermethylation of a TNFα gene network in ABC DLBCLs [6]. These signatures represent a core of stably affected loci within the larger context of more variable DNA methylation disruption as reported herein. While previous studies including ours aimed at identifying key genes and pathways dysregulated in lymphomas, here we add another dimension to these studies by highlighting the implications of epigenetic heterogeneity at a genome-wide scale, and also the complex interaction between master regulators and insulator elements that contribute to establishing an abnormal methylome during lymphomagenesis. Our approach can be used to analyze other tumor types and delineate the contribution of aberrant methylation patterning to the development of human cancers.

## Methods

### Sample collection

Samples used in the study included naïve B-cells (NBC; 8 samples), normal germinal center B-cells (NGC; 10 samples), follicular lymphoma (FL: grades 1 and 2 representing lower grade lymphoma than Diffuse Large B-cell Lymphoma (DLBCL); 8 samples), germinal center B-cell-like DLBCL (GCB: DLBCL with better prognosis; 39 samples), and activated B-cell-like DLBCL (ABC: DLBCL with worse prognosis; 18 samples). All FL and DLBCL samples used in the study were selected based on their high content of neoplastic cells from primary diagnostic material preceding treatment and were obtained by the Vancouver Cancer Center in British Columbia, Canada. The FL and DLBCL samples represent soft tissue biopsy material. The percent of neoplastic cells in the biopsy was determined based on pathologic evaluation using morphologic criteria and immunohistochemical characteristics of the neoplastic cells (expression of CD79B, CD20, BCL2, CD10, CD43, BCL6 antigens).The use of human tissue was in agreement with IRB of the Vancouver Cancer Center and Weill Cornell Medical Center. Primary NBC and NGC B-cells were purified from reactive human tonsillar specimens. Tonsils were minced on ice and mononuclear cells were isolated using Histopaque density centrifugation. All washes were performed in PBS/2% Bovine Serum Albumine/2% EDTA. All antibodies were used at 1∶100 dilution in cold PBS and staining was done for 10 min on ice, followed by 3 washes. The B-cell populations were separated using the AutoMACS system (Milteny Biotec, Auburn, CA) using the “posselD” program. In brief, NBC cells were separated using depletion of GC cells, T-cells, plasma and memory cells (CD10, CD3, CD27), followed by enrichment for IgD+ B-cells; GCB cells were separated by positive selection with CD77 (anti-CD10: BD Biosciences cat# 555373 Lot 59624, anti-CD3: BD Biosciences cat# 555332 Lot 59347, anti-CD27: BD Biosciences cat# 555439 Lot 71274, anti-CD77: Serotec cat# MCA579 Batch 180510, anti-IgD: BD Biosciences cat# 555778 Lot 58641). While the tissue environment of the collected normal and lymphoma cells (e.g. cytokine exposure level) differ, this is unlikely to bias our analyses. All NBC and NGC samples yielded a purity of >90%. For patient characteristics (Table S1) see Shaknovich et al. [6] and GEO number GSE23967.

### HELP assays and analysis of DNA methylation data

We assayed genome-wide patterns of promoter methylation using HELP assays and custom-designed oligonucleotide arrays. HELP assays were performed based on the standard protocol [7]. One µg of high molecular weight genomic DNA was digested with HpaII and MspI (NEB, Ipswich, MA), digestion products were extracted with phenol-chloroform and resuspended in 10 mM TRIS-HCl pH8, after which they were subjected to ligation of HpaII adapter using T4 DNA Ligase. This approach was followed by PCR amplification and labeling of HpaII and MspI digestion products and co-hybridization to custom NimbleGen HELP microarrays (NimbleGen, Inc. Madison, WI). The microarray design was previously published and represents >50,000 CpGs corresponding to 14,000 promoters [6], [7].

Data processing was performed using the published HELP pipeline [40]. Intra- and inter-array normalization was performed by subtracting mean random probe intensity separately within HpaII and MspI channels, after which quantile normalization was performed within each channel independently. Quantile normalized log2(HpaII/MspI) values, denoted as M-scores, were subsequently used for all further analysis. The probes whose intensity of Msp1 channel was less than 2.5 mean absolute standard deviations from the mean of log2(Hpa2/Msp1) of random probes were considered failed and removed from the analysis. Since the Msp1 channel served as an internal control, it allowed us to remove the probes that had low intensities due to genomic deletions, thus avoiding false positives for hypermethylation. We also calculated the inter-quartile range (IQR) of the M-scores between the samples within the same normal or disease group at a given locus to reflect inter-sample methylation diversity. The analysis was based on the Human Reference Genome version hg19 [41] and the list of human protein-coding genes was obtained from Ensembl v59 [42]. HELP arrays for DLBCLs can be found in GEO number GSE23967 and for CD34+ cells in GEO number GSE18700. NBCs, NGCs and FLs data is pending GEO accession number.

### Phylogenetic tree analysis

HELP methylation data for CD34+ hematopoietic progenitor cells was obtained from the NCBI Geo database (GSE18700). HELP was performed in the same manner as the normal (NBC and NGC) and lymphoma (FL, GCB and ABC) samples, hybridized on the same methylation microarray and normalized using the same protocol together with the normal and lymphoma samples. Only promoter methylation probes without missing values were considered for further analysis. M-scores were averaged for each group (CD34+, NBC, NGC, FL, GCB and ABC) on each probe. Pairwise distances between groups were then calculated with the Pearson correlation distance on the group-averaged M-scores. This approach was repeated 1000 times, with bootstrapping of promoter methylation probesets and samples. Then, phylogenetic trees were constructed using the FastME method, implemented in the ape R package, on these 1000 distance matrices. A consensus tree was calculated in Dendroscope. The code is available as R package at https://github.com/lima1/maphylogeny.

### Gene expression analysis

We obtained both genome-wide promoter methylation and gene expression data for 4 NBC and 45 DLBCL samples (13 ABC and 32 GCB samples). Expression data for NBC were obtained from GSE15271, generated using Affymetrix HG133_Plus2_microarray and mas5 normalized together with the expression data for the DLBCL samples (GSE23501). The processing of RNA, hybridization, and image scanning were performed as per Affymetrix protocols. The trimmed mean target intensity of each array was set to 500. Expression-based classification labels GCB and ABC were assigned as published in Shaknovich et al. [6]. The gene expression data for all DLBCLs was deposited to GEO number GSE23501.

### ChIP-on-chip analysis

MYC ChIP-on-chip analysis was performed using Ramos cells. First, Ramos cells were fixed in 1% formaldehyde for 10 min, quenched with glycine and washed three times with PBS. Cells were then resuspended in lysis buffer and sonicated 6×30 sec (amplitude 55%) in an Ultrasonic Dismembrator Model 500 (Fisher) to shear the chromatin to an average length of 500 bp. Supernatants were precleared using protein-A agarose beads (Roche) and 10% input was collected. Immunoprecipitation was performed in 10^7^ cells using antibodies against MYC (Santa Cruz). DNA-protein complexes were pulled down using protein-A agarose beads and washed. DNA was recovered by overnight incubation at 65°C to reverse cross-links and purified using QIAquick PCR purification columns (Qiagen). ChIP products and their respective input genomic fragments were amplified by ligation-mediated PCR [43]. Q-ChIP was repeated after amplification to verify that the enrichment ratios were retained. The genomic products of three biological ChIP replicates were labeled with Cy5 (for ChIP products) and Cy3 (for input) and co-hybridized on a NimbleGen human promoter array representing 1.5 kb of promoter sequence from >24,000 genes (human genome version 35, May 2004) according to manufacturer's protocol (Roche NimbleGen, Inc., Madison, WI). The enrichment for each promoter was calculated by computing the log ratio between the probe intensities of the ChIP product and input chromatin, which were co-hybridized on the same array. Thereafter, for each of the >24,000 promoter regions, the maximum average log ratio of three neighboring probes in a sliding window was calculated and compared with random permutation of the log ratios of all probes across the entire array. The MYC ChIP-on-chip data is available on GEO (accession number GSE31110).

The Chip-chip data for BCL6 and EZH2 were previously published [20], [21]. AICDA ChIP-seq data was obtained from the recently published study in mouse activated B-cells [22] (GEO accession number GSE24178). Short reads were aligned to the mm9 genome and ChIP-seq peaks were called using the ChIPSeeqer program (http://icb.med.cornell.edu/wiki/index.php/Elementolab/ChIPseeqer_use). Peaks within RefSeq gene promoters, defined as 4 kb windows centered on transcription start sites, were then extracted. Human and mouse unambiguous orthologs were then determined using the reciprocal best BLAST strategy with protein sequences obtained from RefSeq (and matched with RefSeq transcripts). Human genes whose mouse orthologs were associated with 1 or more AICDA peaks in mouse activated B-cells were then determined.

We obtained CTCF binding site data from InsulatorDB (http://insulatordb.uthsc.edu; downloaded Jan, 2011), where CTCF binding sites (CTCF-BS) were determined using ChIP-on-chip and computational approaches [18], [19], [44]. We performed our analysis using experimentally determined CTCF-BS from this database, and obtained similar results using computationally predicted CTCF-BS from this database.

All statistical analyses were performed in R.

## Supporting Information

Figure S1 Copy number analyses. (A) Distributions of M-scores against copy number log2 ratios for two GCB and two ABC samples. (B) Boxplots showing the distributions of M-scores against copy number loss, gain, and wild type (wt) for those GCB and ABC samples.
(TIF)

Figure S2 Copy number analyses for frequently amplified or deleted regions. (A) The distributions of M-scores against copy number log2 ratios for two GCB and two ABC samples. DNA promoter methylation probes in the regions that were amplified or deleted in a given sample, and also overlapped with GISTIC peaks, are shown in black, and remaining probes are shown in grey. (B) Boxplots showing the distributions of M-scores against copy number loss, gain, and wild type for those GCB and ABC samples.
(TIF)

Figure S3 The frequency distribution of (left) the median M-score and (right) inter-quartile ranges (IQR) of the M-score, per methylation probe of gene promoters for normal and diseased samples with ≥80% purity. The color code is similar to that of Figure 1A in the main text.
(TIF)

Figure S4 Frequency distribution of M-scores at the genome-wide methylation probe positions. Data is shown after removal of low signal to noise ratio probes. Color codes are the same as in Figure 1.
(TIF)

Figure S5 Scatter plot showing the M-score as reported by the HELP assay and the percentage DNA methylation as reported by the RRBS assay for 6 samples. Pearson correlation coefficient (top right corner) and regression lines are shown for each panel.
(TIF)

Figure S6 MassARRAY validation shows that there is greater variance in methylation within DLBCL samples than in normal B-cell samples.
(TIF)

Figure S7 Log(variance ratio) vs. q-value plot demonstrates greater variance of CpG methylation values derived from MassARRAY validation. Only 4 CpGs had a lower variance in DLBCL but the remaining CpGs had a higher variance in DLBCL as compared to NGC samples. The dashed line on the left represents equal variance between the two groups. The dashed line on the right represents 3× higher variance in DLBCL as compared to NGC.
(TIF)

Figure S8 Technical validation of the HELP array using MassARRAY Epityping reveals a linear relationship between these two assays. These validation studies revealed that 1 unit of log2(HpaII/MspI) change in HELP intensity corresponds to 30% change in methylation as detected by the MassARRAY.
(TIF)

Figure S9 Distribution of the sites of hypo- and hyper-methylation along the human chromosomes in both normal and lymphoma samples. The color code is similar to that of Figure 1A in the main text.
(TIF)

Figure S10 Distribution of inter-sample standard deviation of M-score, grouped by NBC, NGC, FL, GCB and ABC. Outliers are not shown.
(TIF)

Figure S11 Distribution of M-score of the cell lines which are grouped into three categories – low, intermediate and high, based on their doubling times. In each panel, the X-axis represents the M-score, and the Y-axis represents the frequency of promoter methylation probe sets which have that M-score. The HELP ID of the cell lines and their doubling time are provided at the top of each panel. The last column represents the distribution of the M-score for the group average. Median and the two quantiles are highlighted in red.
(TIF)

Figure S12 Distribution of % methylation using the eRRBS assay at CpG sites in young and old B-cell controls and in DLBCLs.
(TIF)

Figure S13 Distributions of M-scores at the methylation probesets overlapping with CpG islands and non-CpG islands for normal B-cell and DLBCL samples. The color code is as following: NBC-green, NGC-blue, FL-purple, GCB-red and ABC-orange. For each sample type, bar width is proportional to the number of probes with a given M-score, as discussed in details in Figure 3A in the main text.
(TIF)

Figure S14 Distribution of M-score against CpG density at gene promoters for NBC, NGC, FL, GCB, and ABC samples. The regression line for each category is shown in black.
(TIF)

Figure S15 Distribution of the percentage DNA methylation as estimated for the number of CpG sites in the gene promoters for 6 DLBCL samples. The regression line for each category is shown in black.
(TIF)

Figure S16 Distribution of (i) |M-score| (y-axis, top) and (ii) IQR (y axis, bottom) against CpG density at gene promoters for NBC, NGC, FL, GCB, ABC samples. The regression line for each category is shown in black.
(TIF)

Figure S17 Distribution of % methylation using the eRRBS assay at CpG sites in centromeric, telomeric, and intermediate regions for normal and diseased tissues. In each panel, the vertical bar represents the median value of the respective distribution.
(TIF)

Figure S18 Kaplan-Meier comparison of the risk stratification by stage and methylation heterogeneity score (MHS; left) and stage alone (right) in ABC and GCB.
(TIF)

Figure S19 Kaplan-Meier analysis in ABC only. (A) MHS; (b) IPI; (c) stage; (d) IPI+MHS; (e) stage+MHS.
(TIF)

Figure S20 Distribution of M-score of the FL samples which are grouped according to their grades. In each panel, the X-axis represents the M-score and the Y-axis represents the frequency of promoter methylation probe sets, which have that M-score. The HELP ID of the cell lines and their grade are provided at the top of each panel.
(TIF)

Figure S21 Distributions of M-score for gene-poor, intermediate, and gene-rich regions for normal and lymphoma samples. Color codes are the same as in Figure 1. Bar width is proportional to the number of probes with a given M-score, as discussed in details in Figure 3A in the main text.
(TIF)

Figure S22 Spreading of aberrant methylation in neighboring positions. Position “i” represents probes that are significantly hypo- (black) or hyper-methylated (grey) in lymphoma samples compared to normal tissues, and “i±n” represents the n^th^ neighbors of those probes. The difference in M-score (A and B) and the difference in between-sample variation, estimated by IQR (C and D) between the lymphoma and normal samples at “i”, “i±1”, … “i±5” positions are shown.
(TIF)

Figure S23 Spreading of aberrant methylation in neighboring gene promoters after excluding probesets that map to multiple genes or genes marked by multiple probesets. Position “g” represents probes that are significantly hypo- (black) or hyper-methylated (grey) in lymphoma samples compared to normal tissues, and “g±n” represents the n-th neighboring promoter probeset. The difference in M-score (A) and the difference in between-sample variation, estimated by IQR (B) between the lymphoma and normal samples at “g”, “g±1”, … “g±5” positions are shown.
(TIF)

Figure S24 Distributions of M-score for regions with low CTCF-BS density, intermediate, and high CTCF-BS density in normal and lymphoma samples. Color codes are the same as in Figure 1. Bar width is proportional to the number of probes with a given M-score as discussed in detail in the main text.
(TIF)

Figure S25 Effects of CTCF-binding site on spreading of aberrant methylation in (left) FL and (right) GCB samples. Locus “*i*” refers to a promoter probe position that has significantly different methylation pattern in ABC compared to NGC, and “*i*±1” represent its immediate up- and downstream neighboring probe positions. Changes in M-score at “*i*” and “*i*±1” in the lymphoma samples relative to NGC samples were calculated for four different scenarios – depending on whether “*i*” had aberrant hypo- or hyper-methylation, and presence (black vertical dotted line) or absence (light grey vertical dotted line) of CTCF-BS between “*i*” and “*i*+1”. The horizontal line represents the genome-wide median Δ M-score between ABC and NGC samples. We found that the presence of CTCF-BS restricted the spreading of aberrant hypo- or hyper-methylation.
(TIF)

Figure S26 Effects of CTCF-binding sites (BS) on the spreading of aberrant methylation in DLBCL samples. Locus “*i*” refers to a promoter probe position that has significantly different methylation pattern in DLBCL compared to NGC, and “*i*±1” represent its immediate up- and downstream neighboring positions. Changes in % methylation at “*i*” and “*i*±1” in the lymphoma samples relative to NGC samples were calculated for four different scenarios – depending on whether “*i*” had aberrant hypo- or hyper-methylation, and presence (black vertical dotted line) or absence (light grey vertical dotted line) of CTCF-BS between “*i*” and “*i*+1”.
(TIF)

Figure S27 Inter-sample variation, measured using IQR, for the promoter methylation probe positions of the target genes of AICDA, BCL6, EZH2, and MYC and also all the promoter probe positions in our dataset.
(TIF)

Figure S28 Association of promoter methylation of the target genes of AICDA, BCL6, EZH2, and MYC and transcription factor gene expression. This association was tested utilizing gene set analysis (GSA) and the plots (A–C) visualize the GSA results. The bar plots on the bottom visualize the TF targets in a ranking by correlation with gene expression, while the plots on the top visualize the local enrichment, i.e., the deviation from a random ranking. A positive enrichment score indicates that targets have a higher (positive) correlation of promoter methylation with expression than expected by chance. Promoter methylation of both AICDA and MYC is significantly associated with expression of BCL6 (panels A and B, respectively), while EZH2 expression is anti-correlated with its target promoter methylation (C). The highly similar results in A and B are due to high overlap of targets, i.e., because many genes are regulated by both MYC and AICDA (D).
(TIF)

Figure S29 Extent of change in DNA methylation status of BCL6 (chr3:187 Mb) and MYC (chr8:128 Mb) loci, including the surrounding genes, in lymphoma samples (FL, GCB and ABC) compared to that in the normal NBC samples. Blue lines indicate CTCF binding sites.
(TIF)

Figure S30 Distributions of M-score and inter-sample variation (IQR) for methylation probes that overlap with common repeat elements in normal and lymphoma samples. M-scores are shown on the left and IQR on the right. Color codes are the same as in Figure 1. Bar width is proportional to the number of probes with a given M-score as discussed in Figure 3A in the main text. The dotted horizontal line separates low complexity and simple repeats from other repeat classes.
(TIF)

Table S1 Patient characteristics.
(PDF)

Table S2 The doubling times of the cell lines used.
(PDF)

Table S3 C-statistic with their standard errors (SE) and 95% confidence intervals of prognostic models in ABC and GCB samples. The C-statistic estimates the concordance of the predictions, which is the probability that in a random pair of non-censored patients, the one with higher risk relapsed earlier.
(PDF)

Table S4 C-statistics with their standard errors (SE) and 95% confidence intervals of prognostic models in ABC only.
(PDF)

Text S1 Supporting material.
(DOC)

## References

[1] TNHLCP (1997) A clinical evaluation of the International Lymphoma Study Group classification of non-Hodgkin's lymphoma. The Non-Hodgkin's Lymphoma Classification Project. Blood 89: 3909–3918. 9166827

[2] Tan D, Horning SJ (2008) Follicular lymphoma: clinical features and treatment. Hematol Oncol Clin North Am 22: 863–882, viii. 18954741 10.1016/j.hoc.2008.07.013

[3] Rosenwald A, Wright G, Chan WC, Connors JM, Campo E, et al (2002) The use of molecular profiling to predict survival after chemotherapy for diffuse large-B-cell lymphoma. N Engl J Med 346: 1937–1947. 12075054 10.1056/NEJMoa012914

[4] Klein U, Dalla-Favera R (2008) Germinal centres: role in B-cell physiology and malignancy. Nat Rev Immunol 8: 22–33. 18097447 10.1038/nri2217

[5] Shaknovich R, Cerchietti L, Tsikitas L, Kormaksson M, De S, et al (2011) DNA methyltransferase 1 and DNA methylation patterning contribute to germinal center B-cell differentiation. Blood 118: 3559–3569. 21828137 10.1182/blood-2011-06-357996 PMC3186332

[6] Shaknovich R, Geng H, Johnson NA, Tsikitas L, Cerchietti L, et al (2010) DNA methylation signatures define molecular subtypes of diffuse large B-cell lymphoma. Blood 116: e81–89. 20610814 10.1182/blood-2010-05-285320 PMC2993635

[7] Shaknovich R, Figueroa ME, Melnick A (2010) HELP (HpaII tiny fragment enrichment by ligation-mediated PCR) assay for DNA methylation profiling of primary normal and malignant B lymphocytes. Methods Mol Biol 632: 191–201. 20217579 10.1007/978-1-60761-663-4_12

[8] Figueroa ME, Skrabanek L, Li Y, Jiemjit A, Fandy TE, et al (2009) MDS and secondary AML display unique patterns and abundance of aberrant DNA methylation. Blood 114: 3448–3458. 19652201 10.1182/blood-2009-01-200519 PMC2765680

[9] Khulan B, Thompson RF, Ye K, Fazzari MJ, Suzuki M, et al (2006) Comparative isoschizomer profiling of cytosine methylation: the HELP assay. Genome Res 16: 1046–1055. 16809668 10.1101/gr.5273806 PMC1524864

[10] Shaknovich R, Cerchietti L, Tsikitas L, Kormaksson M, De S, et al (2011) DNA methyltransferase 1 and DNA methylation patterning contribute to germinal center B-cell differentiation. Blood 10.1182/blood-2011-06-357996 PMC3186332 21828137

[11] Hansen KD, Timp W, Bravo HC, Sabunciyan S, Langmead B, et al (2011) Increased methylation variation in epigenetic domains across cancer types. Nat Genet 43: 768–775. 21706001 10.1038/ng.865 PMC3145050

[12] Riester M, Stephan-Otto Attolini C, Downey RJ, Singer S, Michor F (2010) A differentiation-based phylogeny of cancer subtypes. PLoS Comput Biol 6: e1000777 doi:10.1371/journal.pcbi.1000777. 20463876 10.1371/journal.pcbi.1000777 PMC2865519

[13] TIN-HLPFP (1993) A predictive model for aggressive non-Hodgkin's lymphoma. The International Non-Hodgkin's Lymphoma Prognostic Factors Project. New England Journal of Medicine 329: 987–994. 8141877 10.1056/NEJM199309303291402

[14] Uno H, Cai T, Pencina MJ, D'Agostino RB, Wei LJ (2011) On the C-statistics for evaluating overall adequacy of risk prediction procedures with censored survival data. Statistics in Medicine 30: 1105–1116. 21484848 10.1002/sim.4154 PMC3079915

[15] Turker MS (2002) Gene silencing in mammalian cells and the spread of DNA methylation. Oncogene 21: 5388–5393. 12154401 10.1038/sj.onc.1205599

[16] Ahmed I, Sarazin A, Bowler C, Colot V, Quesneville H (2011) Genome-wide evidence for local DNA methylation spreading from small RNA-targeted sequences in Arabidopsis. Nucleic Acids Res 39: 6919–6931. 21586580 10.1093/nar/gkr324 PMC3167636

[17] Hathaway NA, Bell O, Hodges C, Miller EL, Neel DS, et al (2012) Dynamics and memory of heterochromatin in living cells. Cell 149: 1447–1460. 22704655 10.1016/j.cell.2012.03.052 PMC3422694

[18] Cuddapah S, Jothi R, Schones DE, Roh TY, Cui K, et al (2009) Global analysis of the insulator binding protein CTCF in chromatin barrier regions reveals demarcation of active and repressive domains. Genome Res 19: 24–32. 19056695 10.1101/gr.082800.108 PMC2612964

[19] Kim TH, Abdullaev ZK, Smith AD, Ching KA, Loukinov DI, et al (2007) Analysis of the vertebrate insulator protein CTCF-binding sites in the human genome. Cell 128: 1231–1245. 17382889 10.1016/j.cell.2006.12.048 PMC2572726

[20] Ci W, Polo JM, Cerchietti L, Shaknovich R, Wang L, et al (2009) The BCL6 transcriptional program features repression of multiple oncogenes in primary B cells and is deregulated in DLBCL. Blood 113: 5536–5548. 19307668 10.1182/blood-2008-12-193037 PMC2689052

[21] Velichutina I, Shaknovich R, Geng H, Johnson NA, Gascoyne RD, et al (2010) EZH2-mediated epigenetic silencing in germinal center B cells contributes to proliferation and lymphomagenesis. Blood 116: 5247–5255. 20736451 10.1182/blood-2010-04-280149 PMC3012542

[22] Yamane A, Resch W, Kuo N, Kuchen S, Li Z, et al (2011) Deep-sequencing identification of the genomic targets of the cytidine deaminase AID and its cofactor RPA in B lymphocytes. Nat Immunol 12: 62–69. 21113164 10.1038/ni.1964 PMC3005028

[23] Skinnider BF, Horsman DE, Dupuis B, Gascoyne RD (1999) Bcl-6 and Bcl-2 protein expression in diffuse large B-cell lymphoma and follicular lymphoma: correlation with 3q27 and 18q21 chromosomal abnormalities. Hum Pathol 30: 803–808. 10414499 10.1016/s0046-8177(99)90141-7

[24] Cattoretti G, Pasqualucci L, Ballon G, Tam W, Nandula SV, et al (2005) Deregulated BCL6 expression recapitulates the pathogenesis of human diffuse large B cell lymphomas in mice. Cancer Cell 7: 445–455. 15894265 10.1016/j.ccr.2005.03.037

[25] van Galen JC, Dukers DF, Giroth C, Sewalt RG, Otte AP, et al (2004) Distinct expression patterns of polycomb oncoproteins and their binding partners during the germinal center reaction. Eur J Immunol 34: 1870–1881. 15214035 10.1002/eji.200424985

[26] Morin RD, Johnson NA, Severson TM, Mungall AJ, An J, et al (2010) Somatic mutations altering EZH2 (Tyr641) in follicular and diffuse large B-cell lymphomas of germinal-center origin. Nat Genet 42: 181–185. 20081860 10.1038/ng.518 PMC2850970

[27] Suzuki J, Caputo GR, Kondo C, Higgins CB (1990) Cine MR imaging of valvular heart disease: display and imaging parameters affect the size of the signal void caused by valvular regurgitation. AJR Am J Roentgenol 155: 723–727. 2119099 10.2214/ajr.155.4.2119099

[28] Rimsza LM, Leblanc ML, Unger JM, Miller TP, Grogan TM, et al (2008) Gene expression predicts overall survival in paraffin-embedded tissues of diffuse large B-cell lymphoma treated with R-CHOP. Blood 112: 3425–3433. 18544678 10.1182/blood-2008-02-137372 PMC4467875

[29] Xu Z, Pone EJ, Al-Qahtani A, Park SR, Zan H, et al (2007) Regulation of aicda expression and AID activity: relevance to somatic hypermutation and class switch DNA recombination. Crit Rev Immunol 27: 367–397. 18197815 10.1615/critrevimmunol.v27.i4.60 PMC2994649

[30] Guo JU, Su Y, Zhong C, Ming GL, Song H (2011) Hydroxylation of 5-Methylcytosine by TET1 Promotes Active DNA Demethylation in the Adult Brain. Cell 145: 423–434. 21496894 10.1016/j.cell.2011.03.022 PMC3088758

[31] Bhutani N, Brady JJ, Damian M, Sacco A, Corbel SY, et al (2010) Reprogramming towards pluripotency requires AID-dependent DNA demethylation. Nature 463: 1042–1047. 20027182 10.1038/nature08752 PMC2906123

[32] Weisenberger DJ, Velicescu M, Cheng JC, Gonzales FA, Liang G, et al (2004) Role of the DNA methyltransferase variant DNMT3b3 in DNA methylation. Mol Cancer Res 2: 62–72. 14757847

[33] Angrand PO, Apiou F, Stewart AF, Dutrillaux B, Losson R, et al (2001) NSD3, a new SET domain-containing gene, maps to 8p12 and is amplified in human breast cancer cell lines. Genomics 74: 79–88. 11374904 10.1006/geno.2001.6524

[34] Morishita M, di Luccio E (2011) Cancers and the NSD family of histone lysine methyltransferases. Biochim Biophys Acta 1816: 158–163. 21664949 10.1016/j.bbcan.2011.05.004

[35] Kang D, Cho HS, Toyokawa G, Kogure M, Yamane Y, et al (2012) The histone methyltransferase Wolf-Hirschhorn syndrome candidate 1-like 1 (WHSC1L1) is involved in human carcinogenesis. Genes Chromosomes Cancer 10.1002/gcc.22012 23011637

[36] Attia M, Rachez C, De Pauw A, Avner P, Rogner UC (2007) Nap1l2 promotes histone acetylation activity during neuronal differentiation. Mol Cell Biol 27: 6093–6102. 17591696 10.1128/MCB.00789-07 PMC1952155

[37] Leung GP, Lee L, Schmidt TI, Shirahige K, Kobor MS (2011) Rtt107 is required for recruitment of the SMC5/6 complex to DNA double strand breaks. J Biol Chem 286: 26250–26257. 21642432 10.1074/jbc.M111.235200 PMC3138301

[38] Roy MA, D'Amours D (2011) DNA-binding properties of Smc6, a core component of the Smc5-6 DNA repair complex. Biochem Biophys Res Commun 416: 80–85. 22086171 10.1016/j.bbrc.2011.10.149

[39] Lai AY, Fatemi M, Dhasarathy A, Malone C, Sobol SE, et al (2010) DNA methylation prevents CTCF-mediated silencing of the oncogene BCL6 in B cell lymphomas. J Exp Med 207: 1939–1950. 20733034 10.1084/jem.20100204 PMC2931164

[40] Thompson RF, Reimers M, Khulan B, Gissot M, Richmond TA, et al (2008) An analytical pipeline for genomic representations used for cytosine methylation studies. Bioinformatics 24: 1161–1167. 18353789 10.1093/bioinformatics/btn096 PMC5061929

[41] Fujita PA, Rhead B, Zweig AS, Hinrichs AS, Karolchik D, et al (2011) The UCSC Genome Browser database: update 2011. Nucleic Acids Res 39: D876–882. 20959295 10.1093/nar/gkq963 PMC3242726

[42] Flicek P, Amode MR, Barrell D, Beal K, Brent S, et al (2011) Ensembl 2011. Nucleic Acids Res 39: D800–806. 21045057 10.1093/nar/gkq1064 PMC3013672

[43] Oberley MJ, Tsao J, Yau P, Farnham PJ (2004) High-throughput screening of chromatin immunoprecipitates using CpG-island microarrays. Methods Enzymol 376: 315–334. 14975315 10.1016/S0076-6879(03)76021-2

[44] Mukhopadhyay R, Yu W, Whitehead J, Xu J, Lezcano M, et al (2004) The binding sites for the chromatin insulator protein CTCF map to DNA methylation-free domains genome-wide. Genome Res 14: 1594–1602. 15256511 10.1101/gr.2408304 PMC509268

