# Supplementary figures and images for: Aberration in DNA Methylation in B-Cell Lymphomas Has a Complex Origin and Increases with Disease Severity
Source: PLoS Genet. 2013 Jan 10;9(1):e1003137. doi: 10.1371/journal.pgen.1003137 (PMC3542081; doi:10.1371/journal.pgen.1003137)

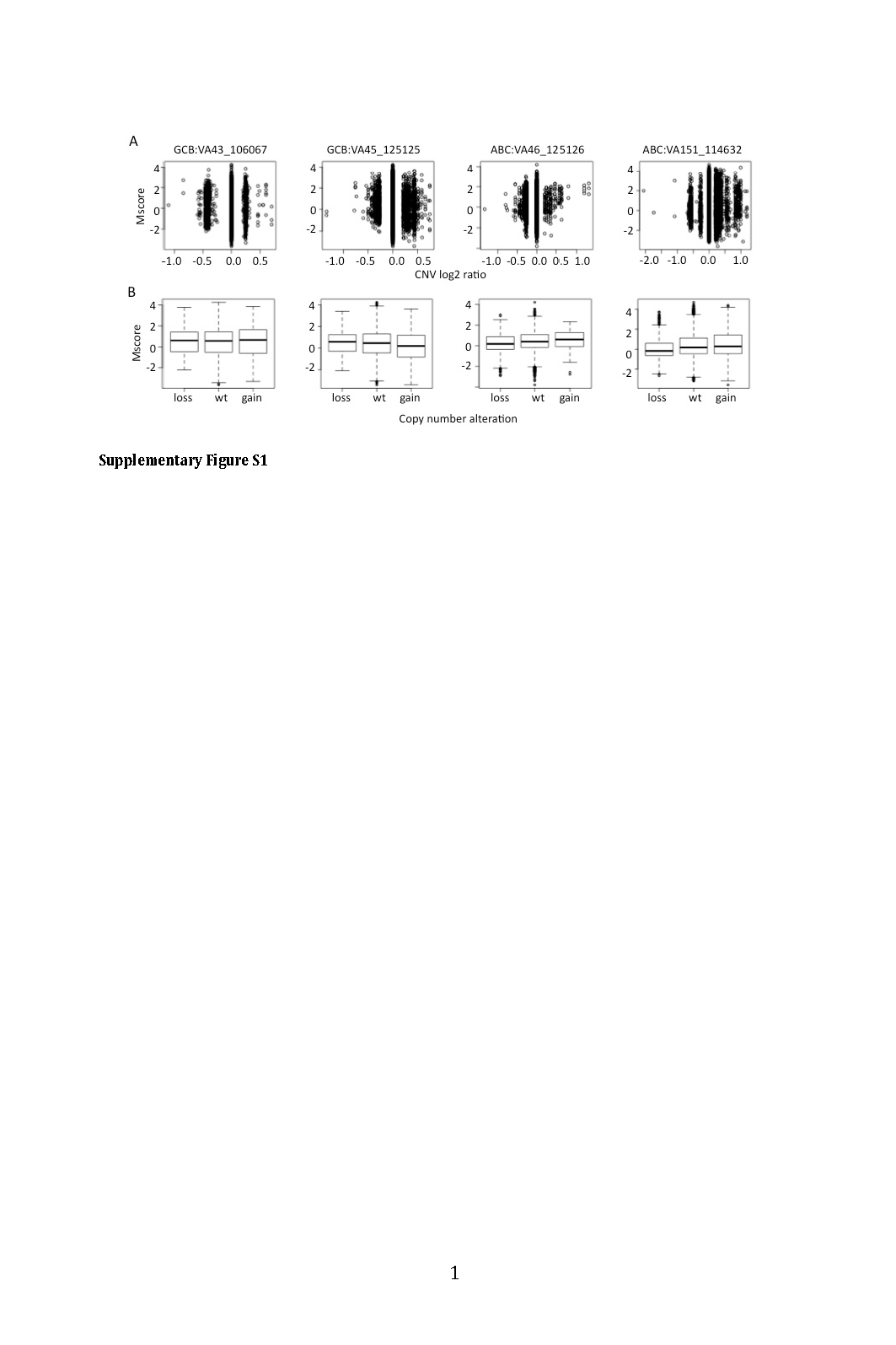

Supplement: Figure S1 — Copy number analyses. (A) Distributions of M-scores against copy number log2 ratios for two GCB and two ABC samples. (B) Boxplots showing the distributions of M-scores against copy number loss, gain, and wild type (wt) for those GCB and ABC samples. (TIF) [file pgen.1003137.s001.tif]

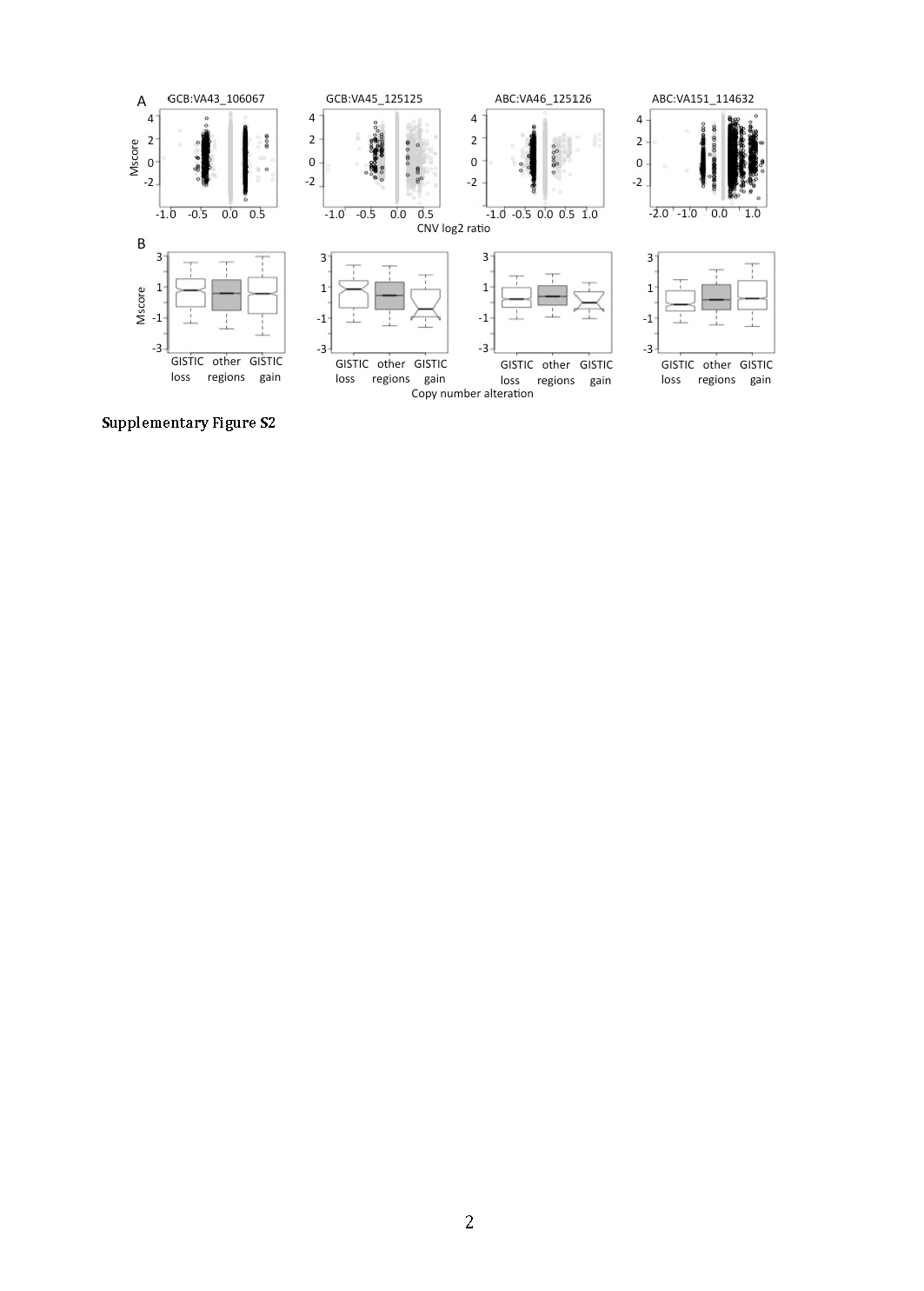

Supplement: Figure S2 — Copy number analyses for frequently amplified or deleted regions. (A) The distributions of M-scores against copy number log2 ratios for two GCB and two ABC samples. DNA promoter methylation probes in the regions that were amplified or deleted in a given sample, and also overlapped with GISTIC peaks, are shown in black, and remaining probes are shown in grey. (B) Boxplots showing the distributions of M-scores against copy number loss, gain, and wild type for those GCB and ABC samples. (TIF) [file pgen.1003137.s002.tif]

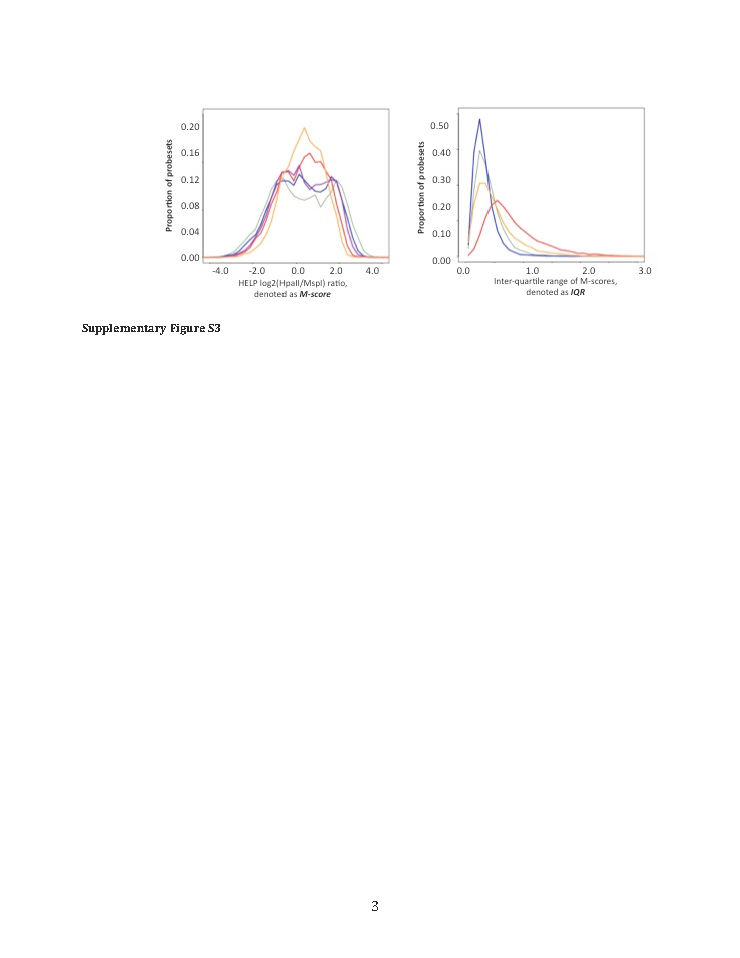

Supplement: Figure S3 — The frequency distribution of (left) the median M-score and (right) inter-quartile ranges (IQR) of the M-score, per methylation probe of gene promoters for normal and diseased samples with ≥80% purity. The color code is similar to that of Figure 1A in the main text. (TIF) [file pgen.1003137.s003.tif]

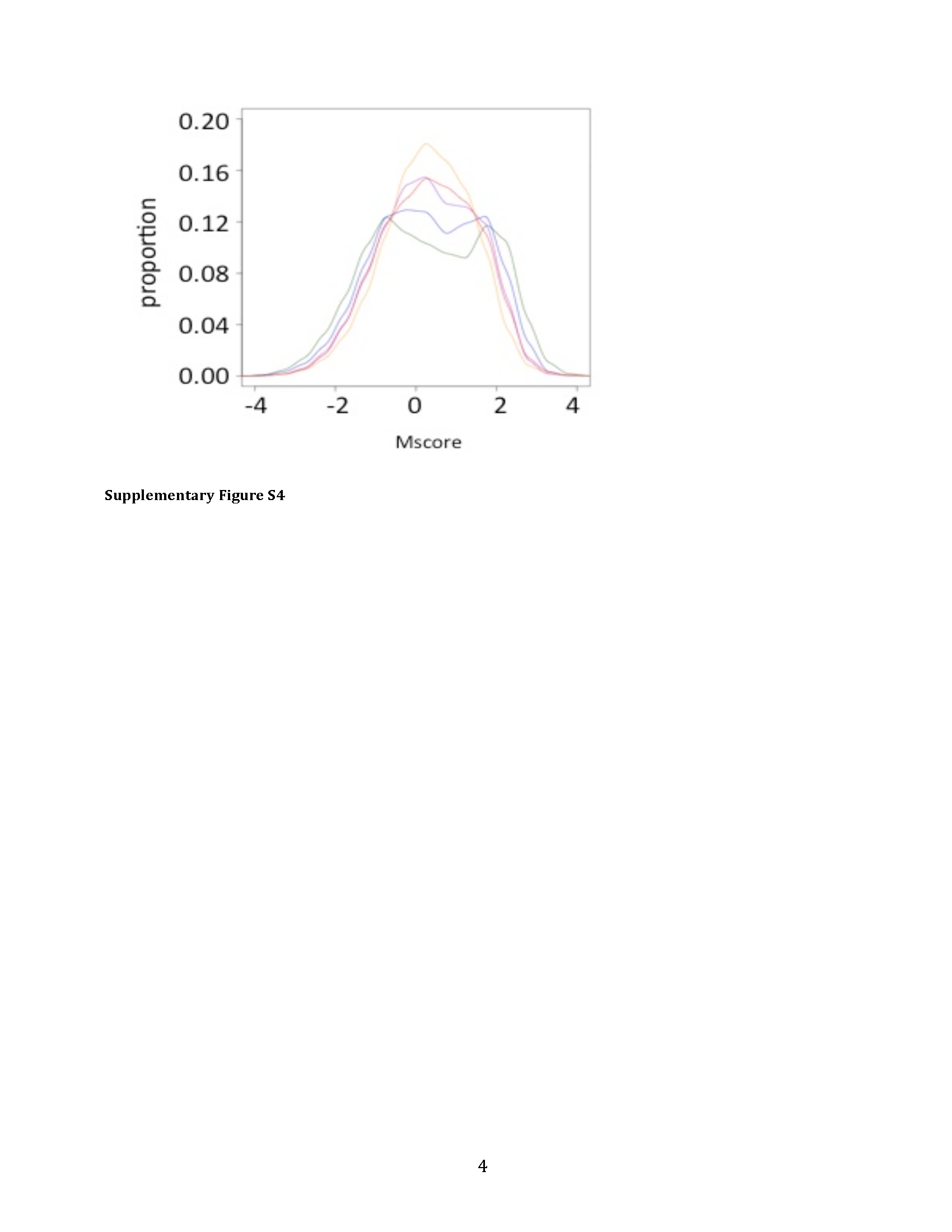

Supplement: Figure S4 — Frequency distribution of M-scores at the genome-wide methylation probe positions. Data is shown after removal of low signal to noise ratio probes. Color codes are the same as in Figure 1. (TIF) [file pgen.1003137.s004.tif]

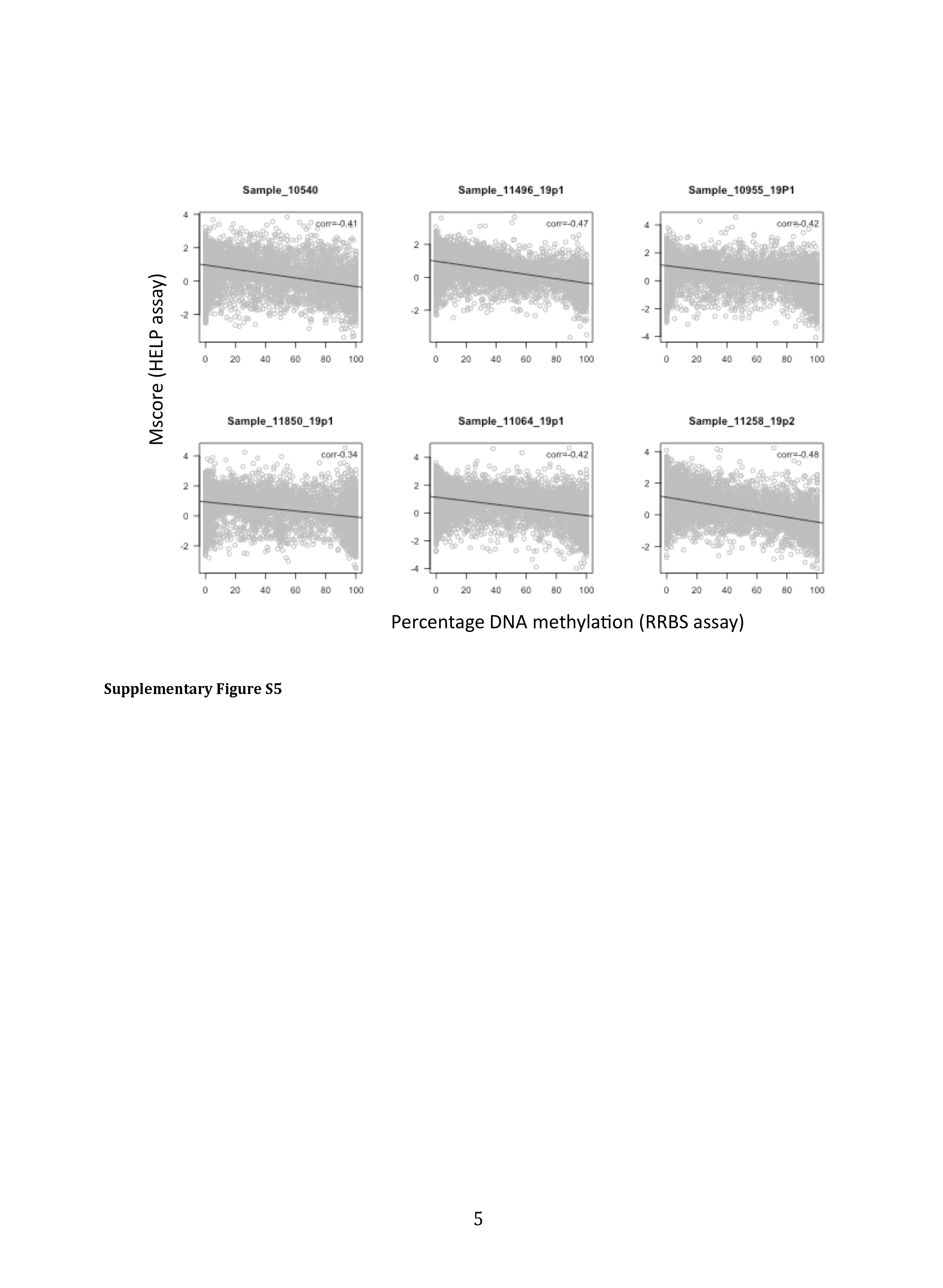

Supplement: Figure S5 — Scatter plot showing the M-score as reported by the HELP assay and the percentage DNA methylation as reported by the RRBS assay for 6 samples. Pearson correlation coefficient (top right corner) and regression lines are shown for each panel. (TIF) [file pgen.1003137.s005.tif]

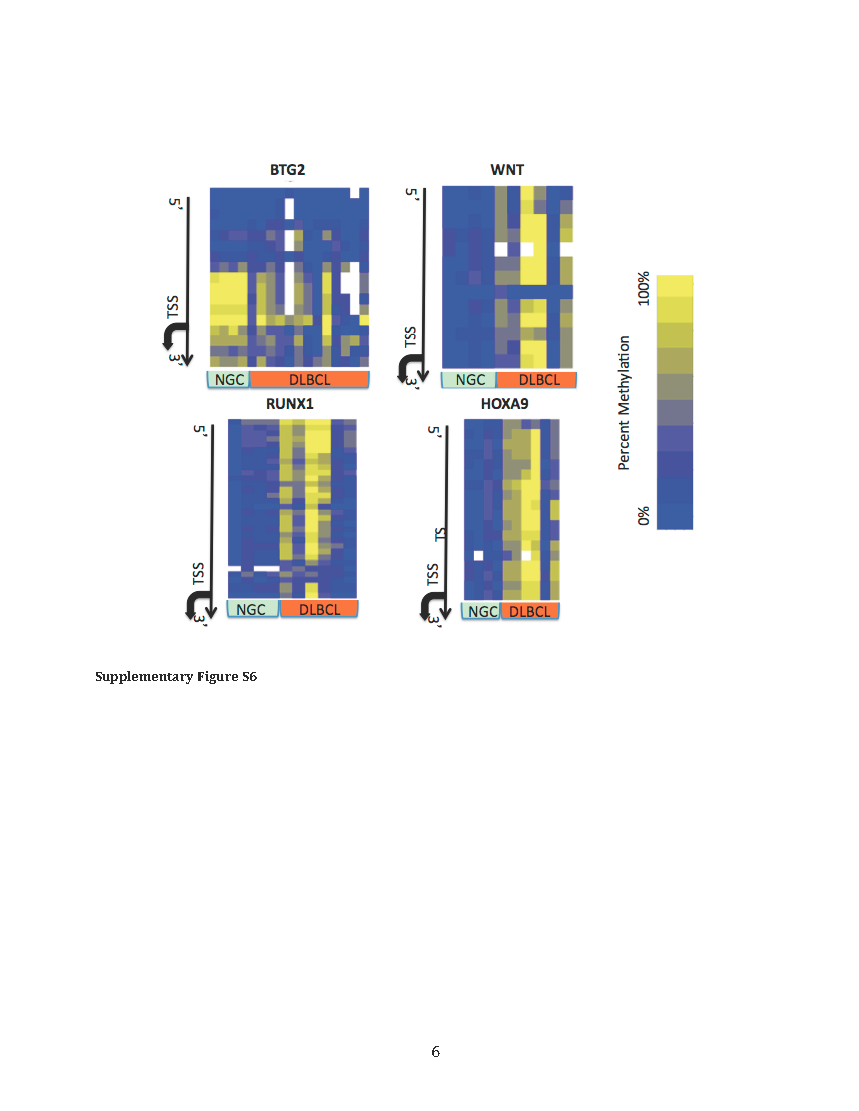

Supplement: Figure S6 — MassARRAY validation shows that there is greater variance in methylation within DLBCL samples than in normal B-cell samples. (TIF) [file pgen.1003137.s006.tif]

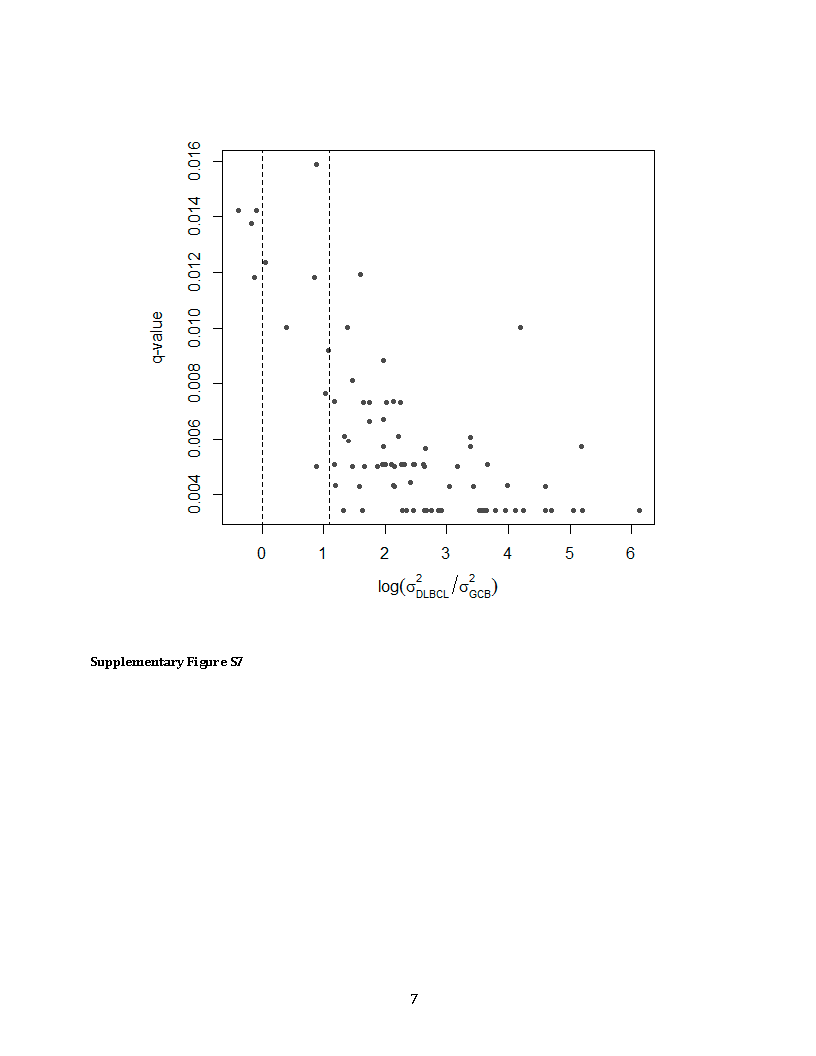

Supplement: Figure S7 — Log(variance ratio) vs. q-value plot demonstrates greater variance of CpG methylation values derived from MassARRAY validation. Only 4 CpGs had a lower variance in DLBCL but the remaining CpGs had a higher variance in DLBCL as compared to NGC samples. The dashed line on the left represents equal variance between the two groups. The dashed line on the right represents 3× higher variance in DLBCL as compared to NGC. (TIF) [file pgen.1003137.s007.tif]

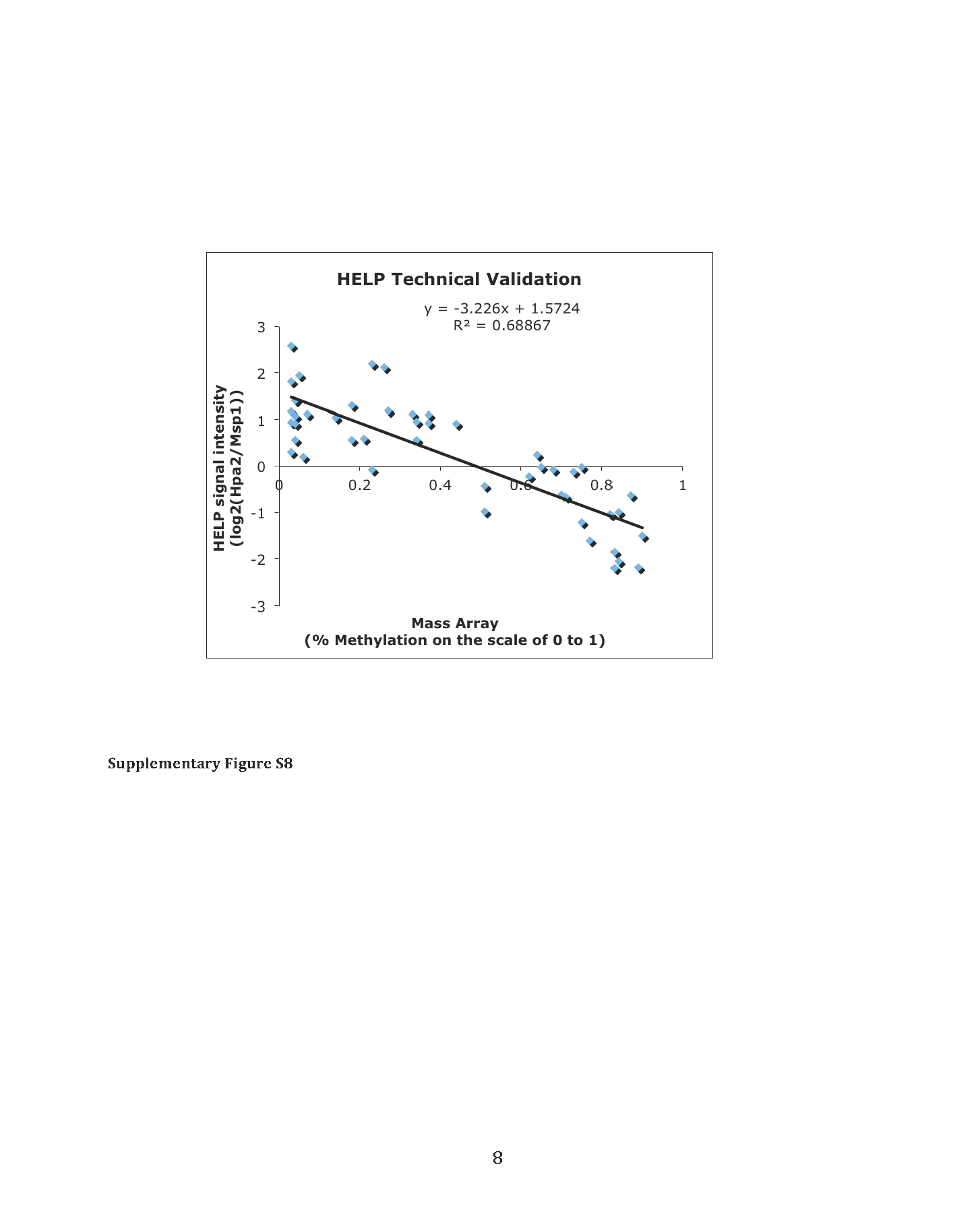

Supplement: Figure S8 — Technical validation of the HELP array using MassARRAY Epityping reveals a linear relationship between these two assays. These validation studies revealed that 1 unit of log2(HpaII/MspI) change in HELP intensity corresponds to 30% change in methylation as detected by the MassARRAY. (TIF) [file pgen.1003137.s008.tif]

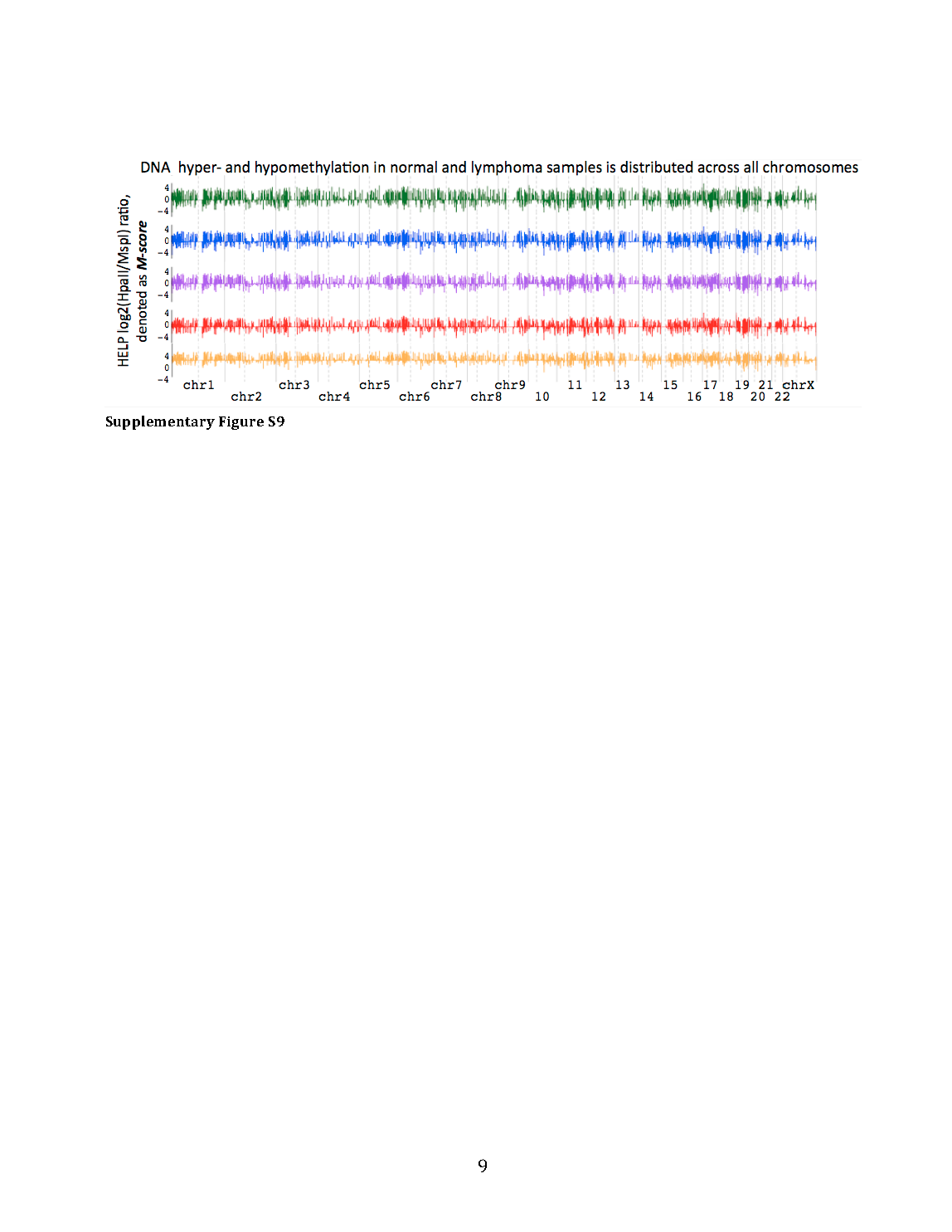

Supplement: Figure S9 — Distribution of the sites of hypo- and hyper-methylation along the human chromosomes in both normal and lymphoma samples. The color code is similar to that of Figure 1A in the main text. (TIF) [file pgen.1003137.s009.tif]

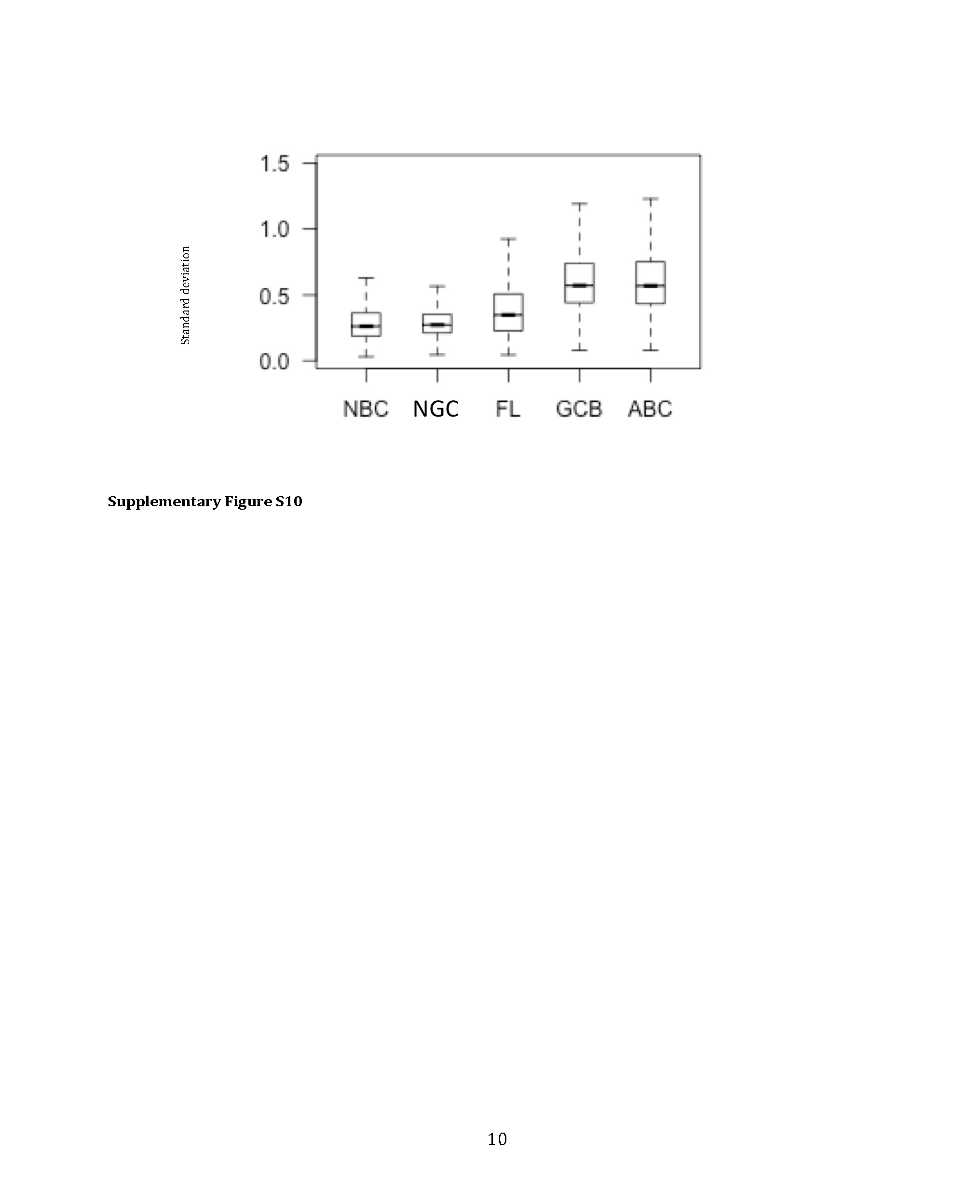

Supplement: Figure S10 — Distribution of inter-sample standard deviation of M-score, grouped by NBC, NGC, FL, GCB and ABC. Outliers are not shown. (TIF) [file pgen.1003137.s010.tif]

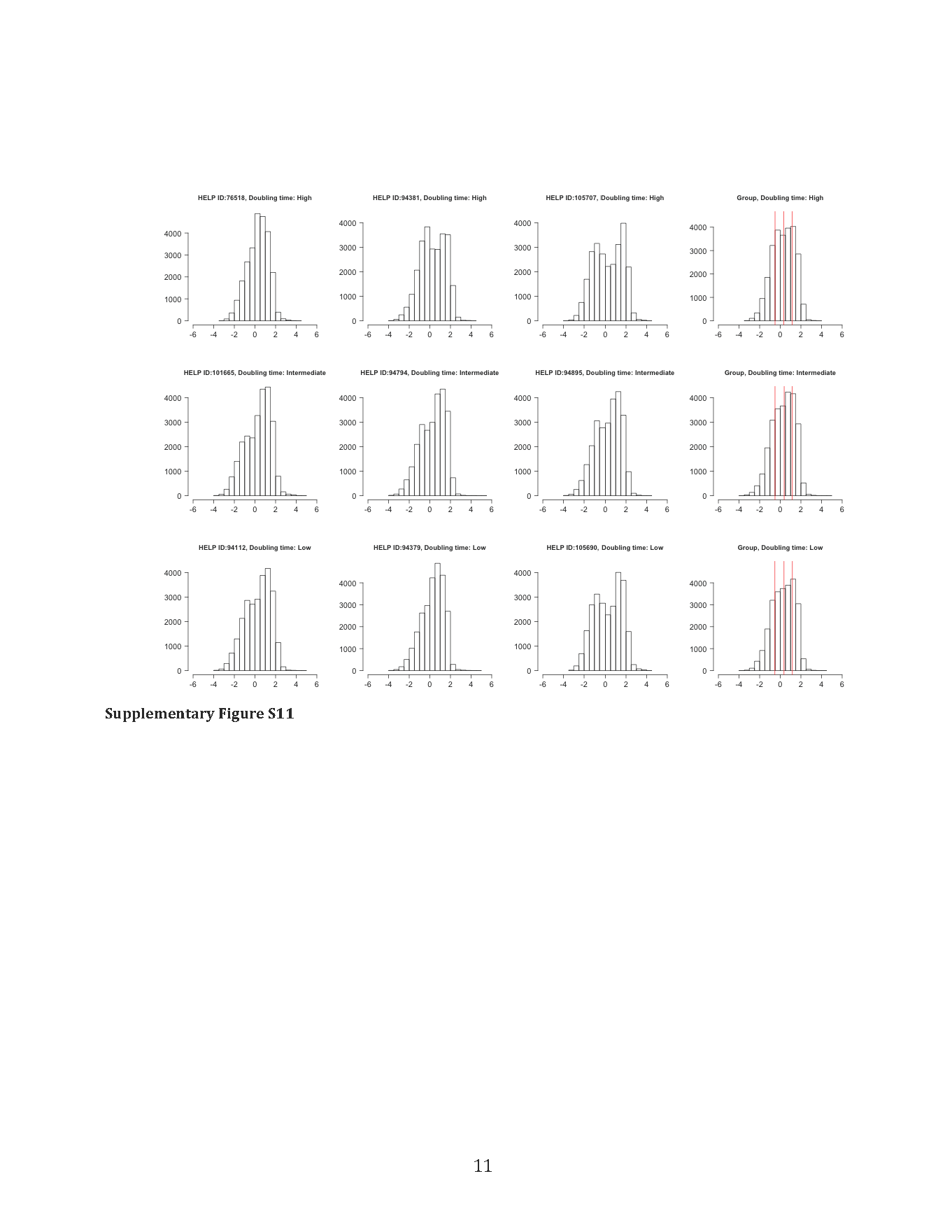

Supplement: Figure S11 — Distribution of M-score of the cell lines which are grouped into three categories – low, intermediate and high, based on their doubling times. In each panel, the X-axis represents the M-score, and the Y-axis represents the frequency of promoter methylation probe sets which have that M-score. The HELP ID of the cell lines and their doubling time are provided at the top of each panel. The last column represents the distribution of the M-score for the group average. Median and the two quantiles are highlighted in red. (TIF) [file pgen.1003137.s011.tif]

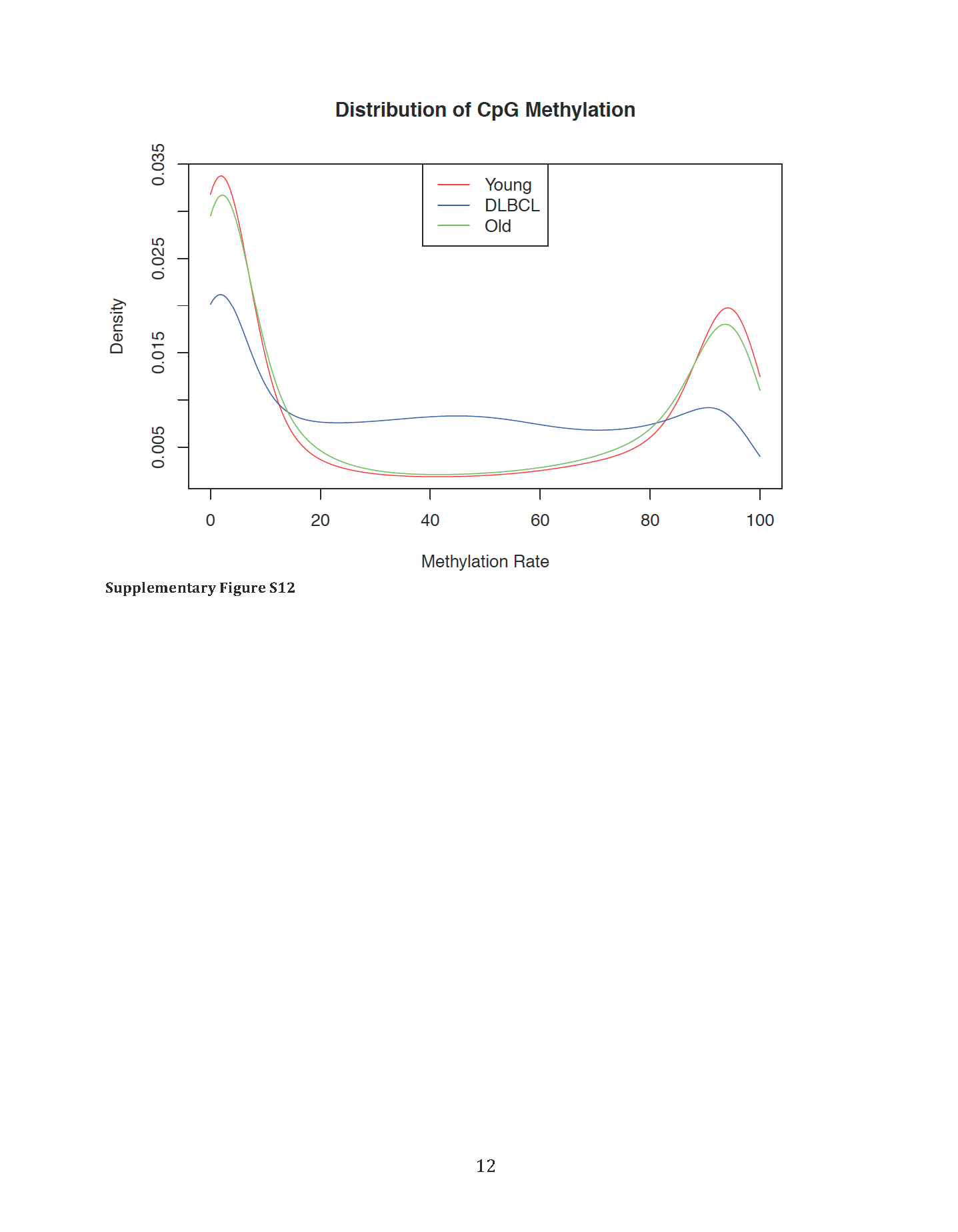

Supplement: Figure S12 — Distribution of % methylation using the eRRBS assay at CpG sites in young and old B-cell controls and in DLBCLs. (TIF) [file pgen.1003137.s012.tif]

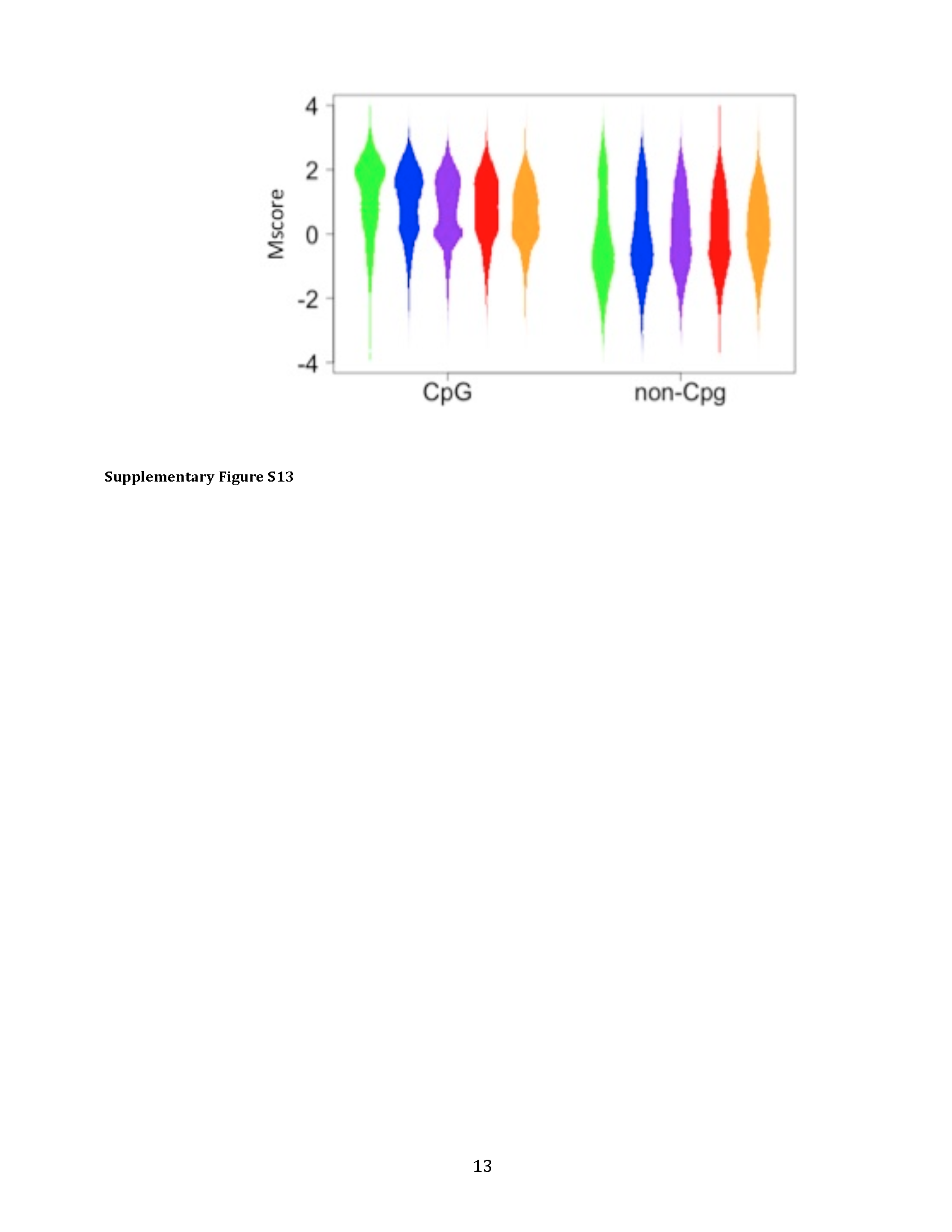

Supplement: Figure S13 — Distributions of M-scores at the methylation probesets overlapping with CpG islands and non-CpG islands for normal B-cell and DLBCL samples. The color code is as following: NBC-green, NGC-blue, FL-purple, GCB-red and ABC-orange. For each sample type, bar width is proportional to the number of probes with a given M-score, as discussed in details in Figure 3A in the main text. (TIF) [file pgen.1003137.s013.tif]

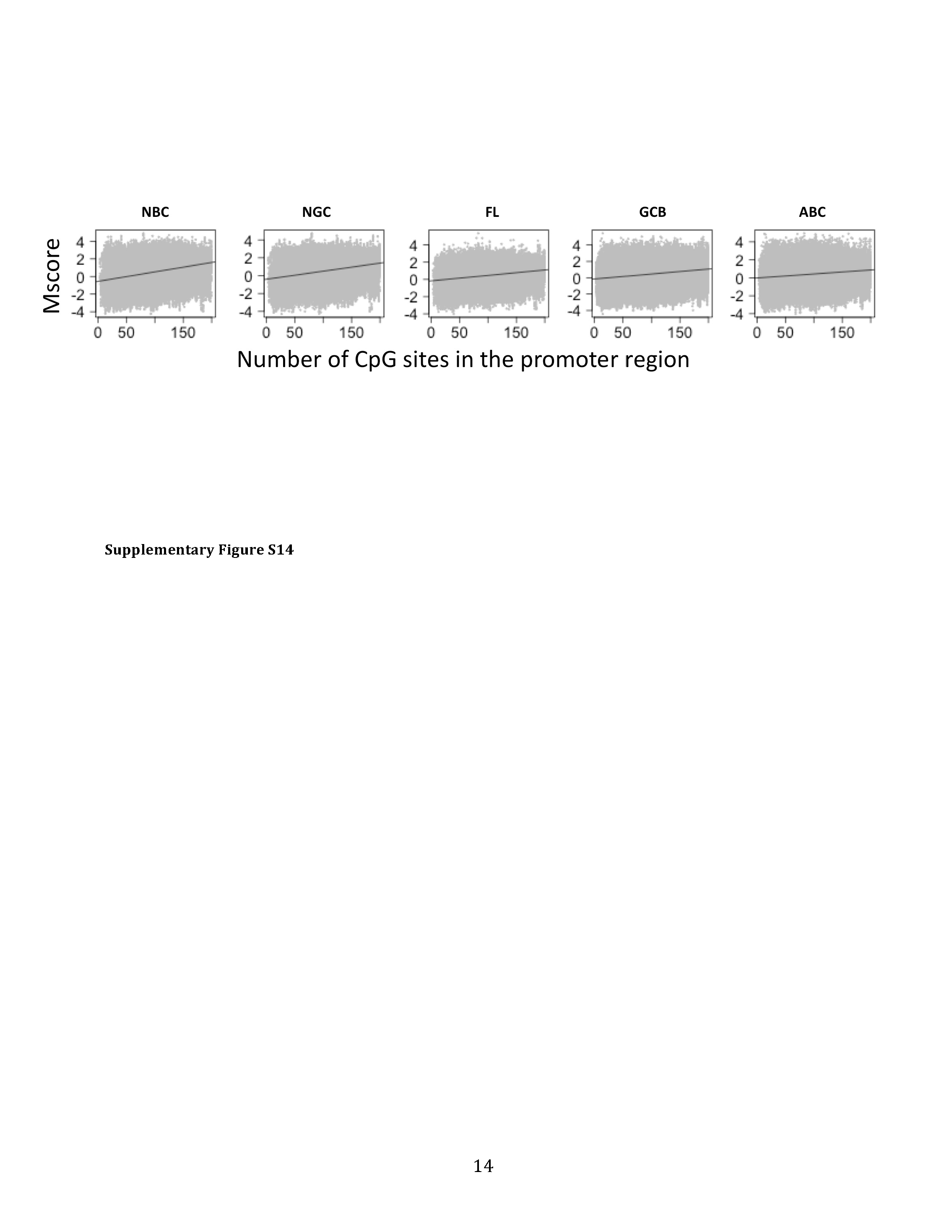

Supplement: Figure S14 — Distribution of M-score against CpG density at gene promoters for NBC, NGC, FL, GCB, and ABC samples. The regression line for each category is shown in black. (TIF) [file pgen.1003137.s014.tif]

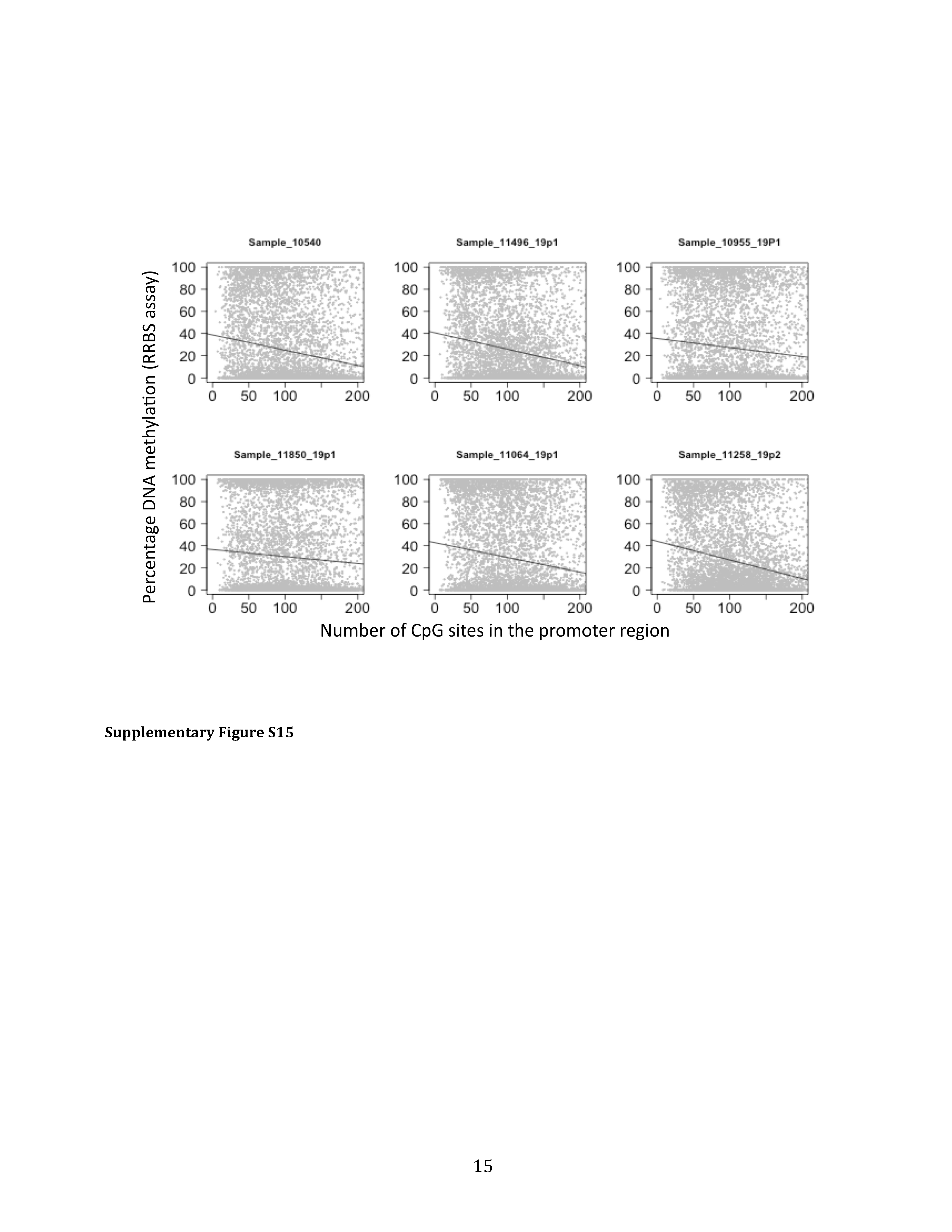

Supplement: Figure S15 — Distribution of the percentage DNA methylation as estimated for the number of CpG sites in the gene promoters for 6 DLBCL samples. The regression line for each category is shown in black. (TIF) [file pgen.1003137.s015.tif]

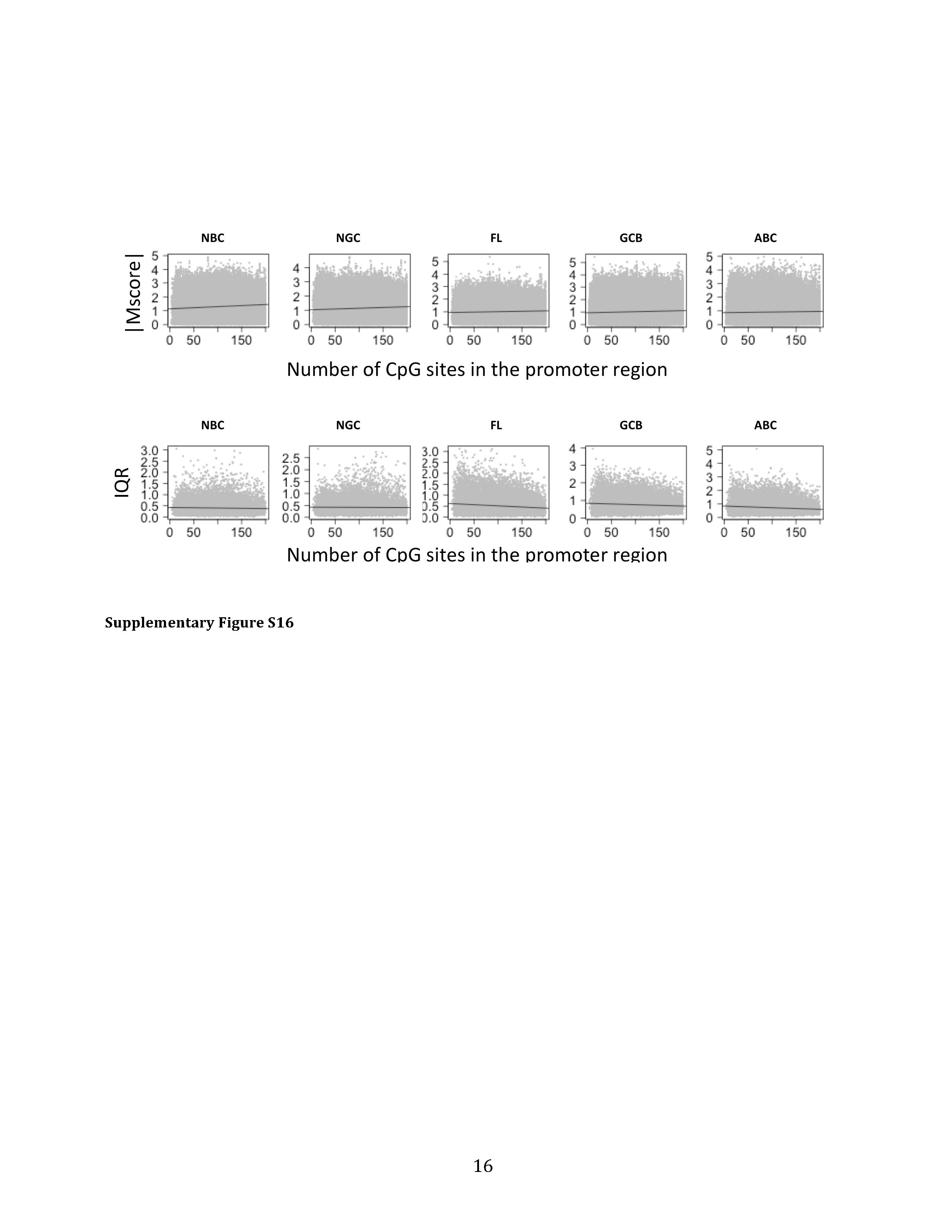

Supplement: Figure S16 — Distribution of (i) |M-score| (y-axis, top) and (ii) IQR (y axis, bottom) against CpG density at gene promoters for NBC, NGC, FL, GCB, ABC samples. The regression line for each category is shown in black. (TIF) [file pgen.1003137.s016.tif]

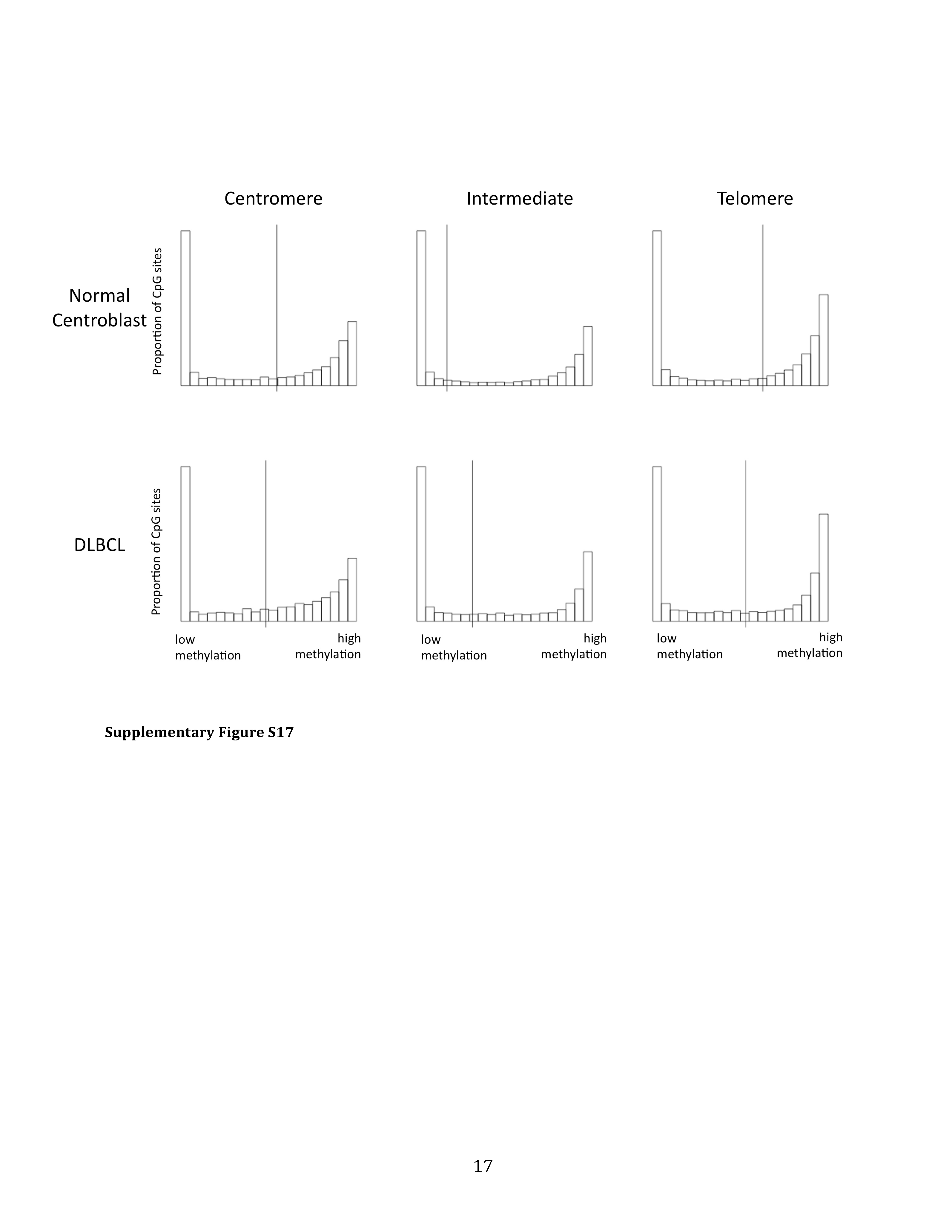

Supplement: Figure S17 — Distribution of % methylation using the eRRBS assay at CpG sites in centromeric, telomeric, and intermediate regions for normal and diseased tissues. In each panel, the vertical bar represents the median value of the respective distribution. (TIF) [file pgen.1003137.s017.tif]

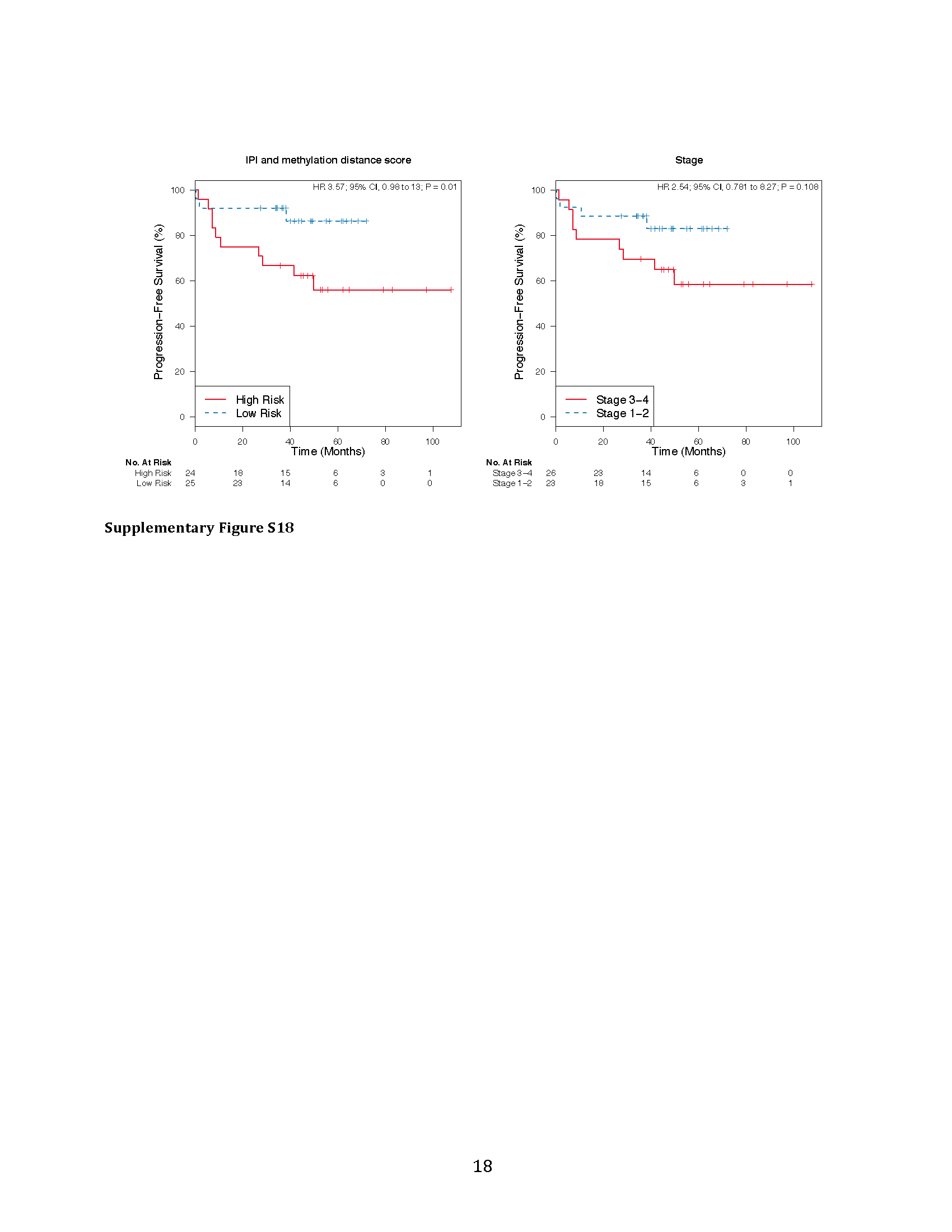

Supplement: Figure S18 — Kaplan-Meier comparison of the risk stratification by stage and methylation heterogeneity score (MHS; left) and stage alone (right) in ABC and GCB. (TIF) [file pgen.1003137.s018.tif]

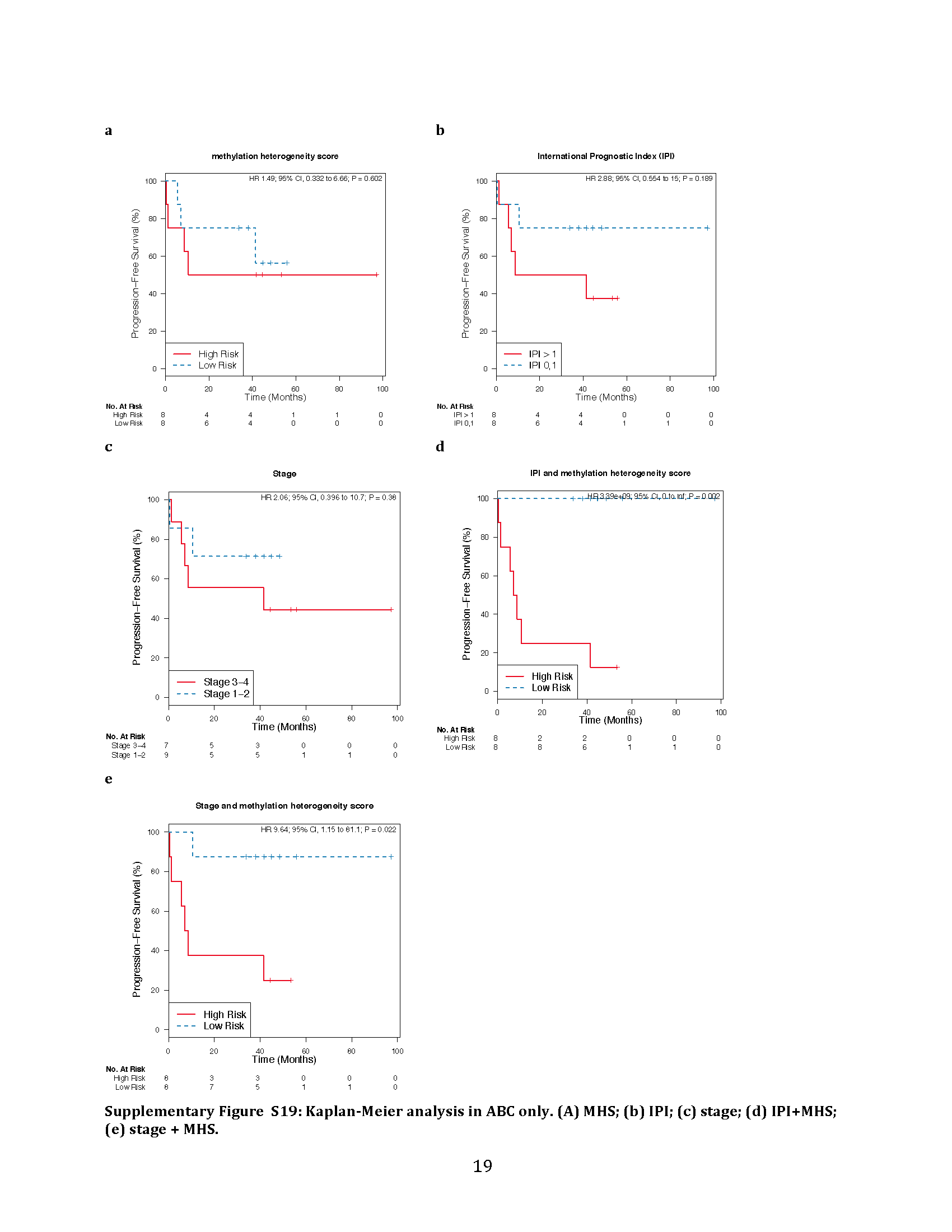

Supplement: Figure S19 — Kaplan-Meier analysis in ABC only. (A) MHS; (b) IPI; (c) stage; (d) IPI+MHS; (e) stage+MHS. (TIF) [file pgen.1003137.s019.tif]

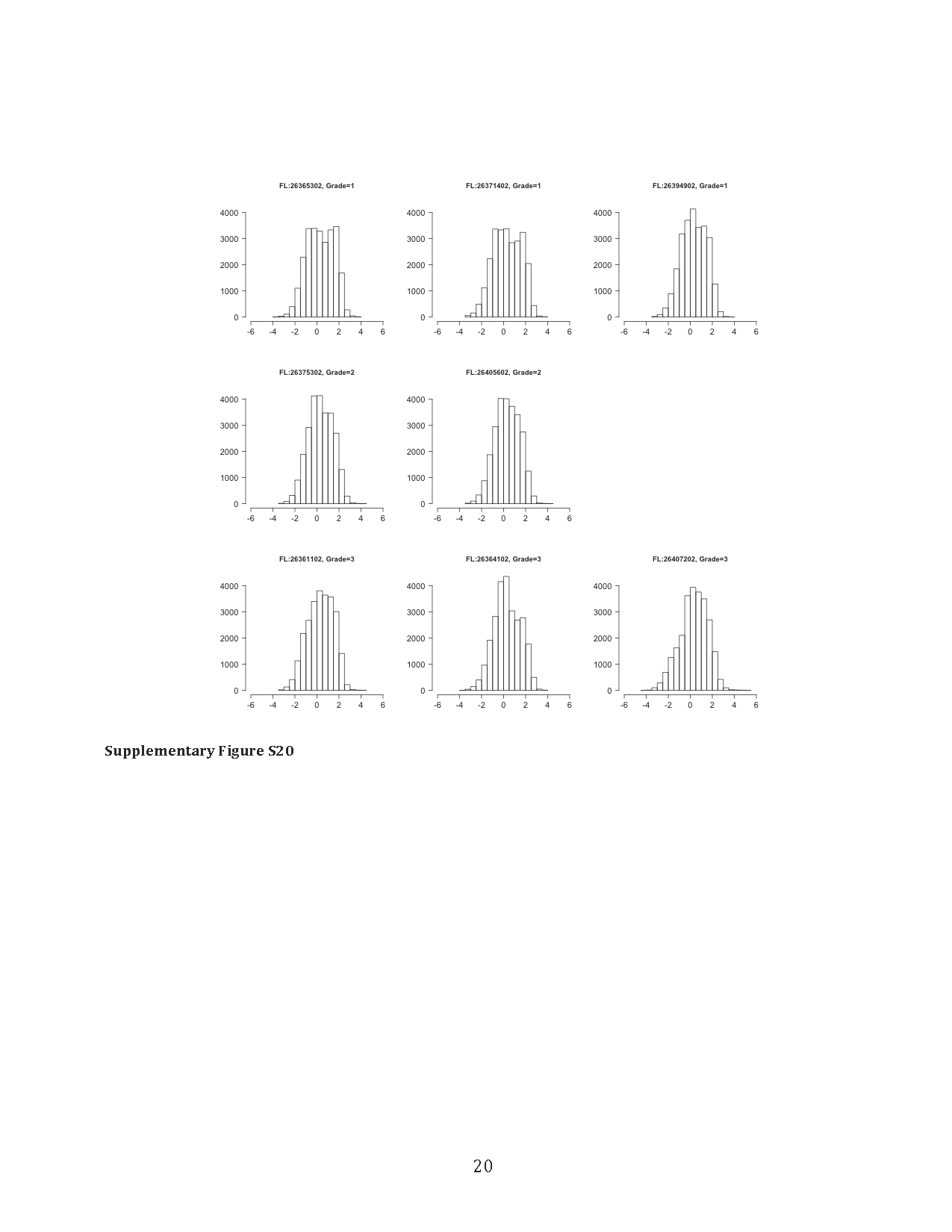

Supplement: Figure S20 — Distribution of M-score of the FL samples which are grouped according to their grades. In each panel, the X-axis represents the M-score and the Y-axis represents the frequency of promoter methylation probe sets, which have that M-score. The HELP ID of the cell lines and their grade are provided at the top of each panel. (TIF) [file pgen.1003137.s020.tif]

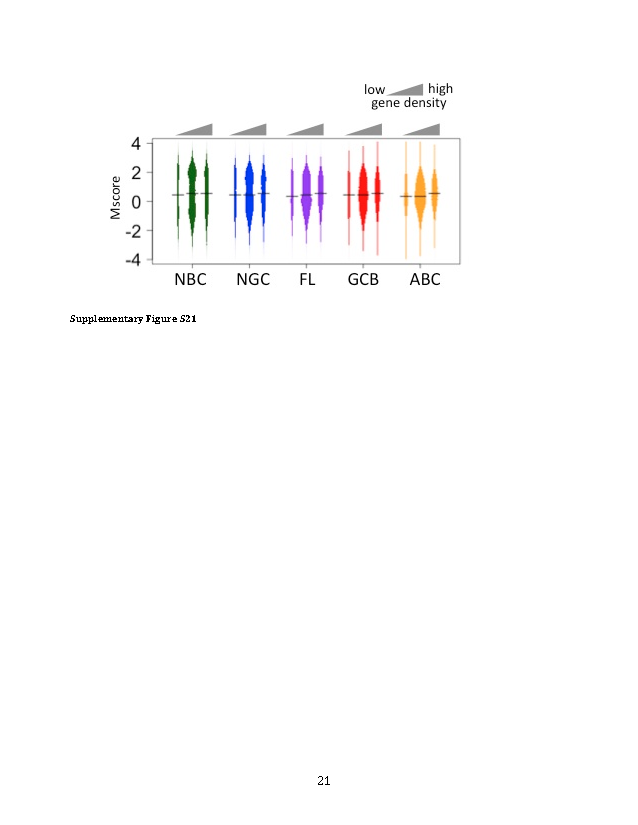

Supplement: Figure S21 — Distributions of M-score for gene-poor, intermediate, and gene-rich regions for normal and lymphoma samples. Color codes are the same as in Figure 1. Bar width is proportional to the number of probes with a given M-score, as discussed in details in Figure 3A in the main text. (TIF) [file pgen.1003137.s021.tif]

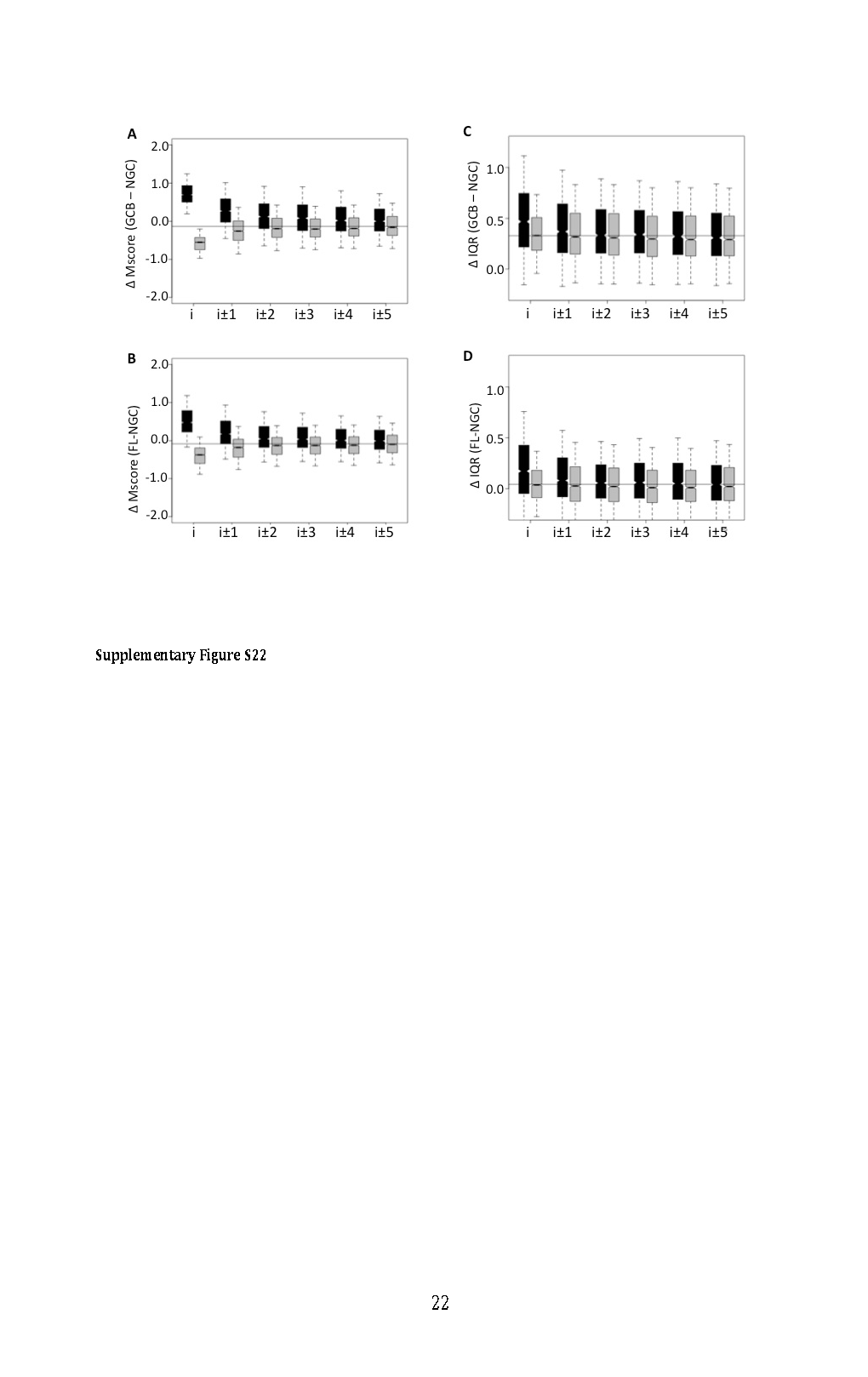

Supplement: Figure S22 — Spreading of aberrant methylation in neighboring positions. Position “i” represents probes that are significantly hypo- (black) or hyper-methylated (grey) in lymphoma samples compared to normal tissues, and “i±n” represents the nth neighbors of those probes. The difference in M-score (A and B) and the difference in between-sample variation, estimated by IQR (C and D) between the lymphoma and normal samples at “i”, “i±1”, … “i±5” positions are shown. (TIF) [file pgen.1003137.s022.tif]

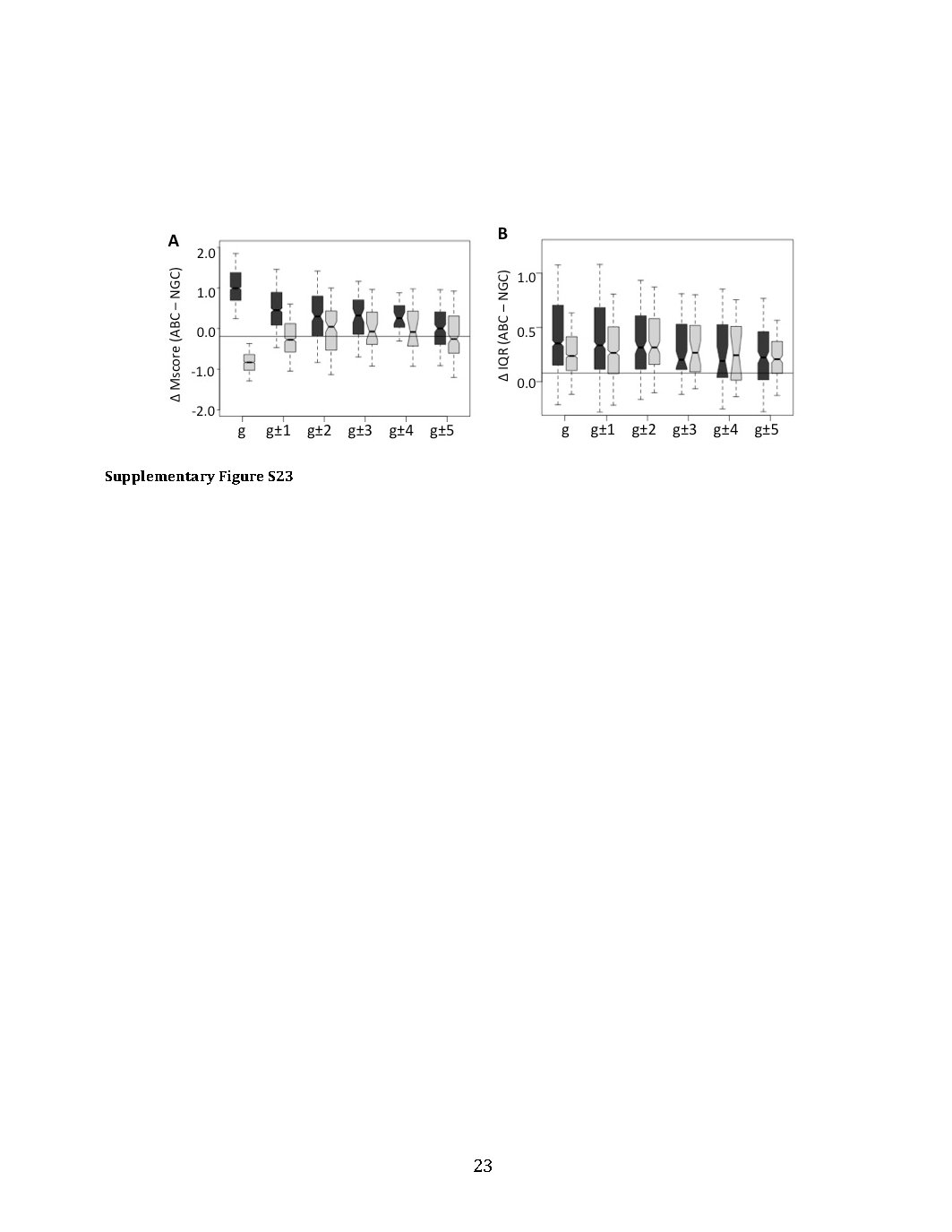

Supplement: Figure S23 — Spreading of aberrant methylation in neighboring gene promoters after excluding probesets that map to multiple genes or genes marked by multiple probesets. Position “g” represents probes that are significantly hypo- (black) or hyper-methylated (grey) in lymphoma samples compared to normal tissues, and “g±n” represents the n-th neighboring promoter probeset. The difference in M-score (A) and the difference in between-sample variation, estimated by IQR (B) between the lymphoma and normal samples at “g”, “g±1”, … “g±5” positions are shown. (TIF) [file pgen.1003137.s023.tif]

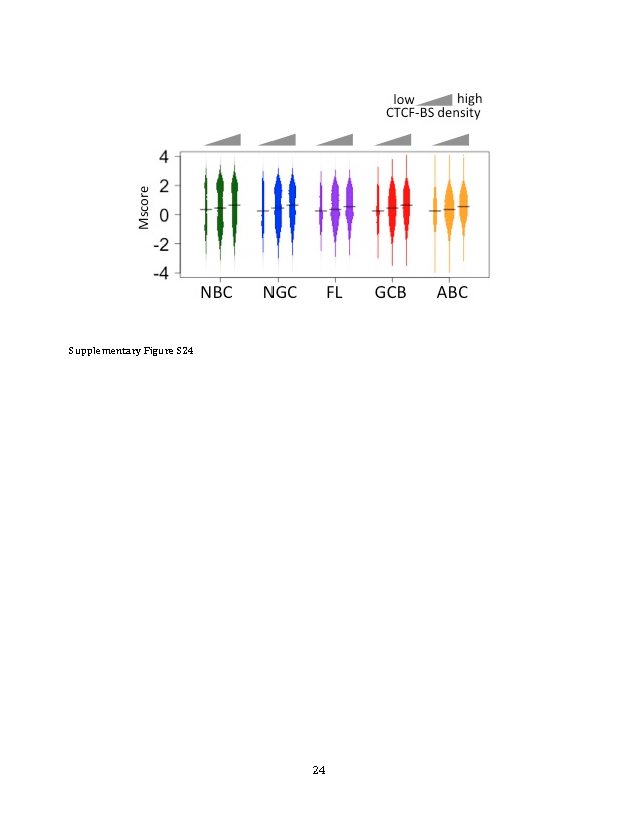

Supplement: Figure S24 — Distributions of M-score for regions with low CTCF-BS density, intermediate, and high CTCF-BS density in normal and lymphoma samples. Color codes are the same as in Figure 1. Bar width is proportional to the number of probes with a given M-score as discussed in detail in the main text. (TIF) [file pgen.1003137.s024.tif]

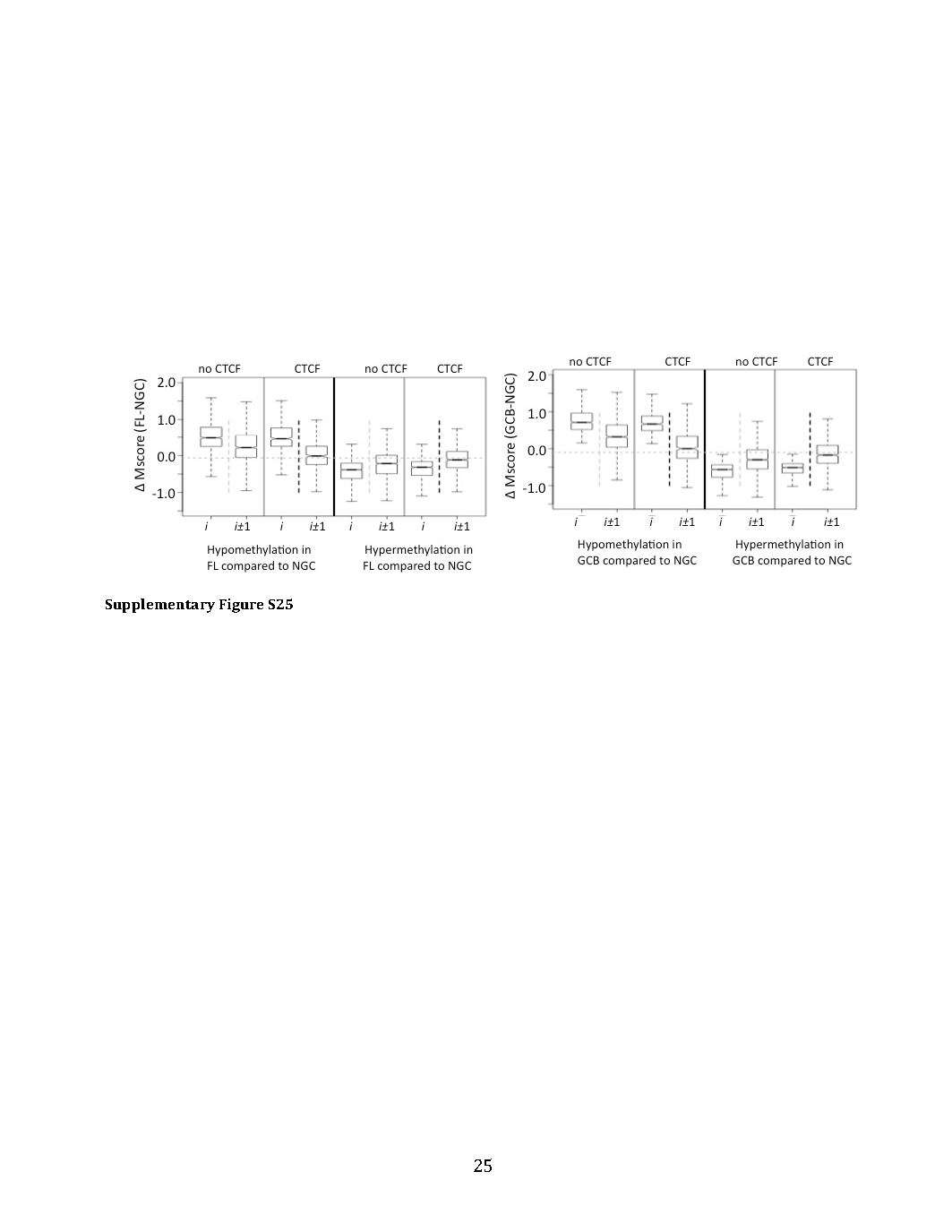

Supplement: Figure S25 — Effects of CTCF-binding site on spreading of aberrant methylation in (left) FL and (right) GCB samples. Locus “i” refers to a promoter probe position that has significantly different methylation pattern in ABC compared to NGC, and “i±1” represent its immediate up- and downstream neighboring probe positions. Changes in M-score at “i” and “i±1” in the lymphoma samples relative to NGC samples were calculated for four different scenarios – depending on whether “i” had aberrant hypo- or hyper-methylation, and presence (black vertical dotted line) or absence (light grey vertical dotted line) of CTCF-BS between “i” and “i+1”. The horizontal line represents the genome-wide median Δ M-score between ABC and NGC samples. We found that the presence of CTCF-BS restricted the spreading of aberrant hypo- or hyper-methylation. (TIF) [file pgen.1003137.s025.tif]

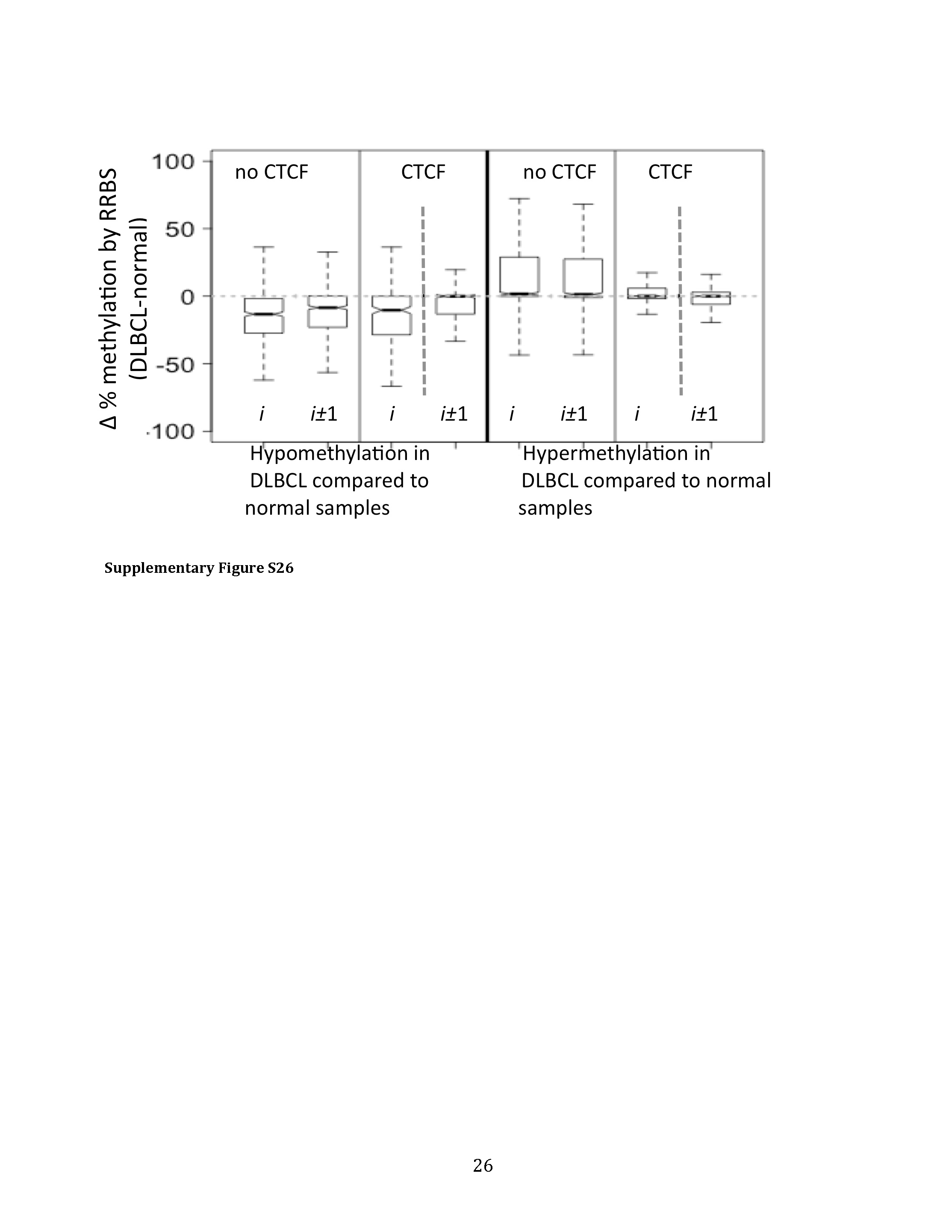

Supplement: Figure S26 — Effects of CTCF-binding sites (BS) on the spreading of aberrant methylation in DLBCL samples. Locus “i” refers to a promoter probe position that has significantly different methylation pattern in DLBCL compared to NGC, and “i±1” represent its immediate up- and downstream neighboring positions. Changes in % methylation at “i” and “i±1” in the lymphoma samples relative to NGC samples were calculated for four different scenarios – depending on whether “i” had aberrant hypo- or hyper-methylation, and presence (black vertical dotted line) or absence (light grey vertical dotted line) of CTCF-BS between “i” and “i+1”. (TIF) [file pgen.1003137.s026.tif]

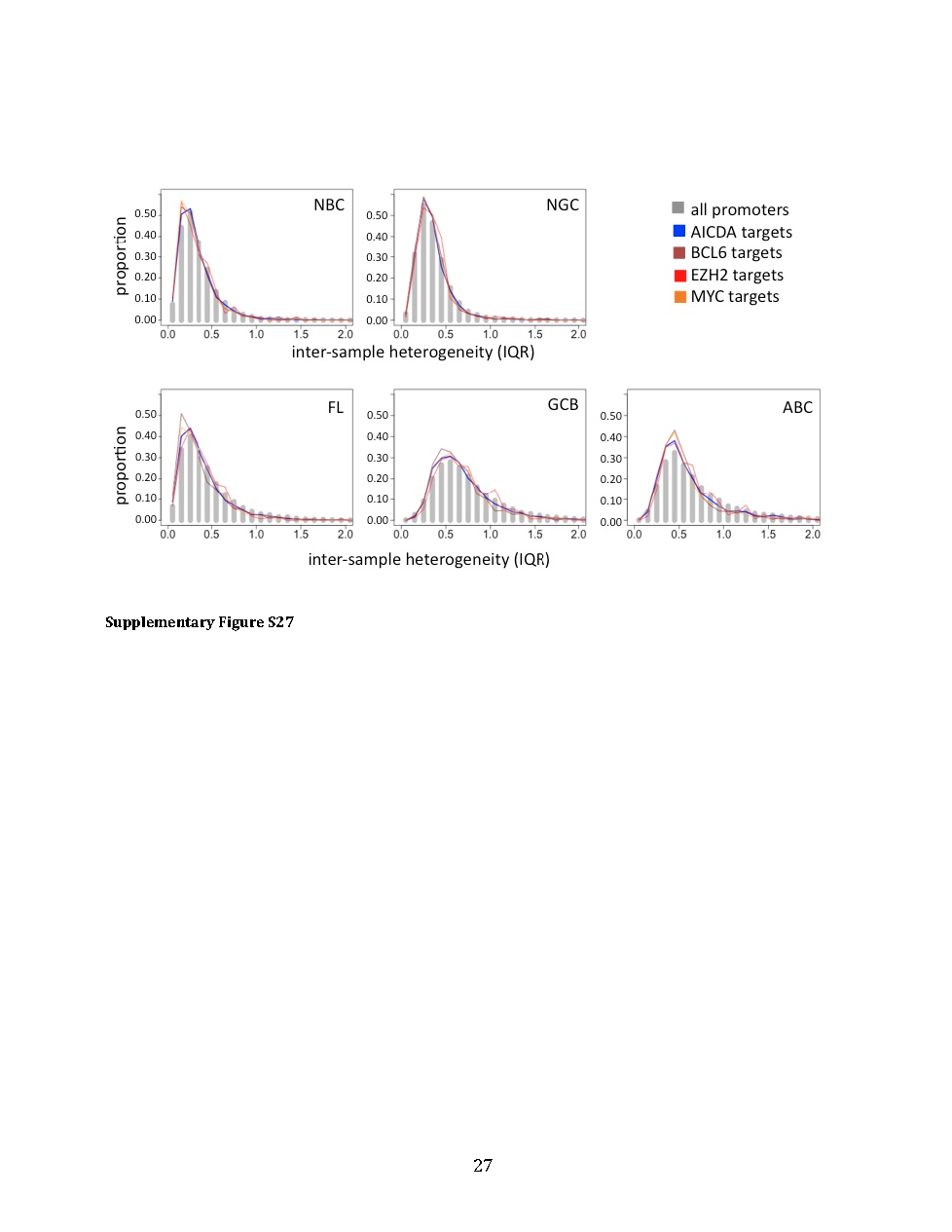

Supplement: Figure S27 — Inter-sample variation, measured using IQR, for the promoter methylation probe positions of the target genes of AICDA, BCL6, EZH2, and MYC and also all the promoter probe positions in our dataset. (TIF) [file pgen.1003137.s027.tif]

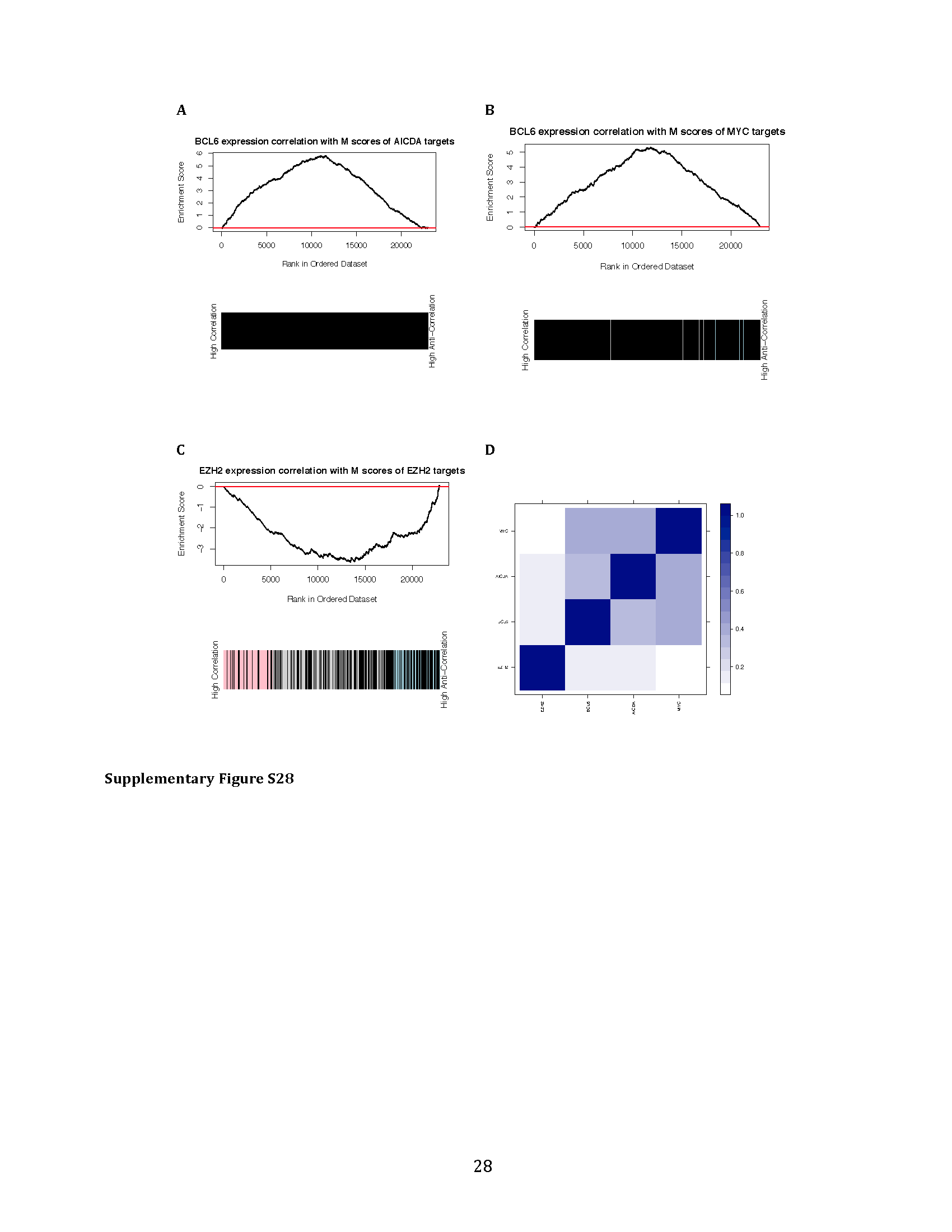

Supplement: Figure S28 — Association of promoter methylation of the target genes of AICDA, BCL6, EZH2, and MYC and transcription factor gene expression. This association was tested utilizing gene set analysis (GSA) and the plots (A–C) visualize the GSA results. The bar plots on the bottom visualize the TF targets in a ranking by correlation with gene expression, while the plots on the top visualize the local enrichment, i.e., the deviation from a random ranking. A positive enrichment score indicates that targets have a higher (positive) correlation of promoter methylation with expression than expected by chance. Promoter methylation of both AICDA and MYC is significantly associated with expression of BCL6 (panels A and B, respectively), while EZH2 expression is anti-correlated with its target promoter methylation (C). The highly similar results in A and B are due to high overlap of targets, i.e., because many genes are regulated by both MYC and AICDA (D). (TIF) [file pgen.1003137.s028.tif]

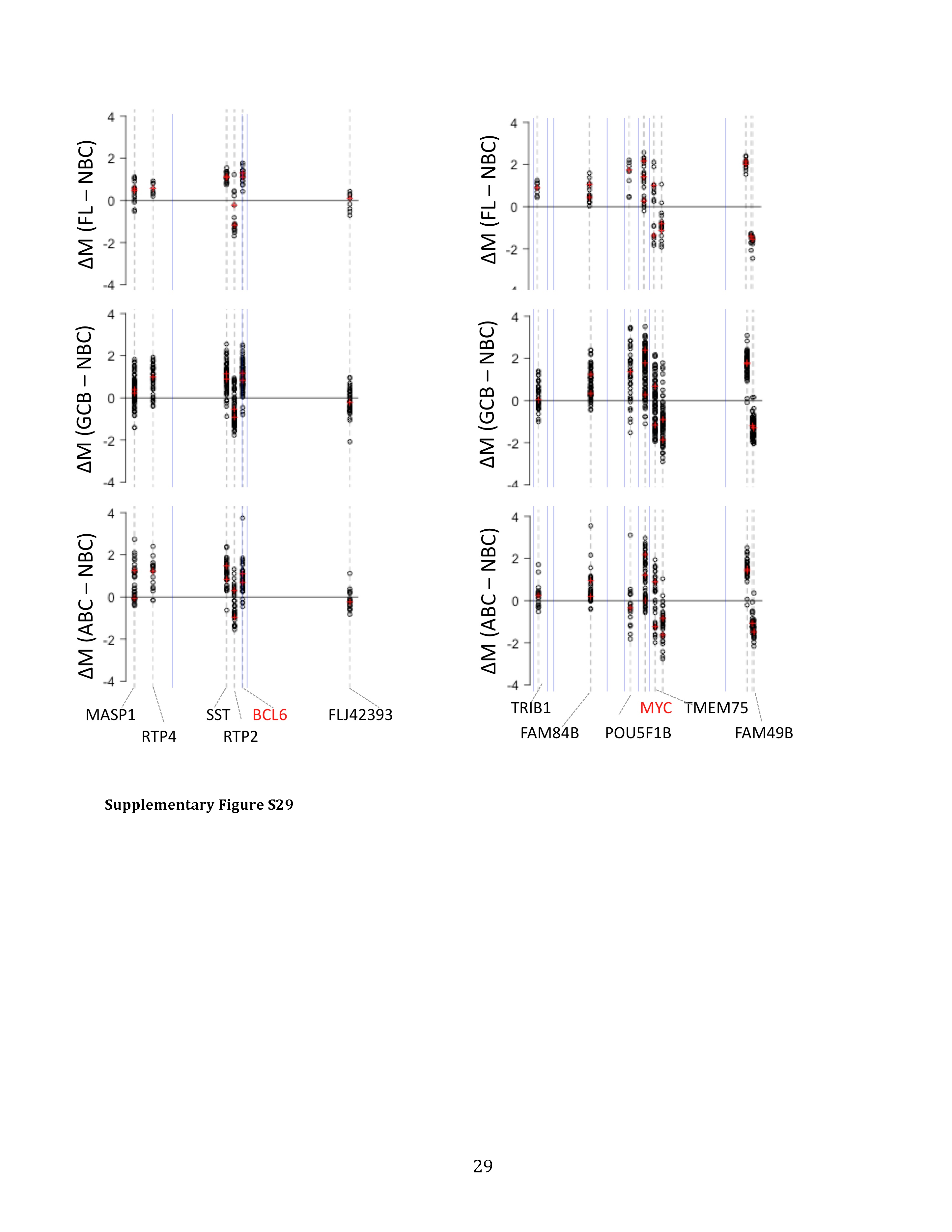

Supplement: Figure S29 — Extent of change in DNA methylation status of BCL6 (chr3:187 Mb) and MYC (chr8:128 Mb) loci, including the surrounding genes, in lymphoma samples (FL, GCB and ABC) compared to that in the normal NBC samples. Blue lines indicate CTCF binding sites. (TIF) [file pgen.1003137.s029.tif]

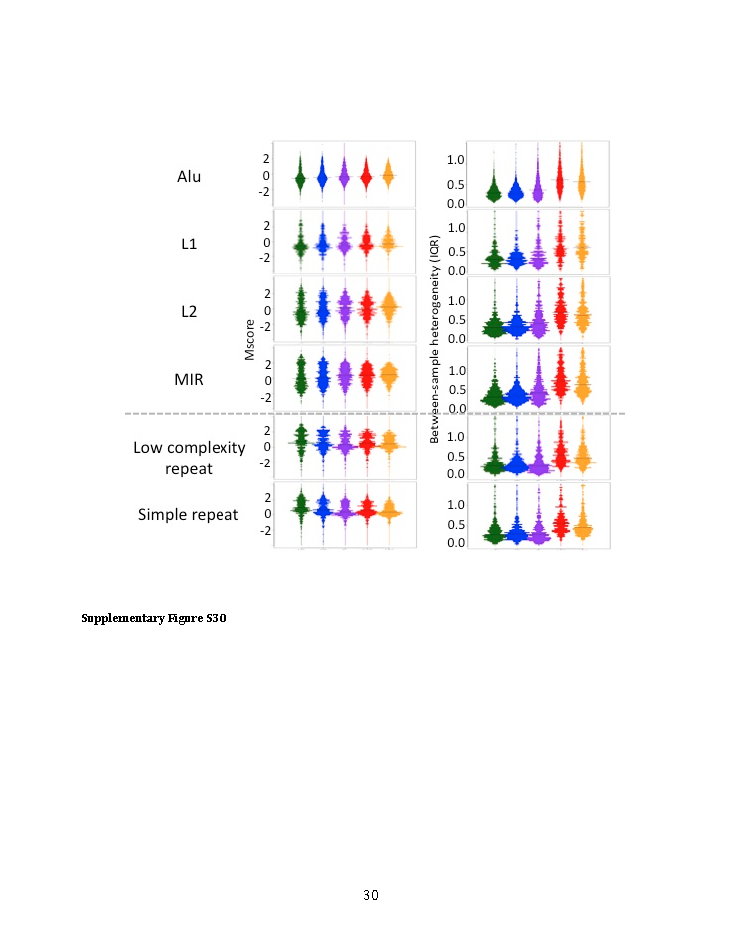

Supplement: Figure S30 — Distributions of M-score and inter-sample variation (IQR) for methylation probes that overlap with common repeat elements in normal and lymphoma samples. M-scores are shown on the left and IQR on the right. Color codes are the same as in Figure 1. Bar width is proportional to the number of probes with a given M-score as discussed in Figure 3A in the main text. The dotted horizontal line separates low complexity and simple repeats from other repeat classes. (TIF) [file pgen.1003137.s030.tif]
