# Supplementary material for: Aberration in DNA Methylation in B-Cell Lymphomas Has a Complex Origin and Increases with Disease Severity
Source: PLoS Genet. 2013 Jan 10;9(1):e1003137. doi: 10.1371/journal.pgen.1003137 (PMC3542081; doi:10.1371/journal.pgen.1003137)
Supplement: Table S1 — Patient characteristics. (PDF) [file pgen.1003137.s031.pdf]

Supplementary Table S1: Patient characteristics.

HELP ID	Diagnosis sample	Label	Age	Sex	PFS (months)	Progression
105788	DLBCL	ABC	54	F	97.182744	0
106067	DLBCL	GCB	47	M	28.221768	1
106086	DLBCL	GCB	57	M	7.1293632	1
108701	DLBCL	GCB	77	M		
114625	DLBCL	GCB	92	F	56.509248	0
114632	DLBCL	ABC	51	M	10.5462012	1
114982	DLBCL	GCB	80	F	38.340864	1
123576	DLBCL	GCB	53	M		
123578	DLBCL	GCB	80	F	82.989732	0
123586	DLBCL	GCB	62	M	1.7741268	1
124254	DLBCL	ABC	72	M	41.462016	1
124424	DLBCL	ABC	71	F	0.39425052	1
124574	DLBCL	GCB	57	F	63.61644	0
124761	DLBCL	GCB	22	M	49.544148	0
124790	DLBCL	GCB	50	M	55.129368	0
124801	DLBCL	GCB	62	M	68.533884	0
125068	DLBCL	ABC	84	M	55.78644	0
125125	DLBCL	GCB	79	M	48.952776	0
125126	DLBCL	ABC	57	M	5.5523616	1
125128	DLBCL	GCB	60	M	71.950716	0
125130	DLBCL	GCB	68	F	79.17864	0
125131	DLBCL	GCB	69	M	49.412736	0
125320	DLBCL	GCB	59	M	62.0616	0
125327	DLBCL	ABC	49	M	1.2813144	1
125408	DLBCL	GCB	56	M	64.701372	0
126481	DLBCL	GCB	19	M	65.741268	0
126485	DLBCL	ABC	81	M	8.6406564	1
126490	DLBCL	GCB	65	M	26.776176	1
128336	DLBCL	GCB	55	M	61.568796	0
128338	DLBCL	GCB	73	F		
130121	DLBCL	GCB	66	M	62.104104	0
130122	DLBCL	GCB	56	M	107.704104	0
145918	DLBCL	ABC	67	M	7.0308012	1
146863	DLBCL	ABC	57	F		
148170	DLBCL	GCB	61	M	52.632444	0
148673	DLBCL	ABC	50	M		
149297	DLBCL	GCB	68	M	34.201236	0
149365	DLBCL	GCB	80	F	49.67556	1
149366	DLBCL	ABC	60	M	44.451744	0
149371	DLBCL	GCB	68	F	47.277204	0
149413	DLBCL	GCB	49	F	45.470232	0
149435	DLBCL	GCB	76	M	27.498972	0
176549	DLBCL	GCB	76	M		
177615	DLBCL	ABC	54	M	53.420952	0
178307	DLBCL	ABC	69	F	44.747436	0
178421	DLBCL	ABC	66	M	48.45996	0
179880	DLBCL	ABC	45	M	38.078028	0
198121	DLBCL	ABC	73	M	33.774132	0

198130	DLBCL	GCB	58	M	34.529772	0
227453	DLBCL	ABC	67	M	41.65914	0
227457	DLBCL	GCB	78	M	43.594524	0
227460	DLBCL	GCB	58	M	35.646816	0
227461	DLBCL	GCB	67	M	36.960984	0
227526	DLBCL	GCB	77	M	39.983568	0
227528	DLBCL	GCB	68	M	36.566736	0
26361102	FL		84	F		
26364102	FL		53	M		
26365302	FL		83	M		
26371402	FL		48	M		
26375302	FL		52	M		
26394902	FL		60	F		
26405602	FL		59	F		
26407202	FL		69	M		
20198202	NBC					
20200602	NBC					
20200702	NBC					
20209402	NBC					
20216902	NBC					
129164	NBC					
129166	NBC					
130587	NBC					
20195402	NGC					
20200402	NGC					
20201702	NGC					
20202002	NGC					
20204302	NGC					
101693	NGC					
128811	NGC					
130062	NGC					
130502	NGC					
GSM435212_128081	CD34					
GSM435213_128500	CD34					
GSM435214_128503	CD34					
GSM435215_129639	CD34					
GSM435216_140452	CD34					
GSM435217_140456	CD34					
GSM435218_140457	CD34					
GSM435219_142831	CD34					
