# Supplementary material for: Aberration in DNA Methylation in B-Cell Lymphomas Has a Complex Origin and Increases with Disease Severity
Source: PLoS Genet. 2013 Jan 10;9(1):e1003137. doi: 10.1371/journal.pgen.1003137 (PMC3542081; doi:10.1371/journal.pgen.1003137)
Supplement: Table S2 — The doubling times of the cell lines used. (PDF) [file pgen.1003137.s032.pdf]

198130	DLBCL	GCB	58	M	34.529772	0
227453	DLBCL	ABC	67	M	41.65914	0
227457	DLBCL	GCB	78	M	43.594524	0
227460	DLBCL	GCB	58	M	35.646816	0
227461	DLBCL	GCB	67	M	36.960984	0
227526	DLBCL	GCB	77	M	39.983568	0
227528	DLBCL	GCB	68	M	36.566736	0
26361102	FL		84	F		
26364102	FL		53	M		
26365302	FL		83	M		
26371402	FL		48	M		
26375302	FL		52	M		
26394902	FL		60	F		
26405602	FL		59	F		
26407202	FL		69	M		
20198202	NBC					
20200602	NBC					
20200702	NBC					
20209402	NBC					
20216902	NBC					
129164	NBC					
129166	NBC					
130587	NBC					
20195402	NGC					
20200402	NGC					
20201702	NGC					
20202002	NGC					
20204302	NGC					
101693	NGC					
128811	NGC					
130062	NGC					
130502	NGC					
GSM435212_128081	CD34					
GSM435213_128500	CD34					
GSM435214_128503	CD34					
GSM435215_129639	CD34					
GSM435216_140452	CD34					
GSM435217_140456	CD34					
GSM435218_140457	CD34					
GSM435219_142831	CD34					
