# Supplementary material for: Aberration in DNA Methylation in B-Cell Lymphomas Has a Complex Origin and Increases with Disease Severity
Source: PLoS Genet. 2013 Jan 10;9(1):e1003137. doi: 10.1371/journal.pgen.1003137 (PMC3542081; doi:10.1371/journal.pgen.1003137)
Supplement: Table S3 — C-statistic with their standard errors (SE) and 95% confidence intervals of prognostic models in ABC and GCB samples. The C-statistic estimates the concordance of the predictions, which is the probability that in a random pair of non-censored patients, the one with higher risk relapsed earlier. (PDF) [file pgen.1003137.s033.pdf]

Supplementary Table S3: C-statistic with their standard errors (SE) and 95% confidence intervals of prognostic models in ABC and GCB samples. The C-statistic estimates the concordance of the predictions, which is the probability that in a random pair of non-censored patients, the one with higher risk relapsed earlier.

Covariates	C	SE	Lower 95	Upper 95
IPI (0-5)	0.642	0.094	0.457	0.826
Stage (1-4)	0.625	0.125	0.381	0.870
Methylation Heterogeneity Score (MHS)	0.579	0.092	0.377	0.781
IPI + MHS	0.701	0.096	0.514	0.889
Stage + MHS	0.678	0.131	0.420	0.935
