# Supplementary material for: Aberration in DNA Methylation in B-Cell Lymphomas Has a Complex Origin and Increases with Disease Severity
Source: PLoS Genet. 2013 Jan 10;9(1):e1003137. doi: 10.1371/journal.pgen.1003137 (PMC3542081; doi:10.1371/journal.pgen.1003137)
Supplement: Table S4 — C-statistics with their standard errors (SE) and 95% confidence intervals of prognostic models in ABC only. (PDF) [file pgen.1003137.s034.pdf]

**Supplementary Table S4: C-statistics with their standard errors (SE) and 95% confidence intervals of prognostic models in ABC only.**

| Covariates | C | SE | Lower 95 | Upper 95 |
|---|---|---|---|---|
| IPI (0-5) | 0.633 | 0.155 | 0.331 | 0.936 |
| Stage (1-4) | 0.680 | 0.158 | 0.369 | 0.990 |
| MHS | 0.688 | 0.122 | 0.449 | 0.927 |
| IPI + MHS | 0.842 | 0.109 | 0.628 | 1.000 |
| Stage + MHS | 0.872 | 0141 | 0.596 | 1.000 |
