# Supplementary material for: Aberration in DNA Methylation in B-Cell Lymphomas Has a Complex Origin and Increases with Disease Severity
Source: PLoS Genet. 2013 Jan 10;9(1):e1003137. doi: 10.1371/journal.pgen.1003137 (PMC3542081; doi:10.1371/journal.pgen.1003137)
Supplement: Text S1 — Supporting material. (DOC) [file pgen.1003137.s035.doc]

**Supplementary Module 1: Patient characteristics and control analyses**

**Patient Characteristics**

We obtained FL and DLBCL patient samples from the Vancouver Cancer Center in British Columbia, Canada. All FL and DLBCL samples used in the study were selected based on their high content of neoplastic cells from primary diagnostic material preceding treatment. **Supplementary Table S1** describes age and sex of the patients, their type of disease, and their corresponding identifiers in the HELP assay. Most of these patient samples were analyzed as part of previously published studies , which addressed separate biological questions.

**Copy number analysis**

To determine whether genomic alterations confounded our DNA methylation analyses, we collected SNP-chip-based copy number data for two GCB and two ABC samples. The copy number analysis was performed using the Affymetrix Genome-Wide Human SNP Array 6.0 platform (Affymetrix, Santa Clara, CA). Raw SNP data was processed using the Birdseed method included in Affymetrix Power Tools (APT, version 1.12.0). Affymetrix log2 intensity ratio data was considered with respect to the 210 HapMap reference models, which were also generated using the Affymetrix SNP 6.0 arrays. The Circular Binary Segmentation (CBS) method in the ‘DNACopy’ R package was used to call segmentations from the log ratio data on each array and the cutoff values of +0.15 and -0.15 were used to call gains and a losses, respectively (data not shown). GISTIC was used to identify significant copy number alterations across the DLBCL samples.

For each sample, we then overlaid the DNA methylation and copy number data. We first analyzed the M-score of those promoter methylation probesets that resided in copy number neutral, gain and loss regions; overall, we did not detect any systematic differences between neutral, gain and loss regions across all samples (**Supplementary Figure S1**); this was expected since the HELP assay uses the patients own DNA as the input channel for the assay.

These four samples represent only a subset of a larger patient cohort for which genotyping was performed. A set of genomic regions was flagged as frequently amplified or deleted in DLBCL using the GISTIC algorithm , and those were marked as GISTIC peaks. We separately analyzed the amplifications and deletions in the four samples that overlapped with the GISTIC peaks and the remaining genomic regions; again, no systematic difference was observed in the distributions of M-score across the four samples due to copy number changes (**Supplementary Figure S2**). Taken together, our analyses suggest that copy number alterations did not introduce a systematic bias into our analysis of DNA methylation variation.

**Sample purity analysis**

Information on sample purity was available for most of the lymphoma samples analyzed. In order to prove that greater IQR and intermediate M-score distributions were not the result of greater variation in sample purity in lymphoma samples, we selected primary cases in which the percentage of neoplastic cell purity was lower than in control samples (all control samples have a purity of > 90%, while some primary samples have a purity of < 90%) We used 2 FL samples, 12 GCB samples and 6 ABC samples with greater than 90% purity of neoplastic cells and estimated the distribution of M-score and IQR (**Supplementary Figure S3**), which recapitulated the patterns observed in **Figure 1C-D** in the main text. Therefore, sample purity is unlikely to introduce any major bias in our analysis.

**Exclusion of low signal-to-noise ratio probesets**

Certain methylation probesets have a low signal-to-noise ratio in one or more samples. We investigated the frequencies of these low intensity probes and also determined whether they introduced any systematic bias into our results. We found that less than 2% of probesets failed per sample; as previously discussed, probes with an intensity of < 2.5 mean absolute deviation (MAD) above the mean intensity of the random probes were considered failed. Overall, less than 4% of all probes on the array failed in one or more samples in our dataset. We then reanalyzed the distribution of M-scores after removing the low intensity probes (**Supplementary Figure S4**) and found that the M-score distribution was similar to the one reported in **Figure 1B-C**. Hence probeset quality did not introduce any systematic bias into our analysis.

**eRRBS validation analysis**

For six DLBCL samples, we also performed whole genome DNA methylation analysis using the Enhanced Reduced Representation Bisulfite Sequencing (eRRBS) approach . We used MspI to digest high molecular weight bisulfite-converted DNA. We then size-selected fragments of 70-320 bp length for the library preparation and performed sequencing on the Illumina HiSeq 2000 platform (Illumina Inc, San Diego, CA) using single-end 50 bp reads. eRRBS is a modification of RRBS that captures CpGs outside of CpG islands with 75% increase in coverage of CpG islands and 54% increase in coverage of CpG shores . eRRBS informs on the percent methylation of ~3 million CpGs within non-repetitive, CG-dense genomic regions corresponding preferentially to genes and their promoters. We used the whole-genome alignment approach provided by the Bowtie aligner in the Bismarck package . This analysis reports on the percentage of DNA methylation at any given CpG site in the genome. If multiple CpGs in a given HELP probe set were interrogated by the RRBS assay, we calculated the average percentage of RRBS DNA methylation for those probesets. Approximately 60% of the promoter methylation probesets in the HELP analysis had at least one CpG site that was also covered by the RRBS approach. For those probesets, we found that the extent of DNA methylation, as reported by the HELP assay (M-score), and the average percentage DNA methylation, as reported by the RRBS approach, were concordant (correlation coefficient > 0.4; **Supplementary Figure S5**). Note that a probe set in the HELP assay usually contains multiple CpG sites, and typically only a subset of them is interrogated using the RRBS assay. Selecting only those probe sets that are covered by multiple CpGs in the RRBS assay did not considerably improve the correlation coefficient (0.3-0.45 for > 3 CpGs; 0.25-0. 45 for > 5 CpGs; data not shown). The HELP assay does not report an absolute value for CpG methylation, but instead provides an intensity value that is proportional to the methylation status at those CCGG sites, which are rate-limiting for fragment generation. The relationship between the change in probe intensity and the change in methylation was identified based on the technical validation of the array and was found to be linear (**Supplementary Figure S5**). A comparison of the array data to RRBS data is complicated by different design principles of the assays: the microarray assay assesses the extent of methylation indirectly while the RRBS approach assesses it directly. Nevertheless, we observed a correlation between these two approaches, which supports our findings.

**Methylation distribution based on eRRBS**

Next, we studied genome-wide methylation patterns in 4 NGC and 6 DLBCL samples using the eRRBS assay. At a genome-wide scale, there were ~3 million CpGs that were interrogated by the eRRBS assay. On average, most of the CpGs had either close to 100% or close to 0% methylation, and very few had intermediate values. These intermediate values indicate intra-sample heterogeneity. We further analyzed intermediate values of 30-70%, finding that 10-12% of the CpGs in the normal centroblast samples belonged to this category, while 14-17% of CpGs in the DLBCL samples were in this category. This difference between normal and lymphoma samples was significant (binomial test: p-value < 1x10^-5^). Our finding was independent of the cut-off for intermediate methylation values chosen. For instance, when choosing cut-offs of 20-80%, then 20-22% of CpGs in the NGB samples belong to this category, while 21-27% of CpGs in the DLBCL samples belong to this category. When choosing cut-offs of 40-60%, then 5.2-6.6% of CpGs in the NGB samples belong to this category, while 7.6-10% of CpGs in the DLBCL samples belong to this category. Again, this difference between normal and lymphoma samples was significant (binomial test: p-value < 1x10^-5^)

**MassARRAY validation analysis**

We then used the Sequenom MassARRAY EpiTYPER approach to validate our HELP findings of increasing M-scores in lymphoma samples as compared to normal B-cells. The MassARRAY remains the gold standard, since it is not based on enzymatic digestion of DNA, but on bisulfite conversion of methylated proves followed by PCR amplification of the DNA target of interest; finally, mass spectrometric detection analysis is used, which allows the determination of the percentage of methylated cytosines in each specific genomic position. We selected 4 random genes and studied their methylation in 4 NGC samples and multiple DLBCL samples (**Supplementary Figure S6**). The number of targets and cases were dictated by limited genomic material available.

We identified that all except 4 CpGs in all 4 genes had greater variance in DLBCL samples as compared to NGC samples, using the robust Brown-Forsythe Levene test based on the absolute deviations from the median using the levene.test() function in R. The majority of CpGs revealed a greater than 3 times higher variance in DLBCLs with q-values < 0.05 (**Supplementary Figure S7**). In this figure, rows represent individual CpGs in the amplicon studied, while columns correspond to individual samples, as labeled below the heatmap.

In addition to biological validations, we also performed technical validations of our HELP findings using the Sequenom MassARRAY by interrogating multiple random genes in the DLBCL samples. Technical validation allows a correlation of the array signal. We used quantile normalized log2(HpaII/MspI) values with a percent methylation calculated for the HpaII amplifiable fragment based on absolute methylation values at critical CCGG sites based on Mass Array (**Supplementary Figure S8**). We detected a linear correlation between the signal obtained from the HELP assay and the percentage of methylation based on the MassARRAY; the difference of log2(HpaII/MspI) of one was equal to an approximately 30% change in methylation.

**Chromosome-wide patterns of DNA methylation in normal and lymphoma samples**

We then overlaid the promoter methylation probe sets along the human chromosomes (**Supplementary Figure S9**) and found that in general, the sites of hypo- and hyper-methylation were distributed across all chromosomes in both normal and lymphoma samples.

**Alternative measures for inter-sample variation**

In addition to IQR, at each probe set, we also computed the inter-sample standard deviation of the M-score. We found that the inter-sample standard deviation increases from normal lymphoid cell types (NBC and NGC) to lymphoma subtypes (FL, GCB and ABC; **Supplementary Figure S10**). For instance, approximately 90% of the probe sets had a greater standard deviation of the M-score in the ABC group compared to that in the NGC group. The difference in variance between the groups was modest (p-value > 0.05) for a majority of the cases (Brown-Forsythe Levene-type test) after adjusting for multiple testing by the FDR method; this lack of significance was probably due to the small sample sizes.

**Mitotic rate analysis**

Cells in the S phase of the cell cycle may have a higher proportion of hemi-methylated CpG sites than those in other phases of the cell cycle. To investigate whether the cell cycle phase and mitotic rate have any impact on our findings, we collected nine cell lines, which were grouped according to low, intermediate and high doubling time (**Supplementary Table S2**), and compared the distributions of their M-scores (**Supplementary Figure S11**). DLBCL cell lines were grown in the exponential phase and their doubling time (DT) was calculated as follows:

DT = (*t* - *t*_0_) log2 / (log*N -*log*N*_0_), where *t* and *t*_0_ are the times at which the cells were counted, and *N* and *N*_0_ are the cell numbers at times *t* and *t*_0_.

We found that there was no systematic difference in the M-score between the groups, i.e. (i) the variation in the median M-score between the groups was not significantly greater than that within groups (p-value > 0.05; ANOVA), and (ii) the variation in the inter-quartile range (Q3-Q1) of the M-score between the groups was not significantly greater than that within groups (p-value > 0.05; ANOVA). In light of these findings, we believe that the mitotic rate has no significant impact on the M-score distribution of the normal and lymphoma samples, and that our observations are unlikely to be due to differences in mitotic rates. We also acknowledge that cell lines are different from primary lymphoma cells, and thus further work needs to be done to firmly exclude this possibility.

**Distribution of CpG methylation in age-matched controls**

We utilized three peripheral blood B-cell controls ages 20-30 as “young” controls; three peripheral blood B-cell controls ages 60-70 as “old” controls; and 10 DLBCLs with an average age of 65.3 years. All cases were profiled using eRRBS and all methylation values are shown in **Supplementary Figure S12**.

As can be seen from the diagram, there are small differences in methylation rates in the young vs old controls with slightly higher extreme methylation values in the young controls. The differences between controls based on age are small and both controls have significantly different profiles from DLBCLs (p < 0.05, ANOVA). Thus, young and old controls are much more similar to each other than to DLBCLs. This data suggests that differences between controls and DLBCLs cannot be explained strictly by the age differences in cohorts.

**Supplementary Module 2: Methylation patterns at CpG island and non-CpG island positions**

We identified the position of CpG islands from the UCSC Genome Browser . We classified probesets according to whether they were associated with CpG islands or not, and found that probesets within CpG islands were hypomethylated in normal cells, while those outside CpG islands were predominantly hypermethylated (**Supplementary Figure S13)**. CpG island probesets gained methylation in the lymphoma samples. While CpG islands displayed a bimodal methylation pattern in normal samples, this distribution was more heterogeneous in GCB and ABC samples. In contrast, non-CpG island probesets lost methylation gradually with increasing disease severity, i.e. NBC<NGC<FL<GCB<ABC, and there was weak bimodal pattern in normal and lymphoma tissues.

**Effects of CpG density**

To investigate whether the CpG density in the promoter regions of genes had any effect on our analyses, we plotted the distribution of the M-score against the number of CpG sites in the corresponding promoters for the NBC, NGC, FL, GCB and ABC samples (**Supplementary Figure S14)**. We found that typically, promoters with high CpG density tended to have a relatively high M-score, indicating more hypomethylation. To investigate whether this was a biological phenomenon or a technical artifact, we plotted the distribution of the percentage promoter methylation against CpG density at those same promoters (**Supplementary Figure S15)** and found consistent results – indicating that this trend is unlikely to arise from technical artifacts of the HELP assay.

In addition, we plotted the distribution of |M-score| (i.e. absolute value of M-score) against the CpG density in the corresponding promoters for the NBC, NGC, FL, GCB and ABC samples (**Supplementary Figure S16)**. We obtained no significant association, indicating that the CpG density is uncorrelated with the magnitude of the log2 ratios in the methylation microarrays. We also plotted the distribution of IQR values, which reflect between-sample variation, against CpG density in the corresponding promoters for the NBC, NGC, FL, GCB and ABC samples (**Supplementary Figure S16)**. Again, we found no significant association, indicating that the CpG density is uncorrelated with the between-sample variation in the methylation microarrays.

**DNA methylation patterns in centromeric and telomeric regions**

We then estimated DNA methylation patterns in the centromeric, telomeric and intermediate regions using eRRBS data (**Supplementary Figure S17)**. We found that, even though in the eRRBS assay, most of the CpG sites had close to 0% or close to 100% methylation, the results were consistent with those reported in **Figure 3B**.

**Supplementary Module 3: Survival analysis and phylogenetic tree analysis**

As described in the **Methods** and **Figure 1F,** we built a phylogenetic tree of normal and lymphoma samples using promoter methylation data. Here, we further tested whether the extent of aberrant promoter methylation in lymphoma samples has prognostic information. For 33 GCB and 16 ABC samples, follow-up data (median 3.72 years, range 0.03-8.97), the International Prognostic Index (IPI) , and cancer stage information was available . We built multivariate Cox proportional hazards models with the average correlation distance to NGC (taken from the distance matrices calculated as described in the Methods section) and also IPI or stage (binned into I, II, III and IV) as covariates. This correlation distance represents a methylation heterogeneity score (MHS), reflecting how different the methylation profile is from normal B cells or NGC. The right endpoint was set to be relapse-free survival. The Cox model was then used to estimate the risk of relapse (i.e. risk = b_1_ IPI + b_2_ MHS). The MHS score was significantly associated with overall survival in the ABC samples, both adjusted (p-value = 0.012) and unadjusted for IPI (p-value = 0.016). It was not significant in GCB samples, potentially due to the small number of events (adjusted p-value = 0.38).

We then utilized permutation tests to estimate how well our findings might generalize to independent cohorts. Due to the lack of an independent validation set, we used leave-one-out cross-validation to predict these risk scores for each patient, i.e., for every patient, a risk score was calculated by a Cox proportional hazards model that was fitted using the methylation data from the remaining patient samples only. Patients were then classified as high- and low-risk depending on whether their risk scores were above or below the median cross-validated risk score. Analyzing the GCB and ABC samples together, we found that the distance measure from NGC is a predictor of survival (**Figure 2b**). We observed similar trends when GCB or ABC samples were analyzed independently, but it was again not significant in the GCB samples. The significance of the survival curve differences was estimated with a permutation test. The process from cross-validation to risk stratification was repeated 500 times with shuffled survival labels. This resulting empirical chi-square distribution was then utilized to estimate p-values. To evaluate a risk stratification based on the MHS alone, the high- and low-risk groups were again assigned based on the cohort median. For analyses using stage alone, we compared stage I, II versus III, IV. Based on IPI, patients were classified as high-risk with IPI greater than 1, otherwise as low-risk. The log-rank test was used to estimate p-values for these univariate models. In addition to **Figure 2b**, where we compare IPI and MHS, we show in **Supplementary Figure S18** a comparison of stage and MHS with very similar results.

We further analyzed the C-statistic (C) of the risk scores, which estimates the concordance of the predictions. The R package survC1 was used for this analysis. The concordance is the probability that in a random pair of non-censored patients, the one with higher risk relapsed earlier. **Supplementary Table S3** lists all Cs from all analyzed models and in both ABC and GCB.

The best model was IPI and MHS together in a multivariate model, with an increase in C of 0.059 (95% CI -0.07 -0.20) compared to IPI alone. MHS alone showed higher concordance in the first two years (0.76, 95% CI 0.51-1.00) than IPI alone (0.519, 95% CI 0.38-0.93) or than the combined model (0.65, 95% CI 0.38-0.93). Patients who died early thus showed a high extent of aberrant promoter methylation.

We then repeated the same analysis for the subset of ABC patients only and show the results in **Supplementary Table S4** and **Supplementary Figure S19**. The combined model IPI+MHS had again the highest concordance of all models. The increase in concordance compared to IPI alone was 0.21 (95% CI -0.06 - 0.47). The combined model identified all relapses correctly, yielding an infinitive Hazard Ratio (**Supplementary Figure S17e**).

**Follicular lymphoma grade analysis**

We collected grade information for the follicular lymphoma samples and analyzed their methylation patterns in the context of grade information. There were three samples of grade 1, two samples of grade 2 and three samples of grade 3. The grade of sample # 26405602 was classified as 1 or 2. We calculated the M-score distribution of the follicular lymphoma samples grouped according to grade and found that the M-score distribution of the grade 1 samples was predominantly bimodal while bimodality was not apparent in grade 2 and 3 samples (**Supplementary Figure S20**). The difference was statistically significant (p-value < 1x10^-5^; Kolmogorov Smirnov test). Our findings suggest that for follicular lymphoma, DNA methylation patterns become increasingly heterogeneous with an increase in disease severity.

**Supplementary Module 4: Promoter DNA methylation analysis based on different gene densities**

We divided the genome into non-overlapping blocks with 100kb resolution and counted the number of unique Ensembl v59 genes in each block. We flagged a genomic block as gene-poor, intermediate, or gene-rich, depending on whether there was no gene, there were 1- 3 genes, or ≥ 4 genes in that block; this segmentation was similar to the quantile distribution of gene density in the genome. In all normal (NBC and NGC) and lymphoma (FL, GCB and ABC) samples, the gene-poor blocks were significantly hyper-methylated compared to gene-rich blocks (p-value: <1x10^-15^, Mann Whitney test). In the NBC and NGC samples, the inter-sample variability, estimated by IQR, was not significantly different between gene-poor and gene-rich blocks (p-value > 1x10^-3^; Mann Whitney test), but the GCB and ABC samples showed a significant difference (p-value < 1x10^-10^; Mann Whitney test).

Next, we excluded centromeric and telomeric regions and then analyzed the distribution of promoter M-scores for normal and lymphoma tissues for 100kb non-overlapping blocks of low, intermediate and high gene densities (**Supplementary Figure S21**). We found that the pattern of promoter methylation was similar to that displayed in Figure 2D.

**Supplementary Module 5: Spreading of promoter methylation in lymphoma samples**

To investigate if aberrant methylation patterns spread in the genomic neighborhood (**Figure 4A-B**), we first identified the probesets that displayed significantly aberrant methylation in lymphoma samples, and then focused on their neighborhoods.

As a first step, at each methylation probe position, we compared the distribution of M-scores between NGC B-cells and lymphoma samples (FL, GCB and ABC combined) using the Mann-Whitney test, and then selected promoter methylation probesets that were significantly differently methylated using a FDR corrected p-value cut-off of 5.0x10^-3^. There were 5,688 probes that satisfied these criteria. These probes were distributed across all chromosomes; only two pairs of probe positions that suffered aberrant hyper-methylation and one pair of probe positions that suffered aberrant hypo-methylation had a distance of less than 100kb on the linear chromosomes between the pair(s). To avoid any bias due to effects of gene-desert regions, we excluded those cases in which there were fewer than 2 and 5 neighboring promoter methylation probe positions within 50 and 100kb, respectively. There were 5,458 probesets that passed this filter. Of them, 2,044 and 3,144 displayed aberrant hypo- and hyper-methylation, respectively, in lymphoma samples. For the promoter probes that suffered aberrant hypo- and hyper-methylation, the differences in M-score between NGC and ABC samples were on average -1.09 and 1.69, respectively.

We then considered the cases with aberrant hypo-methylation in DLBCLs, and denoted those probe positions as “i”. We scanned up- and downstream regions and denoted neighboring promoter probe positions as “i±1”, “i±2”, … “i±5”, such that “i±1” referred to the immediate neighbors of “i”, and “i±5” referred to the promoters which were located 5 promoter methylation probe positions away from position “i”. We calculated the difference in M-score between NGC and ABC (**Figure 4B**), NGC and GCB (**Supplementary Figure S22a**), and NGC and FL samples (**Supplementary Figure S22b**) at “i”, “i±1”, … “i±5” positions. We also calculated the difference in IQR between the NGC and ABC (**Figure 4B**), NGC and GCB (**Supplementary Figure S22c**), and NGC and FL (**Supplementary Figure S22d**) samples at “i”, “i±1”, … “i±5” positions. We observed that in addition to “i”, its neighboring promoter methylation probe positions underwent aberrant hypo-methylation although the effect weakened with distance. We repeated the analysis for the set of methylation probes that suffered aberrant hyper-methylation in DLBCLs and found similar results – neighboring promoter probe positions underwent aberrant hypo-methylation although the effect weakened with distance.

Some of the genes may have multiple promoter methylation probesets, and some promoter probes may map to multiple genes. Therefore, we repeated our analysis after excluding these cases, such that the neighboring probesets marked distinct genes (**Supplementary Figure S23**). We denoted these probesets by “g”, “g±1” … “g±5”, to distinguish them from those in **Supplementary Figure S22**. We observed similar neighborhood effects – when position “g” suffered significant hypo- or hyper-methylation, the neighboring gene promoters also showed similar effects that weaken over distance, and the neighborhood effect was more widespread in case of aberrant hypo-methylation compared to aberrant hyper-methylation.

**Supplementary Module 6: CTCF-binding site (BS) analysis**

We excluded centromeric and telomeric regions and then analyzed the distribution of promoter M-scores for normal and lymphoma tissues for 100kb non-overlapping blocks of low, intermediate and high CTCF-BS densities (**Supplementary Figure S24**). We found that the pattern of promoter methylation was similar to that displayed in **Figure 4C**.

We then investigated the neighborhoods of differentially methylated promoters by overlaying CTCF-BS data. We divided the methylation probes into two groups: (i) those whose neighboring promoter probes were separated by at least one CTCF-BS, and (ii) those whose neighboring promoter probes were not separated by any CTCF-BS. Focusing on the promoters that suffered aberrant hypo- and hyper-methylation in FL and GCB samples (**Supplementary Figure S25**), we obtained results similar to those observed for the ABC samples (**Figure 5B**) - promoter pairs not containing intervening CTCF binding sites displayed greater spreading of aberrant methylation from one promoter to the other, compared to those that had one or more intervening CTCF binding sites. This effect was stronger for gain of aberrant hypo-methylation compared to gain of aberrant hyper-methylation in lymphoma samples.

**Validation of CTCF-binding site (BS) analysis using the RRBS assay**

We repeated the analysis, as described in **Figure 5B** and **Supplementary Figure S25**, using RRBS data. In brief, we collected genome-wide methylation patterns in 4 normal NGC and 6 DLBCL samples using the RRBS assay. We first estimated the promoter DNA methylation patterns using the RRBS assay, and then divided the promoters into two groups: (i) those whose neighboring promoters were separated by at least one CTCF-BS, and (ii) those whose neighboring promoters were not separated by any CTCF-BS. Focusing on the promoters that suffered aberrant hypo- and hyper-methylation in lymphoma samples (**Supplementary Figure S26**), we obtained results similar to those observed for the ABC samples (**Figure 5B**) - promoter pairs not containing intervening CTCF-BS displayed greater spreading of aberrant methylation from one promoter to the other, compared to those that had one or more intervening CTCF-BS. This effect was evident for gain of aberrant hypo-methylation, but not in the case of aberrant hyper-methylation in lymphoma samples – which is consistent with our findings reported in **Figure 5B** using the HELP assay.

**Supplementary Module 7: Effects of modifier genes on genome-wide methylation changes**

**Changes in promoter methylation status and expression of the same gene are correlated**

We obtained both genome-wide promoter methylation and gene expression data for 4 NBC and 45 DLBCL samples (13 ABC and 32 GCB). Expression data for NBC were obtained from GSE15271, generated using HG133_Plus2_microarray and mas5 normalized together with the expression data for the DLBCL samples (GSE23501). For each gene in each DLBCL sample, we calculated the extent of change in promoter methylation (ΔM) and log2 gene expression (Δex) relative to the median value of these parameters in the normal centroblasts. By interrogating the genes that have high variance (top 25%) in both expression (Δex) and methylation (ΔM), we found that the two parameters were significantly correlated (correlation coefficient: 0.03, p-value < 1x10^-3^; permutation test). We repeated the analysis using a different variance cut-off (top 50%) in both expression (Δex) and methylation (ΔM), and found a consistent significant positive correlation (correlation coefficient: 0.02, p-value < 1x10^-3^; permutation test). In addition to promoter methylation, transcription factor binding, copy number status and other genomic features influence gene expression, and therefore the moderate correlation is not unexpected. Nevertheless, loss of methylation at gene promoters was associated with an increase in expression of those genes.

**Analysis of target genes of AICDA, BCL6, EZH2 and MYC**

We also computed inter-sample variation, measured using IQR, for the promoter methylation probe positions of the target genes of AICDA, BCL6, EZH2 and MYC. We found that the distribution of IQR is similar to that of all promoter probe positions in our dataset (**Supplementary Figure S27**).

We then tested whether the expression of AICDA, BCL6, EZH2, and MYC directly influences the promoter methylation of their targets. For that purpose, we calculated the Pearson correlation of expression of all 4 transcription factors (TFs) with ΔM of their targets (the methylation aberration compared to normal centroblasts as described above. All promotor ΔM scores were ranked by Pearson correlation with TF expression from “highly correlated” to “highly anti-correlated”. Utilizing the gene set analysis (GSA) from the limma package (wilcoxGST function), we then tested for non-random ranking of targets, i.e., whether the TF expression is significantly associated with the TF target promoter methylation. We found that BCL6 expression was highly correlated with promoter methylation of MYC and AICDA targets, EZH2 was highly anti-correlated with ΔM scores of EZH2 targets (FDR < 0.001, **Supplementary Figure S28**).

**Methylation status of BCL6 and MYC loci**

MYC and BCL6 are key genes implicated in DLBCL. Here we describe the extent of change in the DNA methylation status of the BCL6 and MYC loci, including the surrounding genes, in lymphoma samples (FL, GCB and ABC) compared to that in the NBC samples. We found that both BCL6 and MYC experienced loss of promoter methylation in lymphoma samples compared to normal samples. We also observed that the neighboring genes often, but not always, showed alteration in DNA methylation in the same direction (**Supplementary Figure S29**).

**Genome-wide analysis**

Using the matched mRNA expression and genome-wide promoter methylation data from the 45 DLBCL samples, we tested a set of 11 genes that are implicated in DLBCL pathogenesis for association between gene expression and global methylation changes. We further identified the top genes associated with global effects on promoter methylation. We performed two independent analyses to investigate the association of gene expression with aberrant DNA methylation patterns. **Figure 7** summarizes two independent analyses:

1. In our first analysis, we focused on the top 25% (5,734) of all promoters with the highest variance. First, we calculated, for each gene, the correlation of expression with ΔM of the 5,734 promoters with the largest variance. This approach resulted in 5,734 Pearson correlation coefficients per gene, which are visualized with box plots in **Figure 7a**. A box plot with median below 0, for example, indicates an anti-correlation of expression with ΔM. This means that an up-regulation of the gene is associated with hypermethylation of the top-variance promoters. Including low-variance promoters would lower the average Pearson correlation coefficient for all genes. **Figure 7a** shows these correlation coefficients for 11 key genes implicated in DLBCL pathogenesis. The choice of these 11 genes was guided by our prior knowledge of their role in lymphomagenesis, epigenetic regulation and hematopoietic development .
2. In the second approach, we used a robust published approach that is independent of parameters and that summarizes the correlation coefficients of all promoters in one single quantity, R^2^ . This quantity is an estimate of how much a particular gene’s expression could maximally explain the global methylation changes. These numbers are written on top of the box plots in **Figure 7**. We repeated exactly the same analysis genome-wide for all genes to identify genes whose expression has highest association with methylation changes (**Figure 7b**). The gene expression of our top hit in **Figure 7b**, WHSC1L1, is a known epigenetic modifier and can explain 9.2% of the total variance in global promoter methylation. Genes with a significant R^2^ value (p-value < 0.05) are marked with an asterisk. We calculated the p-values with a permutation test, in which the analysis was repeated 10,000 times with randomized promoter methylation, i.e., in which the ΔM scores for all promoters were shuffled independently. The R^2^ estimate indirectly weighs promoter correlation coefficients by variance, and is thus independent of parameters such as variance filtering; see for details. We therefore used all 22,937 assayed promoters in this analysis.

**Supplementary Module 8: Analysis of repeat elements**

We obtained information on repeat elements from the UCSC Genome Browser. Gain of de novo methylation and maintenance of methylated states over generations of cell divisions are mediated by several DNA methyl-transferase family genes. Among them is the DNMT3 (A, B, L) class of DNA methyl-transferases, which play a key role in de novo methylation; this process, however, appears to be specific to selective repeat classes . Furthermore, some repeat classes preferentially occur within certain chromosomal regions (e.g. centromeres preferentially contain simple repeats). To assess whether specific repeat classes are targeted for abnormal methylation in lymphoma samples, we overlaid repeat sequence data with the methylome data and classified the MSP probes that overlap with the most common repeat classes, i.e. those which are present with at least 350,000 copies in the genome.

We found that distinct methylation patterns were associated with various repeat classes (**Supplementary Figure S30**). Alu and L1, which are active in the germ line , were predominantly hyper-methylated in normal as well as lymphoma samples; these repeat classes displayed a limited loss of methylation with increasing disease aggressiveness, and moderate inter-sample variability. L2 and MIR repeats exhibited a bimodal methylation pattern in normal tissue and interestingly, that bimodality is preserved, albeit weakly, in lymphoma samples. Inter-sample variation was comparatively higher in this repeat class. Finally, low complexity repeats and simple repeats displayed hypo-methylation in normal tissues, but increased hyper-methylation in lymphoma samples, and also overall slightly lower inter-sample variability among the repeat classes. Of note, DNMT3 targets of de novo methylation, e.g. D4Z4 repeats , fall into these repeat classes. Thus, DNMT3 genes may have key roles in the aberrant gain of methylation in the lymphomas.

**Supplementary References**

1. Shaknovich R, Geng H, Johnson NA, Tsikitas L, Cerchietti L, et al. (2010) DNA methylation signatures define molecular subtypes of diffuse large B-cell lymphoma. Blood 116: e81-89.

2. Olshen AB, Venkatraman ES, Lucito R, Wigler M (2004) Circular binary segmentation for the analysis of array-based DNA copy number data. Biostatistics 5: 557-572.

3. Venkatraman ES, Olshen AB (2007) A faster circular binary segmentation algorithm for the analysis of array CGH data. Bioinformatics 23: 657-663.

4. Beroukhim R, Getz G, Nghiemphu L, Barretina J, Hsueh T, et al. (2007) Assessing the significance of chromosomal aberrations in cancer: methodology and application to glioma. Proc Natl Acad Sci U S A 104: 20007-20012.

5. Meissner A, Gnirke A, Bell GW, Ramsahoye B, Lander ES, et al. (2005) Reduced representation bisulfite sequencing for comparative high-resolution DNA methylation analysis. Nucleic Acids Res 33: 5868-5877.

6. Akalin AK, E. G-BF, Kormaksson M, Busuttil J, Zhang L, et al. (2012) Base-pair resolution DNA methylation sequencing reveals profoundly divergent epigenetic landscapes in Acute Myeloid Leukemia. PLoS Genetics.

7. Krueger F, Andrews SR (2011) Bismark: a flexible aligner and methylation caller for Bisulfite-Seq applications. Bioinformatics 27: 1571-1572.

8. Fujita PA, Rhead B, Zweig AS, Hinrichs AS, Karolchik D, et al. (2011) The UCSC Genome Browser database: update 2011. Nucleic Acids Res 39: D876-882.

9. TIN-HLPFP (1993) A predictive model for aggressive non-Hodgkin's lymphoma. The International Non-Hodgkin's Lymphoma Prognostic Factors Project. New England Journal of Medicine 329: 987-994.

10. Uno H, Cai T, Pencina MJ, D’Agostino RB, Wei LJ (2011) On the C-statistics for evaluating overall adequacy of risk prediction procedures with censored survival data. . Statistics in Medicine 30: 1105-1116.

11. Flicek P, Amode MR, Barrell D, Beal K, Brent S, et al. (2011) Ensembl 2011. Nucleic Acids Res 39: D800-806.

12. Ci W, Polo JM, Cerchietti L, Shaknovich R, Wang L, et al. (2009) The BCL6 transcriptional program features repression of multiple oncogenes in primary B cells and is deregulated in DLBCL. Blood 113: 5536-5548.

13. Rimsza LM, Leblanc ML, Unger JM, Miller TP, Grogan TM, et al. (2008) Gene expression predicts overall survival in paraffin-embedded tissues of diffuse large B-cell lymphoma treated with R-CHOP. Blood 112: 3425-3433.

14. Skinnider BF, Horsman DE, Dupuis B, Gascoyne RD (1999) Bcl-6 and Bcl-2 protein expression in diffuse large B-cell lymphoma and follicular lymphoma: correlation with 3q27 and 18q21 chromosomal abnormalities. Hum Pathol 30: 803-808.

15. Cattoretti G, Pasqualucci L, Ballon G, Tam W, Nandula SV, et al. (2005) Deregulated BCL6 expression recapitulates the pathogenesis of human diffuse large B cell lymphomas in mice. Cancer Cell 7: 445-455.

16. Ci W, Polo JM, Melnick A (2008) B-cell lymphoma 6 and the molecular pathogenesis of diffuse large B-cell lymphoma. Curr Opin Hematol 15: 381-390.

17. Fritz EL, Papavasiliou FN (2010) Cytidine deaminases: AIDing DNA demethylation? Genes Dev 24: 2107-2114.

18. Liu M, Schatz DG (2009) Balancing AID and DNA repair during somatic hypermutation. Trends Immunol 30: 173-181.

19. Millar CB, Guy J, Sansom OJ, Selfridge J, MacDougall E, et al. (2002) Enhanced CpG mutability and tumorigenesis in MBD4-deficient mice. Science 297: 403-405.

20. Morin RD, Johnson NA, Severson TM, Mungall AJ, An J, et al. (2010) Somatic mutations altering EZH2 (Tyr641) in follicular and diffuse large B-cell lymphomas of germinal-center origin. Nat Genet 42: 181-185.

21. Pasqualucci L, Bhagat G, Jankovic M, Compagno M, Smith P, et al. (2008) AID is required for germinal center-derived lymphomagenesis. Nat Genet 40: 108-112.

22. Shah MY, Vasanthakumar A, Barnes NY, Figueroa ME, Kamp A, et al. (2010) DNMT3B7, a truncated DNMT3B isoform expressed in human tumors, disrupts embryonic development and accelerates lymphomagenesis. Cancer Res 70: 5840-5850.

23. Shaknovich R, Cerchietti L, Tsikitas L, Kormaksson M, De S, et al. (2011) DNA methyltransferase 1 and DNA methylation patterning contribute to germinal center B-cell differentiation. Blood.

24. Simon JA, Lange CA (2008) Roles of the EZH2 histone methyltransferase in cancer epigenetics. Mutat Res 647: 21-29.

25. Tiwari VK, Cope L, McGarvey KM, Ohm JE, Baylin SB (2008) A novel 6C assay uncovers Polycomb-mediated higher order chromatin conformations. Genome Res 18: 1171-1179.

26. Velichutina I, Shaknovich R, Geng H, Johnson NA, Gascoyne RD, et al. (2010) EZH2-mediated epigenetic silencing in germinal center B cells contributes to proliferation and lymphomagenesis. Blood 116: 5247-5255.

27. Weisenberger DJ, Velicescu M, Cheng JC, Gonzales FA, Liang G, et al. (2004) Role of the DNA methyltransferase variant DNMT3b3 in DNA methylation. Mol Cancer Res 2: 62-72.

28. Wu X, Gong Y, Yue J, Qiang B, Yuan J, et al. (2008) Cooperation between EZH2, NSPc1-mediated histone H2A ubiquitination and Dnmt1 in HOX gene silencing. Nucleic Acids Res 36: 3590-3599.

29. Chen LS, Storey JD (2008) Eigen-R2 for dissecting variation in high-dimensional studies. Bioinformatics 24: 2260-2262.

30. Angrand PO, Apiou F, Stewart AF, Dutrillaux B, Losson R, et al. (2001) NSD3, a new SET domain-containing gene, maps to 8p12 and is amplified in human breast cancer cell lines. Genomics 74: 79-88.

31. Cordaux R, Batzer MA (2009) The impact of retrotransposons on human genome evolution. Nat Rev Genet 10: 691-703.
